# Whole-genome CRISPR screening identifies genetic manipulations to reduce immune rejection of stem cell-derived islets

**DOI:** 10.1016/j.stemcr.2022.08.002

**Published:** 2022-09-01

**Authors:** Elad Sintov, Igor Nikolskiy, Victor Barrera, Jennifer Hyoje-Ryu Kenty, Alexander S. Atkin, Dario Gerace, Shannan J. Ho Sui, Kyle Boulanger, Douglas A. Melton

**Affiliations:** 1 Department of Stem Cell and Regenerative Biology, Harvard Stem Cell Institute, Harvard University, Cambridge, MA, USA; 2 Bioinformatics Core, Department of Biostatistics, Harvard T.H. Chan School of Public Health, Boston, MA, USA; 3 Howard Hughes Medical Institute, Chevy Chase, MD 20815, USA

**Keywords:** T1D, diabetes, immunogenicity, hypo-immunogenicity, chemokine, CXCL10, transplantation, pancreatic islets, beta cells

## Abstract

Human embryonic stem cells (hESCs) provide opportunities for cell replacement therapy of insulin-dependent diabetes. Therapeutic quantities of human stem cell-derived islets (SC-islets) can be produced by directed differentiation. However, preventing allo-rejection and recurring autoimmunity, without the use of encapsulation or systemic immunosuppressants, remains a challenge. An attractive approach is to transplant SC-islets, genetically modified to reduce the impact of immune rejection. To determine the underlying forces that drive immunogenicity of SC-islets in inflammatory environments, we performed single-cell RNA sequencing (scRNA-seq) and whole-genome CRISPR screen of SC-islets under immune interaction with allogeneic peripheral blood mononuclear cells (PBMCs). Data analysis points to “alarmed” populations of SC-islets that upregulate genes in the interferon (IFN) pathway. The CRISPR screen *in vivo* confirms that targeting IFNγ-induced mediators has beneficial effects on SC-islet survival under immune attack. Manipulating the IFN response by depleting chemokine ligand 10 (CXCL10) in SC-islet grafts confers improved survival against allo-rejection compared with wild-type grafts in humanized mice. These results offer insights into the nature of immune destruction of SC-islets during allogeneic responses and provide targets for gene editing.

## Highlights

• IFN pathway induction sets the fate of SC-islets under allogeneic immune challenge
• “Alarm” genes drive immunogenicity of SC-islets
• Genetically modified SC-islets were generated and evaluated for hypo-immunogenicity
• CXCL10 depletion can reduce immune activation and SC-islet graft rejection

## Graphical abstract

**Figure:**
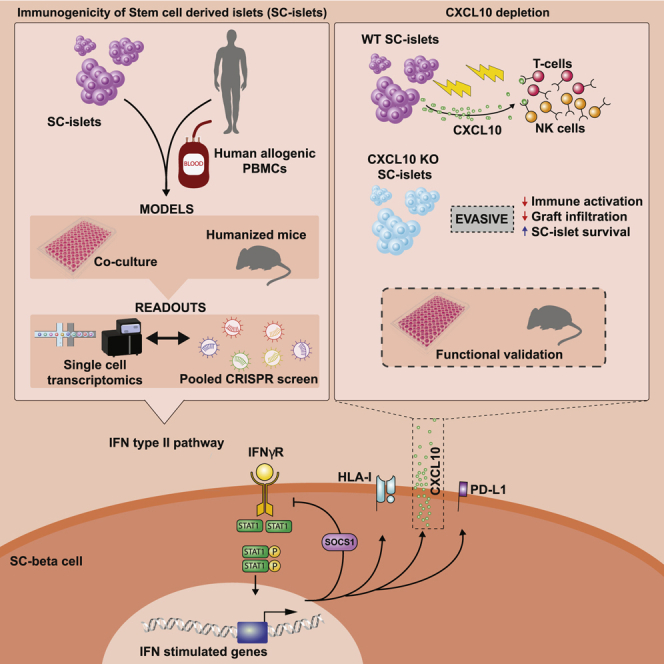

## Teaser

Using single-cell transcriptomics and whole-genome CRISPR screening, Sintov and colleagues find gene perturbation targets that can promote immune evasion of SC-islets in allogeneic transplantations. One of these targets, the early inflammatory response chemokine CXCL10, was depleted in SC-islets, which resulted in partial immune evasion *in vitro* and *in vivo*.

## Introduction

Nearly 100 years ago the first type 1 diabetes (T1D) patient was treated with a “pancreatic extract,” which led to the discovery of insulin (Banting et al., 1922). Since then, the basis of T1D has been shown to be an autoimmune elimination of pancreatic insulin-producing β cells. While acknowledging the impressive technological advances to manage T1D (Kovatchev, 2019), exogenous insulin administration with regular monitoring remains the primary treatment for T1D. In parallel, cadaveric islet or pancreas transplants (Shapiro et al., 2000), have proved to be effective in controlling blood glucose levels, but this treatment is limited by the lack of a consistent and readily available supply of organs/islets and the requirement for systemic immunosuppressants (Shapiro et al., 2017). The prospect of using human pluripotent stem cells (hPSCs) as an unlimited source for β cell differentiation and replacement has been advanced by developing methods to differentiate human stem cells into functional human islets (Helman and Melton, 2021; Nostro et al., 2015; Pagliuca et al., 2014; Rezania et al., 2014; Russ et al., 2015). The first reports of human clinical trials using progenitor cells (Ramzy et al., 2021) or fully differentiated and functional SC-islets (Businesswire, 2021) speak directly to this possibility.

In the light of these encouraging, albeit initial, clinical reports, a major challenge remains of protecting SC-islets from an immune response. The use of immunosuppressants can lead to complications as well as graft impairment in the long term (Lehmann et al., 2008). Encapsulation methods can provide immune protection and graft extraction advantage, but have not yet been determined to be effective (Henry et al., 2018).

Beyond encapsulation, efforts to modify the patient’s immune system have been pursued to blunt or modify the immune response. This includes the use of antibodies to block co-stimulation and amplifying regulatory T cells (Herold et al., 2019; Orban et al., 2011; Raffin et al., 2020). Complementing this approach is genetic modification of the target itself, the SC-islets, to make them opaque or less immunogenic. Strategies include β-2-microglobulin (B2M) or human leukocyte antigen (HLA)-I/II depletions (Castro-Gutierrez et al., 2021; Deuse et al., 2019; Han et al., 2019; Parent et al., 2021; Wang et al., 2015) to prevent donor antigen presentation to T cells, and expression of immune check point inhibitors such as programmed death-ligand 1 (PD-L1) (Castro-Gutierrez et al., 2021; Harding et al., 2019; Yoshihara et al., 2020). Other approaches include expression of CD47 (Deuse et al., 2019, 2021) and HLA-E (Gornalusse et al., 2017) to reduce natural killer (NK) killing when HLA-A, -B, and -C are absent. Another variation is to remove HLA-A and HLA-B but retain one HLA-C allele, requiring only a small number of compatible lines to cover most of recipient populations across the world (Xu et al., 2019). All these promising strategies derive from previous knowledge and studies in other contexts; e.g., maternal-fetal immune interactions and the ability of cancer cells to avoid immune elimination. Of note, there are few reports of endocrine cell-related targets for immune modulation of β cell survival and function (Cai et al., 2020; Wei et al., 2018).

Here we pursue a complementary approach by first defining the immune interaction with SC-islets, studying the interaction between the human allogeneic immune system and SC-islets with a focus on the transcriptional responses. Using single-cell RNA sequencing (scRNA-seq) and whole-genome CRISPR screening, we find that the JAK/STAT type II interferon (IFN) pathway is a leading modulator of early and late inflammatory response events both *in vitro* and *in vivo*. While manipulating the upstream and central mediators of the JAK/STAT pathway provides reduction of SC-islet immunogenicity, the findings indicate that a practical and promising approach is to target downstream components, specifically by depleting the chemokine ligand 10 (CXCL10).

## Results

### Single-cell transcriptional analysis reveals “alarm” genes that drive immunogenicity of SC-islets

**Figure 1:**
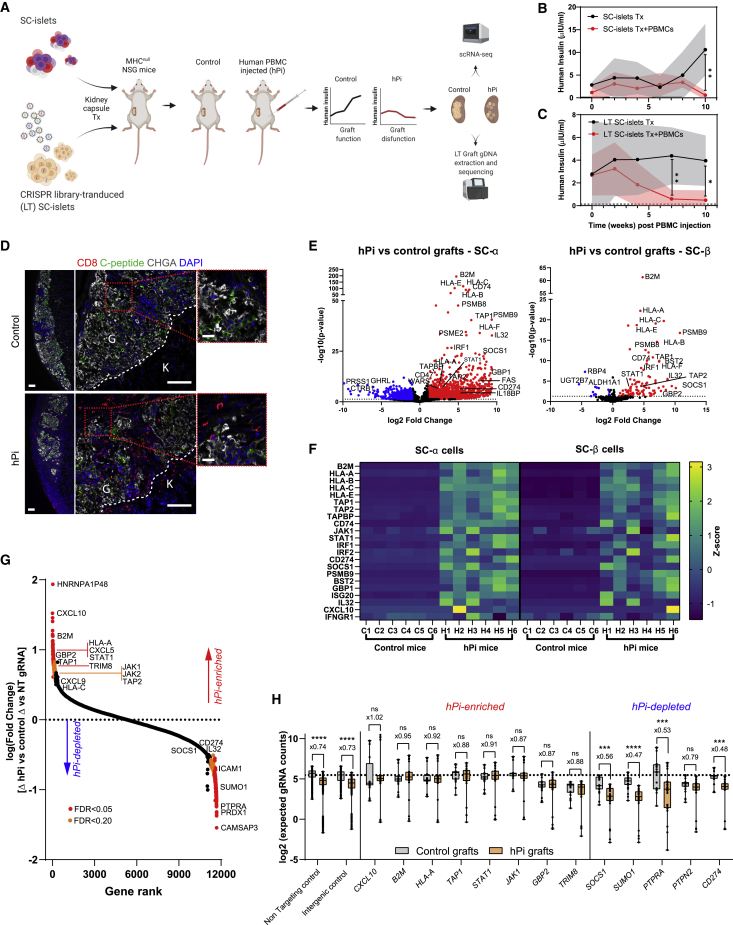
Single-cell transcriptional profile and whole-genome CRISPR screen of SC-islet grafts in an *in vivo* humanized model (A) SC-islets or CRISPR library transduced (LT) SC-islets were transplanted in MHC^null^ NSG mice. Half of each mice cohort was injected with human PBMCs, and human insulin was monitored until graft failure was observed. Grafted cells were then extracted (week 10 post PBMCs) and analyzed by scRNA-seq for gene expression, or by gDNA sequencing for gRNA abundance. (B and C) SC-islet graft failure was assayed in fasted mouse blood by human insulin detection over time, 30 min post glucose. (B) n = 6–8 per group of SC-islet transplanted mice. (C) n = 6 per group of LT SC-islet transplanted mice. (D) Immunofluorescence (IF) staining of kidney SC-islet grafts sections at week 10 after PBMC injection. Bars represent 100 μm in left (×5) and center (×20) and 20 μm in magnified view (right). Kidney (K) and graft (G) margins are outlined. CHGA, chromogranin A. (E and F) scRNA-seq analysis of SC-islet grafts. (E) Volcano plot of differential expressed genes in SC-β and SC-α in hPi versus control grafts. (F) Differential expression of selected genes in different populations, presented as a heatmap. Each row specifies a *Z* score of the specified gene in all graft samples, in the indicated endocrine population. (G) Analysis of enriched and depleted gene KOs. Rank is plotted against fold changes (hPi versus control) of gRNA counts (×4 integrated per gene) relative to integrated non-targeting (NT) gRNA counts (×941). Significant genes are color coded based on false discovery rate (FDR) as indicated. (H) Boxplot presenting individual gRNAs counts (full model predictions) from mice replicates (n = 6 per condition times n = 4 targeting gRNAs, n = 85 for NT gRNAs, or n = 50 for intergenic gRNAs) with genes of interest with positive and negative enrichment in screen. Box lines represent median values. Dashed line represents mean of NT gRNA counts in control mice. Error bars or shaded areas are mean ± SD; ns, not significant; ^∗^p < 0.05; ^∗∗^p < 0.01; ^∗∗∗^p < 0.001; ^∗∗∗∗^p < 0.0001, unpaired two-tailed t test.

To study immune responses in the context of human allogeneic graft rejection, we chose the Hu-PBL-NSG-MHC^null^ humanized mouse (Brehm et al., 2019). NOD-scid IL-2 receptor subunit γ (IL2rg)^null^ (NSG) immunocompromised mice, which lack murine major histocompatibility complex (MHC) class I and II, were transplanted (under the kidney capsule, n = 12) with 5M (Million) SC-islets (HLA-A2 positive), followed by human PBMC injection (termed hPi-mice; 50M/mouse, n = 6) from healthy unmatched donors (HLA-A2 negative). The lack of murine MHC allowed us to monitor the graft function for prolonged durations without the risk of xenogeneic graft-versus-host disease (GVHD). Half of the SC-islet transplanted cohort (n = 6 mice) was used as the control, without PBMC injection (Figure 1A). Graft function failure was determined by human insulin detection in fasting mouse blood 30 min after glucose injection (Figure 1B). Reduction in graft size (Figures S1A) and the loss of function to a glucose challenge are attributed primarily to human T cells retained in mouse tissues (Figures S1B and S1C) for the entire experiment. CD8 cytotoxic T cells can be clearly seen infiltrating the SC-islet grafts (Figure 1D) of hPi-mice mice in week 10 and in proximity to endocrine (chromogranin A+) and SC-β cells (C-peptide+). Note that SC-islets contain several pancreatic hormone-producing cell populations, including glucagon-expressing SC-α and insulin-expressing SC-β. At 10 weeks post PBMC injections, we observed that both SC-α and SC-β numbers are reduced in hPi-mouse grafts (hPi grafts) compared with controls (Figure S1D), as expected for an allogeneic response.

Since graft elimination by PBMCs is incomplete and residual endocrine cells remain in the hPi-mice grafts, we were able to retrieve the SC-islet grafts for single-cell RNA sequencing (scRNA-seq) analysis (Augsornworawat et al., 2020). These samples were used for 10x Genomics mRNA expression library preparation and Illumina sequencing. Datasets were integrated from multiple graft and cell samples (see section “experimental procedures”). As seen in Uniform Manifold Approximation and Projection (UMAP) plots (Figures S1G and S1H), grafted endocrine cells (SC-Endo) from control and hPi-mice maintain their cell identity based on gene markers for SC-α (*INS*−*GCG*+), SC-β (*INS*+*GCG*−), and SC-enterochromaffin cells (SC-EC; *TPH1*+). hPi grafts had fewer endocrine cells (Figure S1I) compared with controls (∼50% reduction), consistent with flow cytometry staining (Figure S1D).

Single-cell technology allows a focus on specific cells populations within heterogeneous SC-islets (Figures S1G–S1I). SC-α, SC-β, and SC-EC exhibited similar patterns of upregulated genes in PBMC infiltrated grafts (Figures 1E, S1J, and Data S1). This suggests that the response in this model system is not specific to a cell population within SC-islets and all transplanted cells are immunogenic.

Among the most upregulated genes are transcripts involved in antigen presentation (*B2M*; *HLA-A*, *-B*, *-C*, *-F*; *TAP1/2*; *CD74*; *PSMB9*), inflammatory pathway mediators (*STAT1*, *JAK1/2*, *IRF1/2*) and pro-inflammatory cytokines, including *IL32*. These genes induce T cell activation and inflammation. In addition, genes that are inhibitory to the immune system are upregulated; e.g. *HLA-E*, *SOCS1*, *CD274* (PD-L1), and *WARS*. Upregulation of these genes suggests an induction of IFN type I (IFNα/β) and II (IFNγ) pathways, through JAK/STAT signaling (Platanias, 2005) (Figures 1E, 1F, S1J, and S1K). A key IFN type II upstream component, the IFNγ receptor gene *IFNGR1*, does not appear to change in hPi-mouse grafts compared with controls (Figure 1F). Pathway analyses confirms the SC-islet response as IFN-driven, one that alarms the immune system through antigen presentation and that can lead to apoptosis of target cells (Tables S1–S3).

### Whole-genome CRISPR screen confirms the role of IFN response genes that set the fate for SC-islet survival

Transcriptional responses of SC-islets during the immune interaction described above provide clues to genes that could be manipulated to dampen immune recognition. However, changes in expression per se might represent a pro- or anti-stimulatory response or no effect. To explore this issue, a whole-genome screen using a CRISPR lentivirus library (Doench et al., 2016) was performed.

The Brunello CRISPR library consists of a pool of 76,441 human targeting guide RNAs (gRNAs) and 1,000 control gRNAs (non-targeting [NT] or intergenic) in a lentiviral vector that expresses Cas9. The pooled library targets 19,114 human genes, most of them by four gRNAs per gene. To avoid multiple different gRNAs in cells and a nonspecific effect on the screen results (Doench, 2018), a low infection lentivirus titer (MOI < 1) was used. Library transduced cells (LT SC-islets) were allowed at least 10 days for CRISPR editing, before transplantation to the NSG-MHC^null^ mouse model, where PBMCs were injected to half of the cohort (hPi-mice, n = 6; control mice, n = 6) (Figure 1A). hPi-mice retained levels of circulating T cells throughout the experiment (Figure S1E). Graft function and subsequent failure due to human PBMC injection was assessed (Figures 1C and S1F). When hPi graft failure was confirmed, 10 weeks after PBMC injection (Figure 1C), both control and hPi grafts were recovered from kidney sites, genomic DNA (gDNA) was extracted, and gRNA regions were amplified by PCR for Illumina sequencing.

The response to PBMCs (graft infiltration) was assessed by gRNA counts from hPi LT SC-islet grafts compared with control LT SC-islet grafts, in relation to NT control gRNA counts in the two environments (see section “experimental procedures”). Essential/housekeeping genes are not evaluated because their gRNA transduced cells will have been eliminated shortly after lentiviral infection. This analysis identifies genes that increase or decrease the chance of transplanted SC-islets survival following PBMC injection (Figure 1G). Approximately 12,000 genes that are expressed in SC-islets (by scRNA-seq datasets) were ranked based on enrichment/depletion following PBMC injection. Results show reduction in total and control (NT or intergenic) gRNA reads in all hPi grafts compared with control, confirming cell elimination and graft rejection (Figures 1H left and S1M). Knockout (KO) perturbations that increase survival are positively enriched in hPi (positive values in Figure 1G) and eliminate the difference in gRNA counts between conditions (Figure 1H center). KO perturbations that decrease survival are depleted in hPi (negative values in Figure 1G) and intensify the difference in gRNA counts between conditions (Figure 1H right). We interpret hPi-enriched gene KOs as pro-survival (tolerizing) under immune attack, whereas the opposite occurs with hPi-depleted genes.

Consistent with expressed transcripts (Figures 1E and 1F), the results point to JAK/STAT signaling for antigen processing/presentation and chemokine secretion. Most prominent were the enrichments of *B2M*, *HLA-A*, *TAP1/2*, *STAT1*, *JAK1/2*, and *CXCL10* gRNAs in LT SC-islet hPi grafts (Figure 1G). KOs of these genes contribute to survival in hPi (Figure 1H).

The observed protective effect of HLA-I KOs is consistent with previous reports (Castro-Gutierrez et al., 2021; Deuse et al., 2019; Han et al., 2019; Parent et al., 2021; Wang et al., 2015). *TAP1* and *TAP2* gRNA enrichments in hPi suggest that immune protection could also be gained by disrupting transport of cytosolic peptides to HLA class I molecules (Scholz and Tampe, 2005).

Interestingly, one of the top hPi-enriched gene perturbations in this screen was for CXCL10 (IP10), an IFN-induced chemokine. Chemokine signaling plays an important role in immune cell recruitment to an inflamed tissue. Other chemokine gRNAs that are hPi-enriched include *CXCL5* and *CXCL9*. *CXCL9* is also an IFN-stimulated gene (ISG) that binds the CXCR3 receptor. *CXCL5* is known to have chemotactic and activating functions on neutrophils (Chang et al., 1994).

Apart from the canonical mediators of the IFN pathway (*STAT1* and *JAK1/2*), other notable hPi-enriched perturbations are *HNRNPA1P48*, *GBP2*, and *TRIM8*. hnRNP proteins are involved RNA processing and splicing (Clarke et al., 2021). *GBP2* is an IFNγ-induced GTPase involved in protective immunity against microorganisms (Tretina et al., 2019) and is also a marker for an efficient T cell response in breast carcinomas (Godoy et al., 2014). TRIM8 is a RING finger protein that inhibits the JAK/STAT suppressor SOCS1 (Toniato et al., 2002), and therefore might act as a IFNγ pathway inducer.

The bottom of Figure 1G shows gene hits that are beneficial to graft survival under immune infiltration of PBMCs. Artificially expressing these genes may help slow or prevent immune destruction. One example is *PTPRA*, a negative regulator of JAK/STAT signaling (Gurzov et al., 2015; Stanley et al., 2015). The difference of *PTPRA* gRNA counts between hPi and control graft is larger than that observed in NT gRNAs, emphasizing the essentiality of PTPRA for graft survival (Figure 1H). Another tyrosine phosphatase, *PTPN2*, is a T1D risk gene (Barrett et al., 2009; Espino-Paisan et al., 2011) but was ranked lower as a beneficial gene in our screen (Figure 1H). In addition, suppressor of cytokine signaling 1 (*SOCS1*), also a negative regulator of JAK/STAT (Galic et al., 2014; Solomon et al., 2011), was upregulated in our scRNA-seq data (Figures 1E and 1F) and exhibited potency as a tolerizing gene (Figure 1H). Other examples that showed a protective effect include small ubiquitin-like modifier 1 (*SUMO1*), which inhibits STAT1 (Rogers et al., 2003), and the tolerizing surface molecule PD-L1 (*CD274*) (Castro-Gutierrez et al., 2021; Yoshihara et al., 2020). *IL32*, *ICAM1*, and *PRDX1* are known to be pro-inflammatory in other systems (Min et al., 2018; Ribeiro-Dias et al., 2017; Yonekawa and Harlan, 2005) and it is unclear why their gRNAs were hPi depleted.

### SC-islets are responsible for early-stage immune cell activation through alarm genes

**Figure 2:**
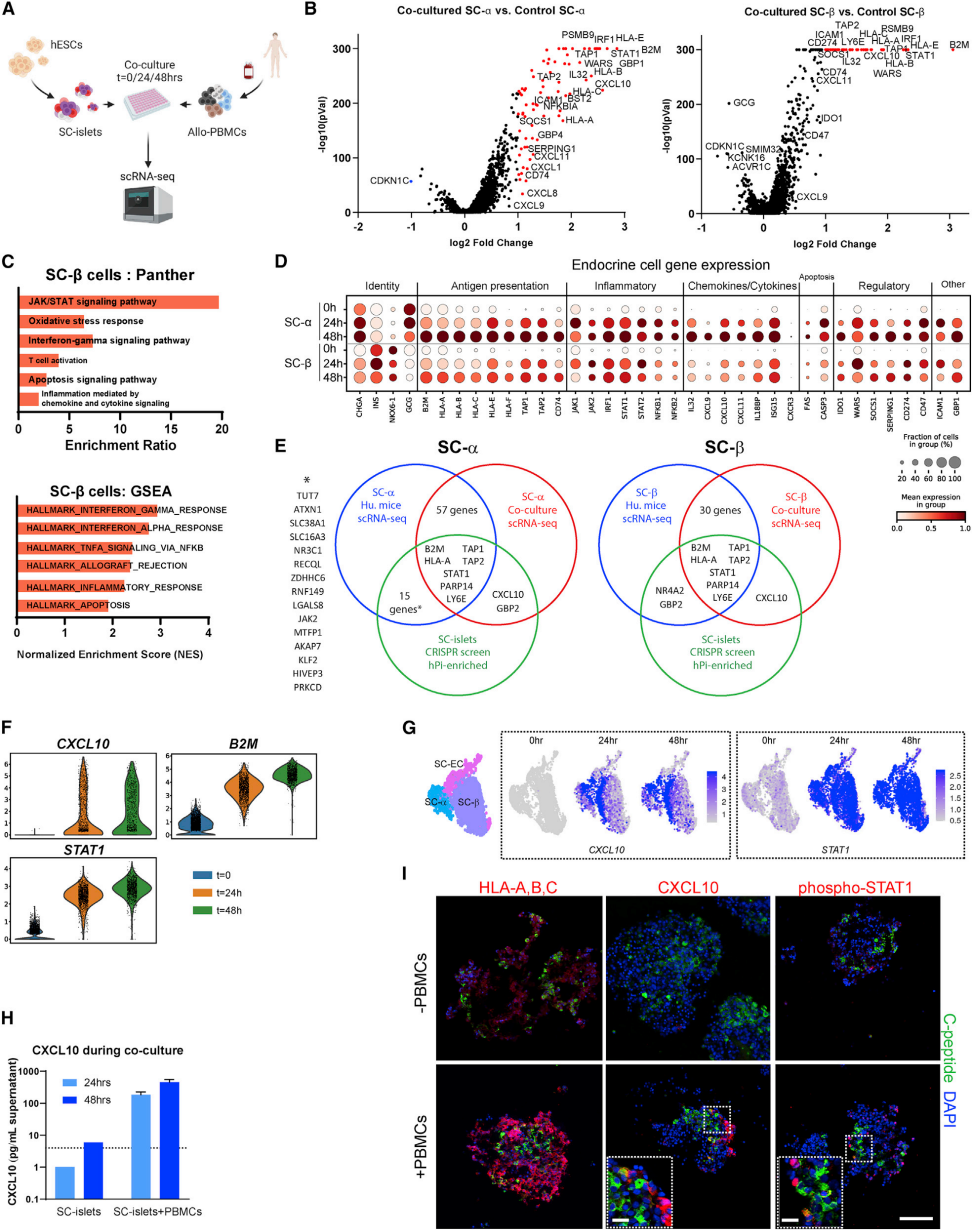
Early response of immune-challenged SC-islets profiled by single-cell transcription analysis after co-culture with human allogeneic PBMCs (A) hESC-derived SC-islets were co-cultured with human allogeneic PBMCs (n = 2 donors) for 0, 24, and 48h, followed by scRNA-seq for gene expression. (B) Volcano plot of differential expressed genes in SC-α or SC-β after 24-h co-culture with PBMCs compared with control (t = 0). (C) Pathway analysis and gene set enrichment analysis (GSEA) of upregulated genes in co-cultured SC-β (48 h). (D) Dot plot representing expression of selected inflammatory genes in groups of SC-α and SC-β over time in co-culture with PBMCs. (E) Venn diagrams feature significantly upregulated genes (log2 fold change >1 and adjusted p values <0.05) obtained from *in vivo* (blue) and *in vitro* (red) SC-α/SC-β scRNA-seq data (Figures 1 and 2) that are common to CRISPR screen hits (positively enriched in hPi-mice, log2 fold change >1) (green). (F) Violin plots of SC-β timed expression of selected genes. See also Figure S2F. (G) UMAP plots of SC-islet cells expressing *CXCL10* or *STAT1* over time in co-culture with PBMCs. Specific endocrine cell type clustering is indicated. (H) ELISA for human CXCL10, from supernatant of co-culture of SC-islets and PBMCs. n = 2 donors. Error bars are mean ± SD. Dashed line is the lower detection limit, while any data below it is extrapolated. (I) IF staining of SC-islet clusters ±48-h co-culture with PBMC. C-peptide staining (green) for SC-β and DAPI (blue) for nuclei. Bars represent 100 μm in main panels and 50 μm in magnified panels.

To compensate for limitations of the hPi-mouse model (Shultz et al., 2019) and for unassessed early events (grafts that are retrieved at week 10), we performed an *in vitro* co-culture of allogeneic PBMCs and SC-islet clusters. SC-islet clusters were enriched for β cells (using CD49A magnetic sorting; SC-α and SC-EC still remain at lower numbers) (Veres et al., 2019), dissociated and reaggregated to obtain a more uniform cell count between wells. SC-islets were co-cultured with human allogeneic PBMCs for 24 or 48 h. As controls (time [t] = 0), SC-islets remained in culture without PBMC addition. These samples, in addition to PBMCs alone (t = 0), were used for scRNA-seq (Figure 2A). Prior to co-culture, all SC-islets (controls included) were treated with thapsigargin to enhance and accelerate T cell activation by inducing an ER stress response that was previously shown to recapitulate aspects of autoimmunity (Leite et al., 2020). Differential expression analysis of integrated data from all samples focused on cell populations of interest (Figures S2A–S2C).

CD4, CD8 T cells, and NK cells, at 24- and 48-h co-culture with SC-islets, displayed gene expression profiles of immune activation compared with control (Figure S2D; Tables S1–S3). Transcripts for T cell co-stimulation molecules (including *CD28*, *CD58* [LFA-3], *CD40LG*, *TNFRSF9* [4-1BB], *TNFRSF4* [OX40]) and other activation markers (*IL2RA* [CD25], *CD38*) are upregulated in T cells as well as inhibitory and exhaustion markers (*HAVCR2* [TIM-3], *LAG3*, *PDCD1* [PD-1]) (Figure S2D top). Co-inflammatory cytokines (*IFNG* and *TNF*) and chemokines (*XCL1/2*) are expressed over time in NK and T cells, while anti-inflammatory cytokines (*IL10* and *TGFB1*) are either undetected or downregulated. T cells and NK sensitization to pro-inflammatory chemokines was increased based on elevated levels of *CXCR3*, a chemokine receptor that binds CXCL9/10/11 (Figure S2D center). Other prominent transcripts are those that play a part in CTL (cytotoxic T lymphocyte) and NK killing functions (Figure S2D bottom: *PRF1*, *GZMB*, *FASLG*), further indications of an allogeneic response in this co-culture system.

We focused on gene expression in SC-α and SC-β cells compared with controls without PBMC addition. Similar to what was observed for the *in vivo* analysis (Figure 1), upregulated profiles did not differ between co-cultured SC-α and SC-β (Figure 2B) and consisted of clear IFN responses through the JAK/STAT pathway with implications for T cell activation (*B2M*, HLA-I genes), inflammation (e.g., *NFKB1/2*), apoptosis signaling (*FAS*, *CASP3*), and allo-rejection (Figures 2C, 2D, S2E and Tables S1–S3; pathway analysis, and gene set enrichment analysis (GSEA)).

The *in vitro* and *in vivo* experiments described (Figures 1 and 2) point to the conclusion that JAK/STAT signaling in SC-islets is a direct and early consequence of IFN signals received from PBMCs. The unbiased whole-genome screening provides further confirmation of IFN signaling as a critical signaling cascade. We compare readouts from these assays in Figure 2E and find seven common genes upregulated in immune-challenged SC-β and SC-α, engrafted or co-cultured. These genes reflect the widely known importance of antigen processing (*TAP1/2*) and presentation (*B2M*, *HLA-A*) by MHC class I in the initiation of immune responses. *STAT1* links the external signal of IFNγ (also IFNα and β) receptors with the downstream effect that consist of MHC-I stimuli, and secreted agents like CXCL10.

*In vivo*, very few SC-islets cells continue to express *CXCL10* at week 10, while other ISGs maintain or increase their levels in both experimental models (Figures 1E, 1F, and S1L). Comparatively, *CXCL10* was one of the top upregulated genes in co-culture, slightly more in SC-α than in SC-β cells (Figures 2B and 2D center and 2F). These results, following the CRISPR screen (Figure 1), provide further evidence that CXCL10 is essential for an IFN-triggered immune response. Other chemokines, *CXCL9* and *CXCL11*, were also upregulated in SC-islets *in vitro*. Chemokine signaling may contribute to the early inflammatory response that was missed due to the graft retrieval timing in our *in vivo* model (Figure 1). It is also possible that CXCL10-expressing cells in the SC-islet grafts are eliminated in the PBMC-injected mice. Regardless, CXCL10 is upregulated in parts of the co-cultured SC-islet endocrine population in scRNA-seq analysis (up to 3-fold in SC-β, high versus low CXCL10 cells) and immunofluorescent staining (Figures 2G and 2I) and can be attributed to IFNγ induction (Figure S2G). Furthermore, higher CXCL10 levels are detected in co-culture supernatants compared with SC-islets only (Figure 2H). In all, CXCL10 appears to have a pivotal role in early alloimmune responses.

Given that the JAK/STAT pathway is highly upregulated in SC-islets during co-culture with PBMCs, we examined genes that activate this pathway, along with the IFNγ receptor, intracellular regulator *STAT1*, negative regulator *SOCS1*, and downstream effectors *B2M* and *CD274* (Figures 2F and S2F). STAT1, a master regulator of the JAK/STAT pathway (Gurzov et al., 2016), is enriched in a GSEA transcription factor motif analysis (Figure S2E). Further evidence for the pathway importance in SC-islet immunogenicity comes from co-culture and external IFN stimuli, wherein STAT1 is phosphorylated and translocated to the nuclei of SC-islet cells, and transcription of IFN response elements are induced (Moore et al., 2011) (Figures 2G, 2I, S2G, and S2H).

### CXCL10 affects SC-islet immunogenicity

**Figure 3:**
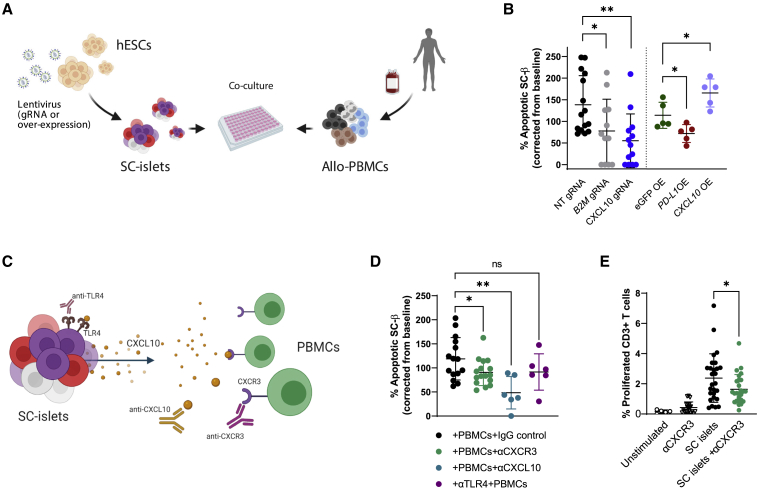
Immunogenicity of CXCL10 expressing SC-islets (A) Transduced SC-islets with lentiviruses carrying Cas9 + gRNA (KO) or a given open reading frame (ORF) insert (overexpression [OE]), were co-cultured with allogeneic PBMCs. (B) Flow cytometry for %TUNEL+ (apoptotic) SC-β cells (C-peptide+), following 48-h PBMC co-culture. Apoptosis was calculated by fraction from baseline (%TUNEL without PBMC). gRNA lentivirus transduced SC-islets were compared with non-targeting (NT) gRNA, and OE transduced SC-islets were compared with eGFP OE. n = 3 for ×5 PBMC donors (left; KO), n = 2–3 for ×2 donors (right; OE). (C) Blocking antibodies prior to/with co-cultures: PBMCs with anti-CXCR3, or SC-islets with anti-TLR4, or anti-CXCL10 during co-culture. (D) Flow cytometry analysis for apoptotic SC-β, following 48-h PBMC co-culture. n = 3 for ×2–6 donors. (E) PBMCs were labeled with cell trace violet (CTV) prior to co-culture. Following a 48-h co-culture, PBMCs were separated and allowed to grow for 7 days. CD3^+^ were gated for the CTV-negative fraction of divided cells. n = 5 for ×3 donors. Error bars are mean ± SD. ns, not significant; ^∗^p < 0.05; ^∗∗^p < 0.01; ^∗∗∗^p < 0.001, unpaired two-tailed t test.

To assess CXCL10 as a target for genetic manipulation compared with other known tolerizing perturbations (β2M KO and PD-L1 overexpression), we co-cultured human allogeneic PBMCs with SC-islets that had been transduced with lentivirus vectors (Figure 3A). For gene KO, vectors expressed Cas9 and gRNAs to *CXCL10* and *B2M*. Overexpression (OE) vectors expressed either *CXCL10* or PD-L1 (*CD274*). All perturbations of target protein expression were assessed compared with NT gRNA or eGFP OE under IFNγ stimuli (Figure S3A). At 48 h after co-culture, SC-islets were stained for apoptotic markers with the focus on SC-β viability (C-peptide staining) (Figure 3B). *CXCL10* and β2M depletions improved viability of SC-β under immune attack by PBMCs (Figure 3B) by more than 2-fold. In addition, a destructive effect of CXCL10 overexpression in SC-β cells under immune attack can be seen by the 50% increase of apoptosis in SC-β overexpressing CXCL10, compared with eGFP overexpression (and comparable with PD-L1) (Figure 3B). PBMCs, pre-labeled with cell trace violet to measure proliferation rates, showed reduced T cell proliferation when co-cultured with CXCL10-depleted SC-islets, compared with NT (Figure S3B). Reduced CXCL10 secretion in CXCL10 KO co-cultures was observed (Figure S3C).

CXCR3 is a chemokine receptor expressed on T helper cells, CD8 T cells, NK cells, and monocytes that react with IFN-inducible chemokines, CXCL9/10/11. CXCR3 has a role in chemotaxis and cell proliferation signals (Loetscher et al., 1996) and can also influence T cell polarization to a specific effector linage (Wildbaum et al., 2002). To evaluate the CXCL10-CXCR3 interaction in SC-islet immunogenicity, PBMC and SC-islet co-culture experiments were performed with a blocking antibody to CXCR3 (Figure 3C). Anti-CXCR3 Ab treatment prior to co-culture with SC-islet reduced T cell activation (CD25 and CD69 activation marker staining), proliferation and the subsequent SC-β apoptotic effect (Figures 3D, 3E, and S3D). An anti-CXCL10 neutralizing antibody added during co-culture also improved SC-β viability (Figure 3D). Since CXCL10 is thought to induce apoptosis through binding to Toll-like receptor 4 (TLR4) in β cells (Schulthess et al., 2009), we treated SC-islets (pre-co-culture) with a TLR4 blocking antibody, which did not significantly reduce apoptosis in this assay (Figure 3D). Overall, these results point to T cell-mediated SC-islet killing through CXCR3 induction, led by CXCL10.

### Immunogenicity of *CXCL10* and *STAT1* KO hESC lines assessed *in vitro*

In the light of aforementioned results, two Hues8 hESCs CRISPR KO lines, *CXCL10* KO and *STAT1* KO, were generated with the rationale of diminishing IFN signaling through a master regulator (STAT1) or by confining the effect to one downstream mediator (CXCL10).

**Figure 4:**
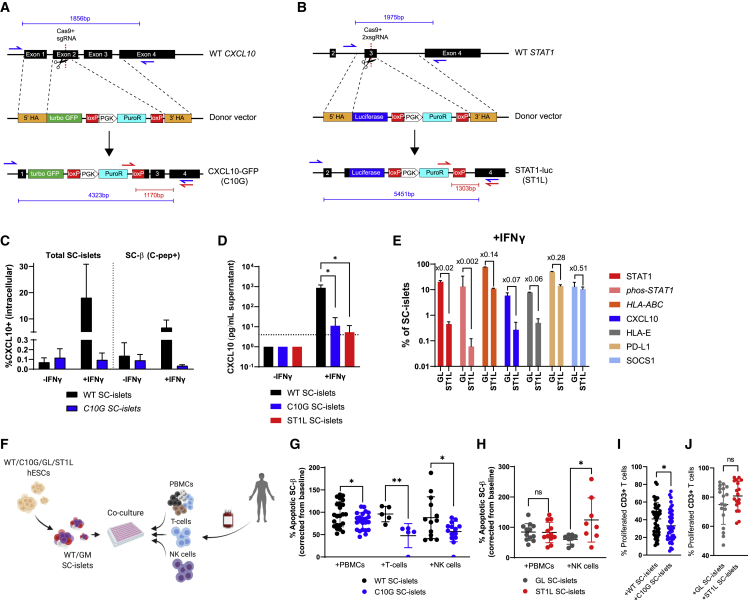
Generation and performance of *CXCL10* KO and *STAT1* KO hESC lines (A and B) Scheme of targeting the (A) CXCL10 or (B) STAT1 locus in hESCs using CRISPR. Red and blue arrows are PCR primers for genotyping as shown in Figure S4A. (C) Flow cytometry of intracellular CXCL10 protein in WT/C10G SC-islets and SC-β (C-peptide+) ± rhIFNγ for 48 h n = 3–5. (D) CXCL10 ELISA of supernatants from ±rhIFNγ-treated WT/C10G/ST1L SC-islets. Dashed line is the lower detection limit, while any data below it is extrapolated. (E) Flow cytometry for protein expression in rhIFNγ-treated GAPDH-luciferase (GL) or ST1L SC-islets. n = 3–4. (F–J) Gene-modified (GM; C10G/ST1L) and control (WT/GL) lines were differentiated into SC-islets, and co-cultured with human PBMCs or purified T cells/NK cells. Apoptosis was calculated by fraction from baseline (%TUNEL without PBMCs). (G) Apoptotic WT or C10G SC-β cells (n = 4 for ×6 PBMC donors, n = 2–3 ×2 T cell donors, n = 4 × 4 NK cell donors). (H) Apoptotic GL or ST1L SC-β cells (n = 4 for ×2 PBMC or NK cell). (I and J) Proliferated CD3 T cell following co-culture with indicated GM SC-islets (I) n = 9 for ×5 donors and (J) n = 9 for ×2 donors). Error bars are mean ± SD. ns, not significant; ^∗^p < 0.05; ^∗∗^p < 0.01, unpaired two-tailed t-test.

Null mutations were created for CXCL10-GFP (C10G) and STAT1-luciferase (ST1L) lines by homology directed repair (HDR) (see section “experimental procedures;” Figures 4A, 4B, and S4A). KO lines displayed normal karyotypes (Figure S4B) and pluripotency marker expression (Figure S4C). These KO lines were compared with a wild-type (WT) Hues8 line or a luciferase expressing Hues8 line (GAPDH-luciferase [GL]; (Gerace et al., 2021)) as controls. C10G, ST1L, and control lines were differentiated successfully into SC-islets (Pagliuca et al., 2014; Veres et al., 2019) and exhibited glucose-stimulated insulin secretion (GSIS) in transplanted mice (Figures S4D–S4F).

C10G SC-islets had very low levels of intracellular CXCL10 staining and almost undetectable CXCL10 secretion after IFNγ stimulation (Figures 4C and 4D). IFNγ treatment of GL SC-islets induced phosphorylated STAT1 that was impaired in ST1L SC-islets (Figure 4E). The absence of STAT1 in ST1L also led to desensitization to IFNγ, as shown by the downregulation of HLA proteins and CXCL10 as well as inhibitory proteins (HLA-E, PD-L1, and SOCS1) (Figures 4D and 4E).

Gene-modified (GM) and control SC-islets were co-cultured with allogeneic PBMCs. To evaluate the contribution of specific immune populations on SC-islet killing, we also co-cultured GM SC-islets with blood purified T cells (C10G only) and NK cells (Figure 4F). Compared with WT, C10G co-cultures displayed significant protective performances against allo-PBMCs, T cells, and NK cells based on improved SC-β (Figure 4G) and SC-islet (Figure S4G) viability and reduced activation and proliferation of T cells (Figures 4I and S4H) in co-cultured PBMCs. In contrast, ST1L did not significantly reduce the response to PBMCs, and more SC-islets were apoptotic after NK cell co-culture (Figures 4H and S4G). T cells from ST1L and GL control SC-islets, co-cultured with PBMCs, show the same level of activation and proliferation (Figures 4J and S4I). Diminished inhibitory signals such as PD-L1 and SOCS1 (Figure 4E) may explain why ST1L does not reduce the immune response to PBMCs, and the reduced expression of HLA class I may be the cause for increased NK killing (Figures 4H and S4G).

### CXCL10-deficient SC-islets are hypoimmunogenic *in vivo*

Since full *STAT1* depletion (ST1L) shows unimpressive results in reducing the immune response *in vitro* (Figures 4H and 4J), we focused on C10G for *in vivo* studies.

**Figure 5:**
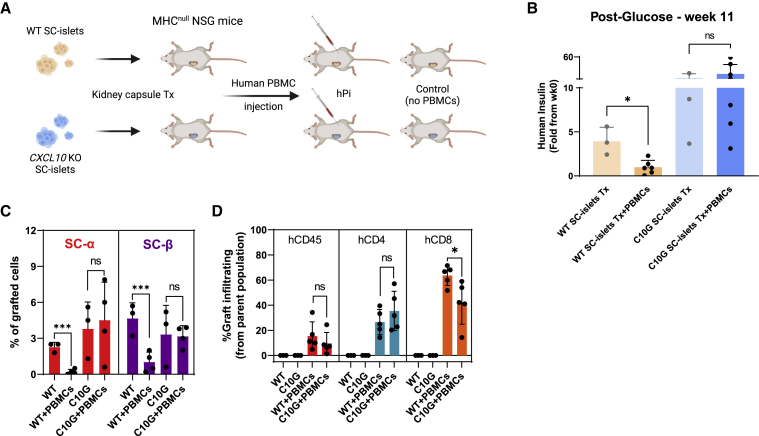
*CXCL10* KO SC-islet grafts evade alloimmune attack in humanized mice (A) WT or C10G SC-islets were transplanted into MHC^null^ NSG mice (n = 10 from each line). n = 6–7 mice from each group injected with human PBMCs (n = 2 human donors), while the remainder served as control (n = 3 per group). (B) Graft failure at week 11 after PBMC injections, as measured by human insulin in fasted mice plasma, 30 min after glucose injection to fasted mice. Data presented as fold increase from t = 0 before PBMC injections. (C) Flow cytometry of SC-α (glucagon+/C-peptide−) and SC-β (glucagon−/C-peptide+) in extracted grafts at week 18 post PBMC injection. n = 3–4 mice per group. (D) Flow cytometry of human T cells in hPi-mouse graft infiltrating at week 18 post PBMC injection. n = 3–5 mice per group. Error bars are mean ± SD. ns, not significant; ^∗^p < 0.05; ^∗∗^p < 0.01, unpaired two-tailed t test.

Using the *in vivo* model (Figure 1), C10G or WT SC-islets were transplanted (n = 20), followed by PBMC injection (hPi) from two human donors, leaving three mice in each group without PBMC injection as controls (Figure 5A). Beginning at week 11 after PBMC injection, graft failure was observed in hPi-mice transplanted with WT SC-islets, continuing through week 17, whereas WT control grafts remained functional. Interestingly, C10G SC-islet graft insulin levels remained stable and even increased over time, with no significant difference between hPi and control mice (Figures 5B and S5A). At the end of the experiment (week 17 post PBMC), kidney capsule grafts were extracted and stained for endocrine and T cell markers. Consistent with insulin measurements (Figure 5B), we observed a decline in the number of SC-β (and SC-α) in WT hPi grafts, but not in C10G hPi grafts compared with controls (Figure 5C). The improved survival of SC-islets can be attributed to the lower frequency of infiltrating human CD8 T cells, comparing C10G hPi grafts with WT hPi grafts (Figure 5D), while circulating human lymphocyte levels did not change (Figure S5B).

In all, SC-islets with impaired ability to express CXCL10 are not only hypoimmunogenic *in vitro* (Figure 4) but are also cable of evading immune attack *in vivo* within an allograft.

## Discussion

This study used two approaches to reveal genes that drive SC-islet immunogenicity: transcript analysis characterized the responses to immune challenge, and CRISPR genome screening helped assess the cause of those responses.

In responding to allogeneic immune cells, the strongest effect in SC-islets is upregulation of ISGs. The results show that T cells are activated in immune environments and express IFNγ, among many other inflammatory genes. The secreted IFNγ leads to an inflammatory cascade in which ISGs are upregulated in SC-islets. A plausible explanation for T cell activation is by antigen presentation through MHC class I molecules.

The most striking observation was the involvement of chemokines secreted by SC-islets. These results suggest that CXCL10 has a role in the early stage of immune-graft interaction. *CXCL10*-KO SC-islet cells in an allogeneic *in vivo* model survived longer compared with surrounding cells with other perturbations. Furthermore, the *in vitro* and *in vivo* allogeneic experiments demonstrate that CXCL10-deficient SC-islets are immune evasive compared with WT. *CXCL10*-deficient SC-islets (C10G) have 2-fold increased survival capability under immune challenge by allogeneic T cells or NK cells. Upon engraftment in a humanized allogeneic *in vivo* model, C10G avoid immune destruction 7 weeks longer than WT SC-islets. CXCL10 as a secreted chemokine plays a determining role as a recruiter of immune cells to an SC-islet transplant site, and depleting it keeps those grafts out of the reach of a human immune system.

CXCL10 is one of the most upregulated chemokines in primary human islets (Eizirik et al., 2012) and hPSC-derived islets (Demine et al., 2020; Dettmer et al., 2022) under pro-inflammatory conditions. Islets of recent-onset T1D show CXCL10 expression in regions where infiltrating lymphocytes express CXCR3 (Roep et al., 2010; Uno et al., 2010). Our results show that CXCL10 expression is not exclusive to SC-β cells but is also differentially expressed by other SC-endocrine cells. A recent study also demonstrated the contribution of pancreatic α cells to CXCL10 expression in NOD mice and in recent-onset T1D islets (Nigi et al., 2020).

In our previous study using a T1D autologous *in vitro* model, CXCL10 was highly secreted from iPSC-islets during co-culture with matched T1D PBMCs (Leite et al., 2020). In this current work, CXCL10 expression was seen in co-cultures but not in late stages of graft rejections, supporting the view of CXCL10 as a first responder or alarm protein at the onset of SC-islet interactions with a hostile immune system. In T1D, islet CXCL10 expression occurs in early stages (Roep et al., 2010; Uno et al., 2010) and serum levels of CXCL10 are elevated in recent-onset compared with long-term T1D individuals (auto-Ab+) (Shimada et al., 2001). Mouse islet isografts expressed high levels of Cxcl10 at day 2 after transplantation into diabetic C57BL/6 mice, but in a lesser degree by day 100 (Bender et al., 2017). Furthermore, analysis of plasma samples from human islet transplant patients revealed that CXCL10 was among the highest released inflammatory mediators and peaked 24 h post transplantation (Yoshimatsu et al., 2017).

In T1D, pancreatic islets react to pro-inflammatory cytokines by inducing the NF-κB and STAT1 signaling that contribute to the immune destruction mechanism of β cells (Cnop et al., 2005; Eizirik et al., 2012). Although our experiments were done in an allogeneic setting, both transcription factors were upregulated in SC-islets, but only *STAT1* depletion showed up as a hit in the CRISPR screen. However, when *STAT1* KO (ST1L) SC-islets were used, this rescue was not reproduced (Figure 4). The reason might derive from the observation that STAT1-deficient SC-islets lose immune-inducing elements such as HLA molecules and CXCL10, but also suffer from loss of immune-inhibitory functions like PD-L1 and SOCS1.

Downstream to STAT1 is the transcription factor IRF1, which has anti-inflammatory effects in β cells through the induction of SOCS1 (Moore et al., 2011). SOCS1 and PTPN2 are negative regulators of cytokine signaling (Chong et al., 2002; Elvira et al., 2022; Moore et al., 2009) and are both associated with T1D risk loci (Onengut-Gumuscu et al., 2015; Ram and Morahan, 2017). Previous reports have shown that SOCS1 overexpression in NOD mice islets prevent diabetes (Flodstrom-Tullberg et al., 2003), and delays allogeneic islet graft rejection in mouse models (Solomon et al., 2011). Our data show that, under PBMCs + SC-islet interactions, both *IRF1* and *SOCS1* are differentially upregulated. *SOCS1* KO and *PTPN2* KO SC-islets were depleted in hPi grafts in our CRISPR screen, along with *PTPRA* KO, another PTP family member (Stanley et al., 2015).

Based on ST1L’s unconvincing results (Figure 4), “pan-JAK/STAT” diminishing strategies should be considered cautiously. These approaches include *SOCS1* overexpression and *IFNGR1* KO. Transgenic lines of *SOCS1* OE or *IFNGR1* KO might have consequences of losing the inflammatory negative regulation feedback of JAK/STAT signaling. PD-L1 downregulation under JAK/STAT silencing will expose SC-islets to T cell attack, while HLA downregulation will result in NK cell recognition and killing. It may be useful to co-edit such stem cell lines with additional modification(s) that will address these concerns.

The analyses presented in this paper include many other genes that may be targeted to control the immune response against SC-islets. Modulation of ISGs by identified hits from our *in vivo* CRISPR screen (e.g., *TRIM8*, *SUMO1*), or others of unclear function (e.g., *IL32*, *CAMSAP3*), were not considered here but may have the potential to reduce immunogenicity. Nevertheless, this study points to opportunities for future applications of SC-islet as a cell replacement therapy for T1D.

### Limitations of the study

An optimal pooled screen would be one that relies on a robust assay with a selection force that separates cells using a phenotype of interest (Doench, 2018). Although we were able to acquire gene hits from the described *in vivo* CRISPR screen, the assay (hPi model) is not flawless. T cells are the only immune cells that engraft successfully and persist long term, leaving out other immune cells that may also contribute to SC-islet graft destruction, in particular NK cells (Shultz et al., 2019). In addition, pooled screens can benefit from survival selection of cells that could proliferate and amplify the enrichment signal. The enrichment in our screen is based solely on differentiated post-mitotic cells.

## Experimental procedures

### Contact for reagent and resource sharing

Further information and requests for resources and reagents should be directed to and will be fulfilled by the corresponding author, Douglas A. Melton (dmelton@harvard.edu).

### Experimental model and subject details

All procedures were performed in accordance with the Institutional Review Board (IRB) guidelines at Harvard University under IRB and Embryonic Stem Cell Research Oversight Committee (ESCRO) protocol E00024. All animal experiments were performed in accordance with Harvard University International Animal Care and Use Committee regulations.

### Quantification and statistical analyses

Statistical analysis was performed by unpaired Student’s t tests as indicated, using Prism v9. All data are presented mean ± SD. p < 0.05 was considered statistically significant. Sufficient sample size was estimated without the use of a power calculation. Data analysis was not blinded.

### Graphic illustrations

Graphic illustrations in the manuscript were created with BioRender.com under BioRender’s academic license terms.

## Author contributions

E.S. conceived the study. E.S., J.HR.K., A.S.A., and K.B. performed the experiments. D.G. was involved in the experimental design and provided technical support. I.N., V.B., and S.H.S. analyzed the scRNA-seq data. I.N. and E.S. analyzed the CRISPR screen data. E.S. and D.A.M. wrote the manuscript. D.A.M. designed and supervised the research.

## Data Availability

scRNA-seq and pooled CRISPR screen data generated during this study are available at NCBI (GEO: GSE200104) and are composed of listed sub-series related to specific experiments described in this paper.

## Supplemental information

Document S1. Figures S1–S5 Tables S1–S3 and supplemental experimental procedures

Data S1. Gene expression and enrichment

Data S2. Single-cell RNA seq QC values

Document S2. Article plus supplemental information
