# Supplementary material for: Whole-genome CRISPR screening identifies genetic manipulations to reduce immune rejection of stem cell-derived islets
Source: Stem Cell Reports. 2022 Sep 1;17(9):1976–90. doi: 10.1016/j.stemcr.2022.08.002 (PMC9481918; doi:10.1016/j.stemcr.2022.08.002)
Supplement: Document S1. Figures S1–S5 Tables S1–S3 and supplemental experimental procedures [file mmc1.pdf]

Stem Cell Reports, Volume *17*

Supplemental Information

Whole-genome CRISPR screening identifies genetic manipulations to reduce immune rejection of stem cell-derived islets

Elad Sintov, Igor Nikolskiy, Victor Barrera, Jennifer Hyoje-Ryu Kenty, Alexander S. Atkin, Dario Gerace, Shannan J. Ho Sui, Kyle Boulanger, and Douglas A. Melton

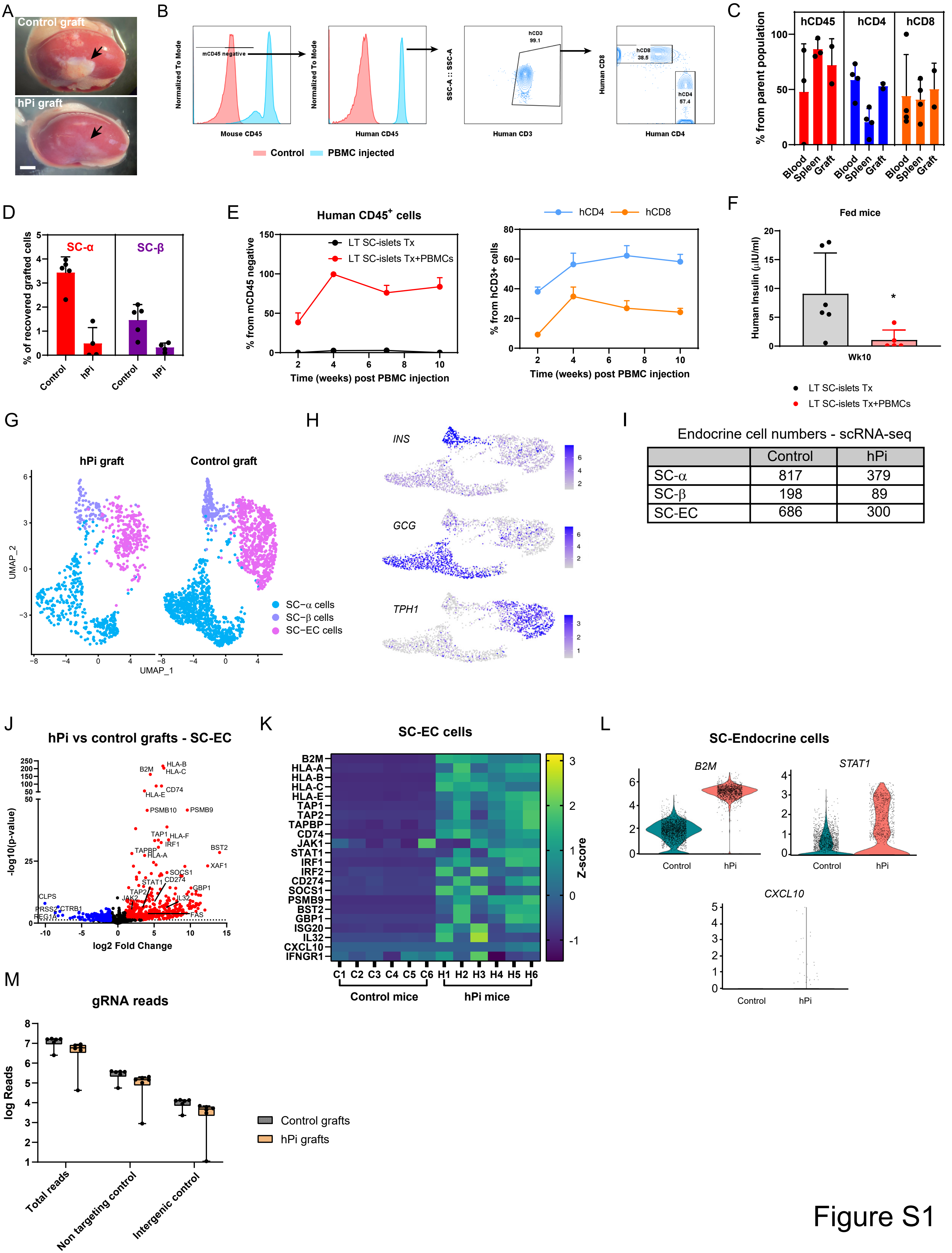
A
Control graft
hPi graft
B
mCD45 negative
Normalized To Mode
Mouse CD45
Human CD45
SSC-A :: SSC-A
Human CD3
hCD3
99.1
Human CD8
Human CD4
hCD8
38.5
hCD4
57.4
Control
PBMC injected
C
hCD45
hCD4
hCD8
% from parent population
Blood
Spleen
Graft
D
SC-α
SC-β
% of recovered grafted cells
Control
hPi
E
Human CD45+ cells
LT SC-islets Tx
LT SC-islets Tx+PBMCs
% from mCD45 negative
Time (weeks) post PBMC injection
hCD4
hCD8
% from hCD3+ cells
F
Fed mice
Human Insulin (µIU/ml)
Wk10
G
hPi graft
Control graft
UMAP_2
UMAP_1
SC−α cells
SC−β cells
SC−EC cells
H
INS
GCG
TPH1
I
Endocrine cell numbers - scRNA-seq
	Control	hPi
SC-α	817	379
SC-β	198	89
SC-EC	686	300
J
hPi vs control grafts - SC-EC
-log10(p-value)
log2 Fold Change
K
SC-EC cells
B2M
HLA-A
HLA-B
HLA-C
HLA-E
TAP1
TAP2
TAPBP
CD74
JAK1
STAT1
IRF1
IRF2
CD274
SOCS1
PSMB9
BST2
GBP1
ISG20
IL32
CXCL10
IFNGR1
Z-score
C1 C2 C3 C4 C5 C6 H1 H2 H3 H4 H5 H6
Control mice
hPi mice
L
SC-Endocrine cells
B2M
STAT1
CXCL10
Control
hPi
M
gRNA reads
log Reads
Total reads
Non targeting control
Intergenic control
Control grafts
hPi grafts

Figure S1

Figure S1. Single cell transcriptional profile and whole genome CRISPR screen of SC-islet grafts in an *in vivo* humanized model. Related to Figure 1.

(A) Representative images of transplanted kidneys after 10 weeks with or without PBMC injection. Bar=2mm.

(B) Gating strategy used for flow cytometry of hPi/control mice blood to detect human T lymphocytes. %Human CD45+ are gated from mouse CD45 negative population. %CD4+ and %CD8+ are gated from hCD45+/hCD3+.

(C) Frequency of human T-lymphocytes in hPi mouse tissues, by flow cytometry.

(D) Frequency of SC-α (Glucagon+/C-peptide-) and SC-β (Glucagon-/C-peptide+) in recovered from SC-islet grafts, by flow cytometry.

(E) Flow cytometry of human T-lymphocytes in mice blood throughout the experiment, transplanted with library-transduced SC-islets (LT-SC-islets) ±PBMC injections (Control/hPi). %human CD45+ are gated from mouse CD45 negative population. %CD4+ and %CD8+ (only in hPi mice) are gated from hCD45+/hCD3+.

(F) Human Insulin detected by ELISA at the 10th week end point of the experiment, in non-fasted mice. Error bars are mean±SD. *p<0.05, **p<0.01, unpaired two-tailed t-test, LT SC-islets Tx+PBMCs (hPi) compared to the control.

(G) UMAP plots of human graft cells extracted from mice after 10 weeks with or without PBMC injection. Integration of n=6 mice per group. SC-endocrine cell clusters are indicated.

(H) Cluster identification (integration of n=12 mice) of specific SC-endocrine cells by gene markers (SC-β:*INS*+*GCG*-, SC-α: *INS*-*GCG*+, SC-EC: *TPH1*+).

(I) Cell counts of endocrine cell populations from scRNA-seq integrations.

(**J-L**) scRNA-seq analysis of SC-islet grafts. (**J**) Volcano plot of differential expressed genes in SC-EC in hPi vs. control grafts. **(K**) Differential expression of selected genes in SC-EC, presented as a heatmap. Each row specifies a z-score of the specified gene in all graft samples, in the indicated endocrine population. (**L**) Violin plots of selected genes, associated with the IFNγ response, expressed in SC-Endocrine cells.

(**M**) Total or control gRNA reads in CRISPR screen of mice replicates, compared between conditions (±PBMC; n=6 per condition). Box lines represent median values.

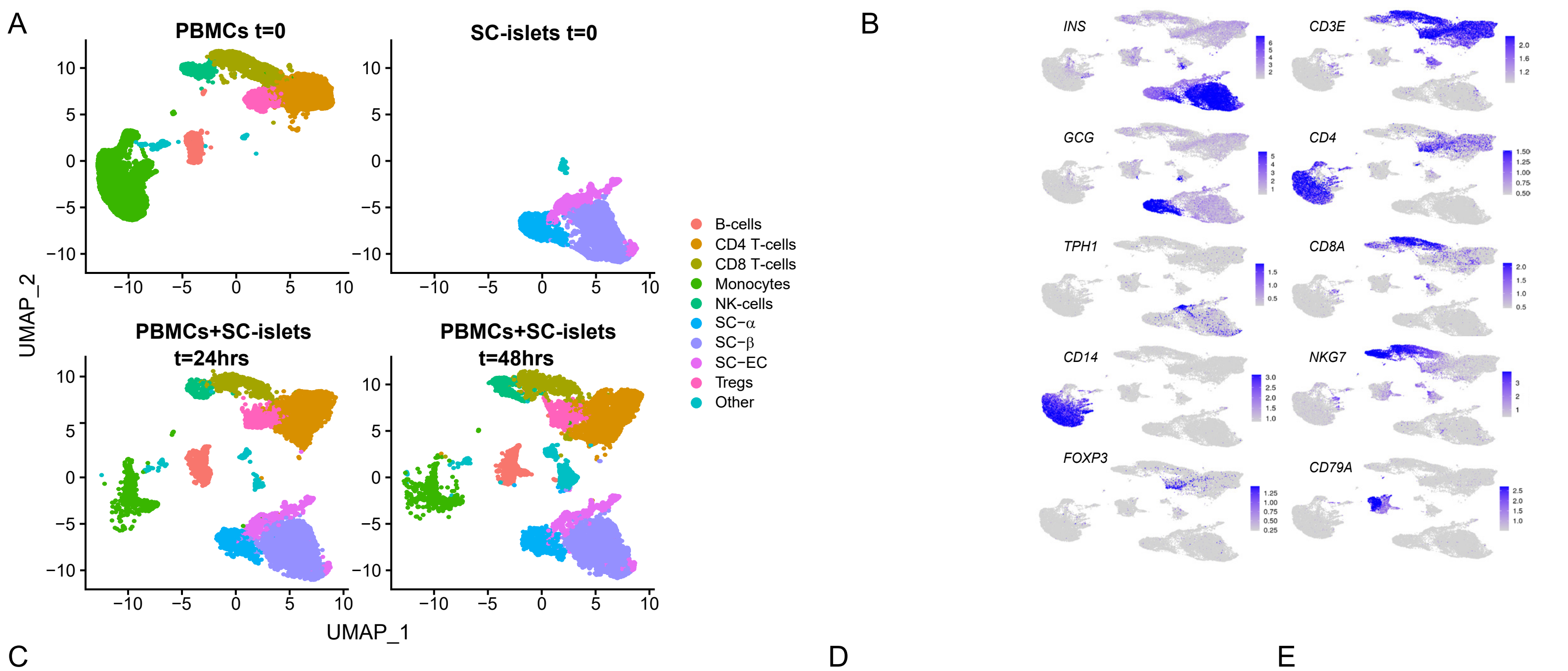

C

Cell numbers - scRNA-seq

	PBMC 0hr	SC-islet 0hr	CC 24hr	CC 48hr
CD4 T-cells	2737	0	2288	1977
CD8 T-cells	2391	0	477	866
B-cells	1140	0	729	859
Monocytes	7725	0	808	681
NK-cells	918	0	152	357
Treg	1217	0	711	654
SC-α	0	1618	877	593
SC-β	0	4723	2672	2006
SC-EC	0	875	573	468
Other	369	0	711	654

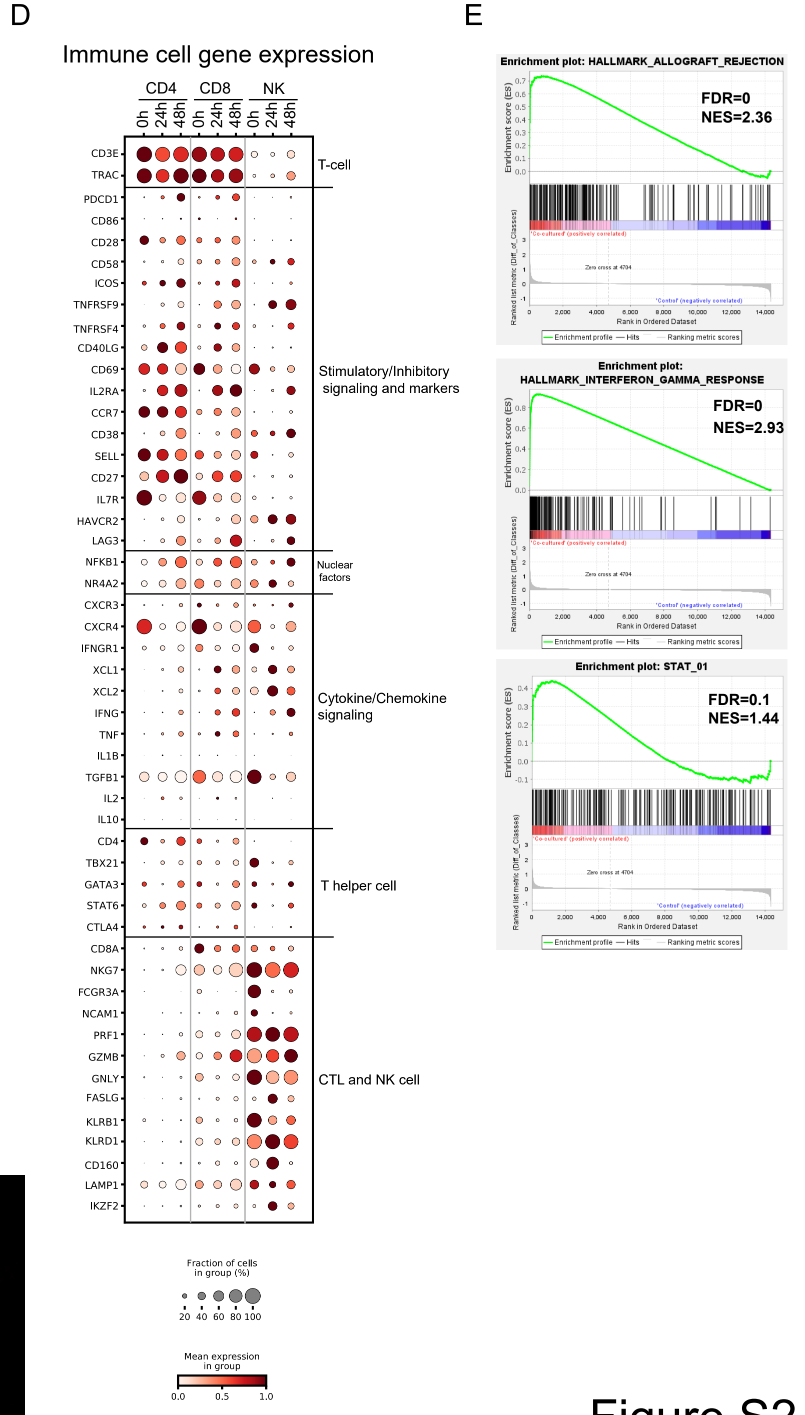

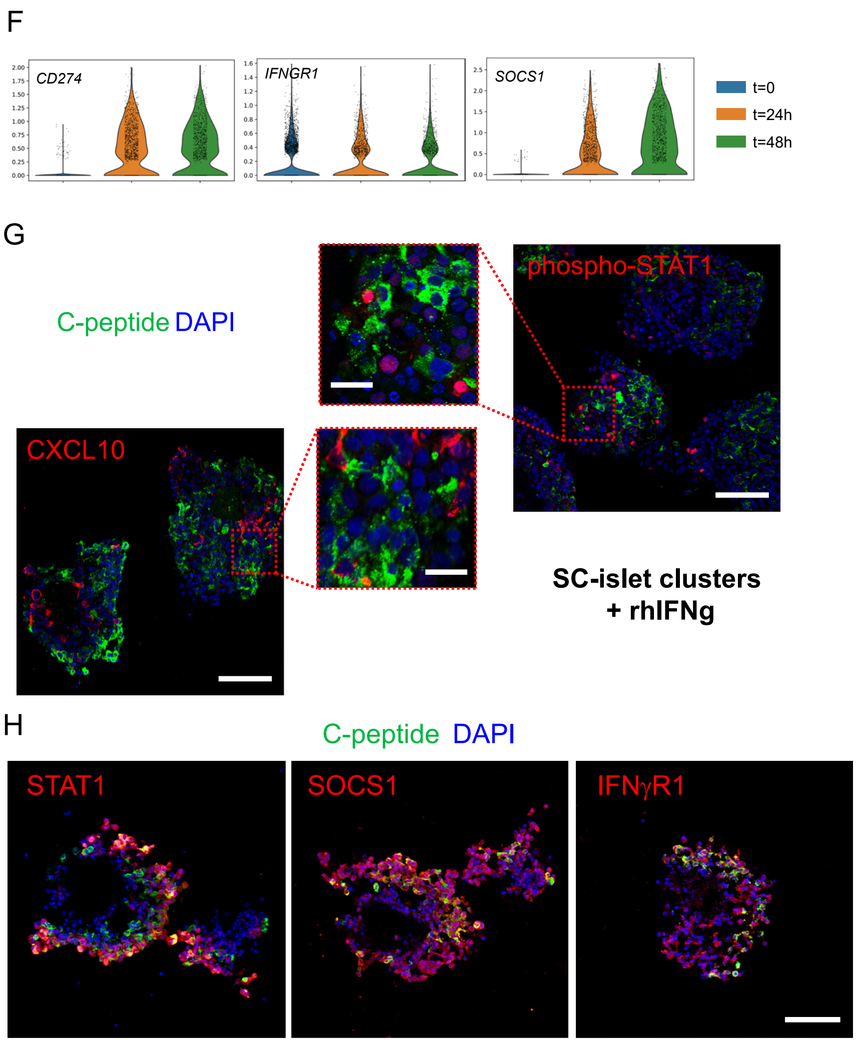

Figure S2

Figure S2. Early response of immune challenged SC-islets profiled by single cell transcription analysis after co-culture with human allogeneic PBMCs. Related to Figure 2.

(**A**) UMAP plots of PBMC+SC-islet co-cultured cells, immune/SC-islet cell clusters are indicated.

(**B**) Cluster identification (integration of all time points) of specific cells by gene markers.

(**C**) Cell counts of cell populations from scRNA-seq integrations. CC=co-culture.

(**D**) Dot plots representing expression of activation/inhibitory genes in specific immune populations, in response to timed SC-islet stimulation.

(**E**) Selected GSEA plots for interferon response and TF motifs, FDR values and normalized enrichment scores (NES) indicated.

(**F**) Violin plots of SC-β timed expression of selected genes.

(**G**) CXCL10 and phosphorylated STAT1 in SC-islet clusters 48hrs after treatment with 20ng/ml rhIFNγ. C-peptide staining for SC-β. Bars are 100μm in main panels and 20μm in magnified panels.

(**H**) IF staining of SC-islet clusters after 48hrs co-culture with PBMC. C-peptide staining (green) for SC-β and DAPI (blue) for nuclei. Bars are 100μm.

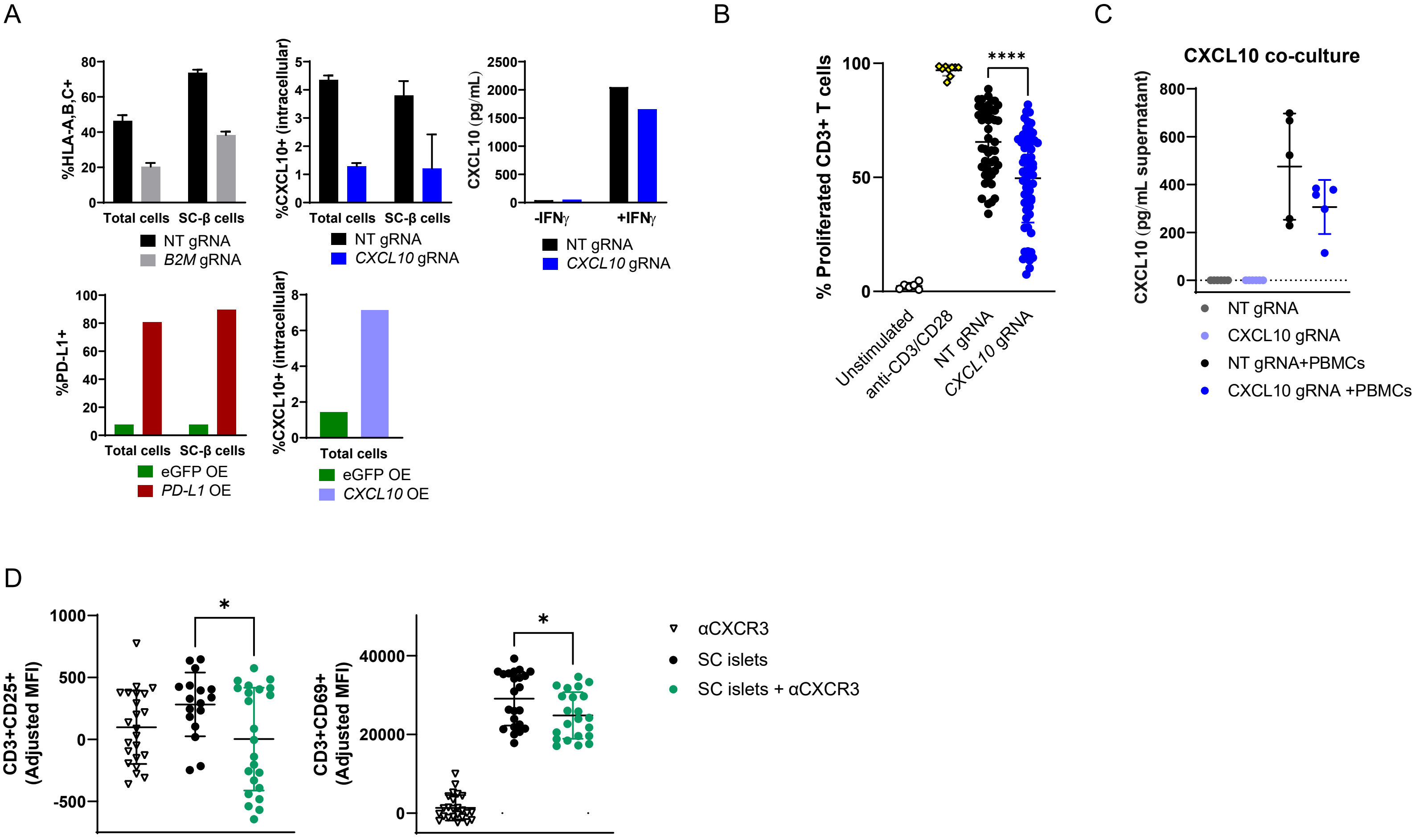
A
%HLA-A,B,C+
Total cells
SC-β cells
NT gRNA
B2M gRNA
%CXCL10+ (intracellular)
CXCL10 gRNA
CXCL10 (pg/mL)
-IFNγ
+IFNγ
%PD-L1+
eGFP OE
PD-L1 OE
CXCL10 OE
B
% Proliferated CD3+ T cells
****
Unstimulated
anti-CD3/CD28
NT gRNA
CXCL10 gRNA
C
CXCL10 co-culture
CXCL10 (pg/mL supernatant)
NT gRNA+PBMCs
CXCL10 gRNA +PBMCs
D
CD3+CD25+ (Adjusted MFI)
CD3+CD69+ (Adjusted MFI)
*
αCXCR3
SC islets
SC islets + αCXCR3

Figure S3

Figure S3. Immunogenicity of CXCL10 expressing SC-islets. Related to Figure 3.

(**A**) Flow cytometry analysis of protein expression of indicated perturbation in SC-islets or specifically in C-peptide+ SC-β 48hrs after rhIFNγ treatment. Where indicated CXCL10 secretion was measured by ELISA.

(**B**) Transduced SC-islets were co-cultured with Cell Trace Violet (CTV) labeled PBMC for 48hrs. PBMCs were then separated and allowed to grow in culture for an additional 7 days, followed by CD3 staining for flow cytometry. CD3+ were gated for the CTV negative fraction of divided cells. PBMCs treated with anti-CD3/CD28 activation beads served as positive control. n=12 for x5 PBMC donors (n=3 for controls).

C) ELISA for human CXCL10, from supernatant of co-culture of SC-islets transduced with NT/CXCL10 gRNA ±PBMCs, n=2-3 for x2 donors. Dashed line is the lower detection limit.

(**D**) Antibody treated (as indicated) SC-islets were co-cultured with PBMC for 48hrs. PBMCs were then separated and analyzed by flow cytometry for CD3+ T cell activation marker expression (CD25 or CD69). n=12 for x2 donors.

NT=Non-targeting, OE=overexpression. Error bars are mean±SD. ns=not significant, *p<0.05, **p<0.01, ***p<0.001, ****p<0.0001 unpaired two-tailed t-test.

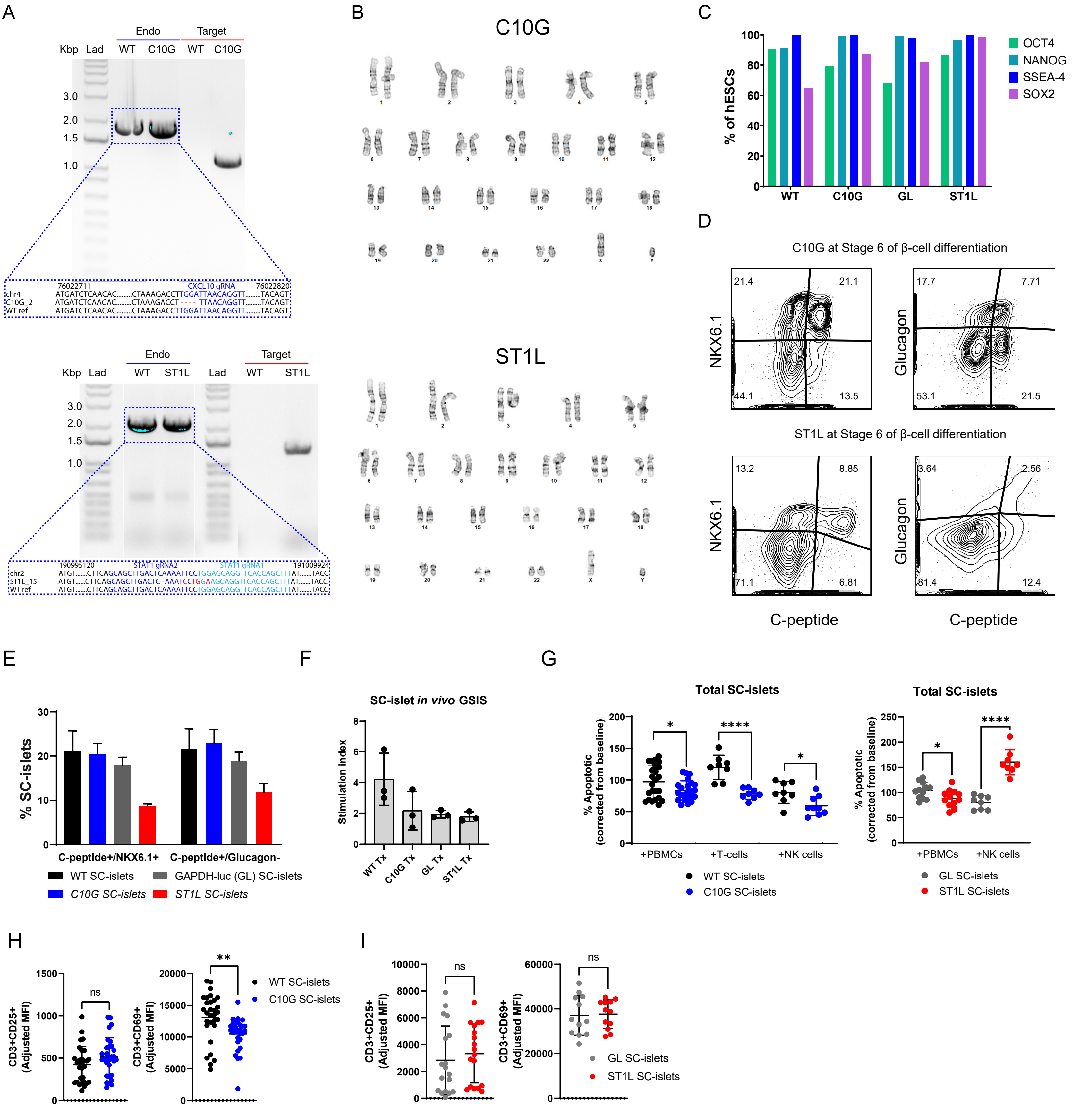
A
Endo
Target
Kbp
Lad
WT
C10G
3.0
2.0
1.5
1.0
CXCL10 gRNA
chr4
C10G_2
WT ref
ST1L
STAT1 gRNA2
STAT1 gRNA1
chr2
STIL_15
B
C10G
ST1L
C
% of hESCs
OCT4
NANOG
SSEA-4
SOX2
GL
D
C10G at Stage 6 of β-cell differentiation
ST1L at Stage 6 of β-cell differentiation
NKX6.1
Glucagon
C-peptide
E
% SC-islets
C-peptide+/NKX6.1+
C-peptide+/Glucagon-
WT SC-islets
GAPDH-luc (GL) SC-islets
C10G SC-islets
ST1L SC-islets
F
SC-islet in vivo GSIS
Stimulation index
WT Tx
C10G Tx
GL Tx
ST1L Tx
G
Total SC-islets
% Apoptotic (corrected from baseline)
+PBMCs
+T-cells
+NK cells
WT SC-islets
C10G SC-islets
GL SC-islets
ST1L SC-islets
H
CD3+CD25+ (Adjusted MFI)
CD3+CD69+ (Adjusted MFI)
ns
WT SC-islets
C10G SC-islets
I
GL SC-islets
ST1L SC-islets

Figure S4

Figure S4. Generation and performance of *CXCL10* KO and *STAT1* KO hESC lines. Related to Figure 4.

(**A**) Clonal genotyping of endogenous or targeted allelles. Endogenous amplified PCR bands were isolated and sequenced for detection of indels, shown in dashed blue frames. Some lanes were cropped to show only relevant clones.

(**B**) Karyotyping analysis of G10G and ST1L hESC cell lines.

(**C**) Pluripotent marker expression by flow cytometry in all 4 lines: wild type (WT), C10G, GAPDH-luc (GL) and ST1L.

(**D**) Flow cytometry analysis to assess %SC-β (%C-peptide+/NKX6.1+ or %C-peptide+/glucagon) in C10G and ST1L hESC at stage 6 of the β-cell differentiation protocol.

(**E**) %SC-β in multiple batches of C10G and ST1L differentiations compared to control WT and GL lines. n=2-3 differentiations.

(**F)** SC-islet GSIS function assay of different lines, 12-15 weeks after transplantation into NSG-mice. Results presented as stimulation ratios of blood human insulin (ELISA) before and 30 min after glucose injection (2g/kg).

(**G**) Flow cytometry was used to assess %TUNEL+ SC-islets. Apoptosis was calculated by fraction from baseline (%TUNEL without PBMC). Left) WT or C10G SC-β cells (n=4 for x6 PBMC donors, n=2-3 x2 T-cell donors, n=4 x2 NK cell donors); Right) GL vs. ST1L SC-β cells (n=4 for x2 PBMC or NK cell donors).

(**H,I**) GM SC-islets were co-cultured with PBMC for 48hrs. PBMCs were then separated and analyzed by flow cytometry for CD3+ T cell activation marker expression (CD25 or CD69). (**H**) n=9 for x3 PBMC donors (**I**) n=9 for x2 PBMC donors

Error bars are mean±SD. ns=not significant, *p<0.05, **p<0.01, ****p<0.0001 unpaired two-tailed t-test.

A

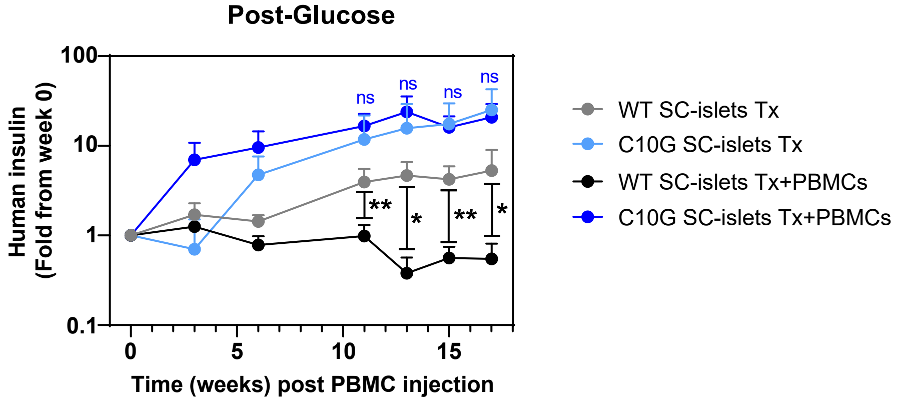
Post-Glucose
Human insulin (Fold from week 0)
100
10
1
0.1
0
5
10
15
Time (weeks) post PBMC injection
ns
ns
ns
ns
**
*
**
*
WT SC-islets Tx
C10G SC-islets Tx
WT SC-islets Tx+PBMCs
C10G SC-islets Tx+PBMCs

B

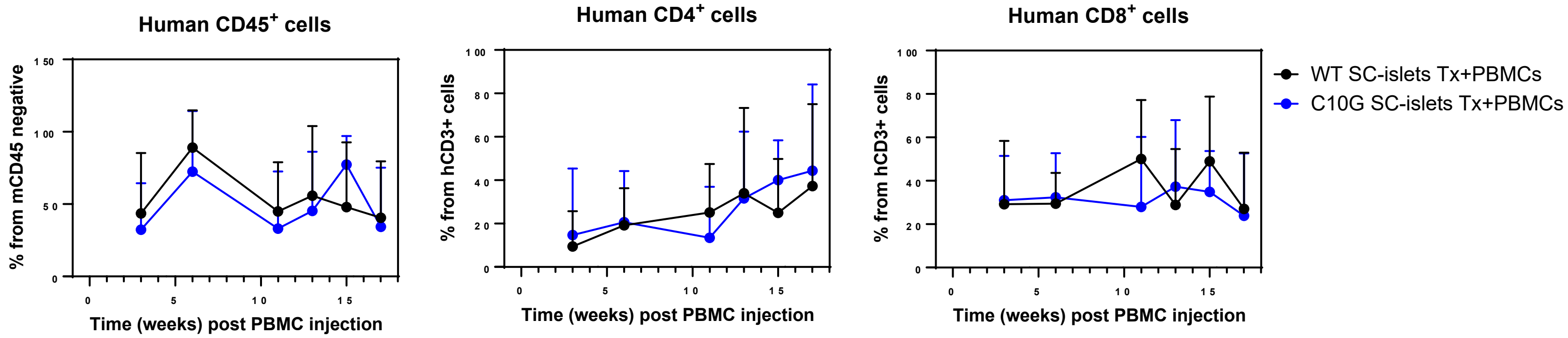
Human CD45+ cells
% from mCD45 negative
Time (weeks) post PBMC injection
Human CD4+ cells
% from hCD3+ cells
Time (weeks) post PBMC injection
Human CD8+ cells
% from hCD3+ cells
Time (weeks) post PBMC injection
WT SC-islets Tx+PBMCs
C10G SC-islets Tx+PBMCs

Figure S5

Figure S5. *CXCL10* KO SC-islet transplantation in humanized mice. Related to Figure 5.

(**A**) Graft failure continuously monitored over time after PBMC injections, as measured by human insulin (ELISA) in fasted mice plasma, 30 min after glucose injection to fasted mice. Data presented as fold increase from t=0 before PBMC injections.

(**B**) Flow cytometry of human T-lymphocytes in mice blood throughout the experiment, transplanted WT/C10G SC-islets +PBMC injections. %human CD45+ are gated from mouse CD45 negative population. %CD4+ and %CD8+ (only in hPi mice) are gated from hCD45+/hCD3+.

Error bars are mean±SD. ns=not significant, *p<0.05, **p<0.01, unpaired two-tailed t-test.

SUPPLEMENTARY TABLES

Table S1 - Panther pathway analysis of upregulated genes in a given cell population. Related to results presented in Figures 1 and 2.

	Term	Enrichment Ratio	False discovery rate (FDR)
**SC-α in vivo SCRNA-seq**	JAK/STAT signaling pathway	5.419432	7.69E-04
	FAS signaling pathway	2.950094	0.026945
	Apoptosis signaling pathway	2.916708	1.20E-06
	T cell activation	2.845202	2.69E-04
	Toll receptor signaling pathway	2.438744	0.028295
	Inflammation mediated by chemokine and cytokine signaling pathway	1.879865	0.00133
**SC-β in vivo SCRNA-seq**	JAK/STAT signaling pathway	15.10667	0.09678
	Oxidative stress response	6.568116	0.156495
	T cell activation	4.028444	0.594957
	PDGF signaling pathway	3.021333	0.623899
	Ras Pathway	3.237143	1
	Toll receptor signaling pathway	3.021333	1
**SC-EC in vivo SCRNA-seq**	Interferon alpha/beta signaling	14.13884	0
	Antigen Presentation: Folding, assembly and peptide loading of class I MHC	13.00773	3.19E-08
	Interferon gamma signaling	11.2468	0.00E+00
	Cytokine Signaling in Immune system	4.468829	0
	Adaptive Immune System	3.089258	0.00E+00
	Immune System	2.916332	0.00E+00
**SC-α in vitro SCRNA-seq**	T cell activation	6.972308	0.501353
	Apoptosis signaling pathway	4.84188	0.599738
	Interleukin signaling pathway	4.469428	1
	JAK/STAT signaling pathway	11.62051	1
	Interferon-gamma signaling pathway	6.45584	1
	Toll receptor signaling pathway	3.486154	1
**SC-β in vitro SCRNA-seq**	JAK/STAT signaling pathway	19.70435	0.043412
	Oxidative stress response	6.425331	0.589624
	Interferon-gamma signaling pathway	7.297907	0.85533
	T cell activation	3.94087	0.85533
	Apoptosis signaling pathway	2.736715	1
	Inflammation mediated by chemokine and cytokine signaling pathway	1.970435	1
**CD4 T cells in vitro SCRNA-seq**	T cell activation	4.028444	0.071289
	Inflammation mediated by chemokine and cytokine signaling pathway	2.454833	0.08497
	Cytoskeletal regulation by Rho GTPase	3.723474	0.08497
	PDGF signaling pathway	2.7192	0.134924
	JAK/STAT signaling pathway	7.553333	0.144705
	Integrin signalling pathway	2.2751	0.153289

**CD8 T cells in vitro SCRNA-seq**	Inflammation mediated by chemokine and cytokine signaling pathway	4.532	3.80E-04
	Apoptosis signaling pathway	4.196296	0.134094
	Integrin signalling pathway	3.185141	0.180917
	T cell activation	4.028444	0.446218
	Axon guidance mediated by Slit/Robo	8.392593	0.508594
	Toll receptor signaling pathway	4.532	0.508594
**NK cells in vitro SCRNA-seq**	Apoptosis signaling pathway	3.38411	4.47E-02
	T cell activation	3.898495	0.04465
	Inflammation mediated by chemokine and cytokine signaling pathway	2.375645	0.063865
	JAK/STAT signaling pathway	7.309677	0.158653
	Integrin signalling pathway	2.20171	0.183223
	FAS signaling pathway	3.536941	0.575141

Table S2 - Reactome pathway analysis of upregulated genes in a given cell population. Related to results presented in Figures 1 and 2.

	**Term**	**Enrichment Ratio**	**FDR**
**SC-α in vivo SCRNA-seq**	Interferon alpha/beta signaling	7.109533	0.00E+00
	Interferon gamma signaling	6.246232	0.00E+00
	Interferon Signaling	4.33996	0
	Programmed Cell Death	3.341008	6.27E-10
	Apoptosis	3.151512	2.09E-08
	Cytokine Signaling in Immune system	2.892824	0
**SC-β in vivo SCRNA-seq**	Interferon alpha/beta signaling	26.14641	0
	Interferon gamma signaling	19.60981	0
	Antigen processing-Cross presentation	14.57861	2.75E-12
	Interferon Signaling	13.73682	0
	Cytokine Signaling in Immune system	5.637821	0
	Immune System	3.252263	0
**SC-EC in vivo SCRNA-seq**	Interferon alpha/beta signaling	14.13884	0
	Antigen Presentation: Folding, assembly and peptide loading of class I MHC	13.00773	3.19E-08
	Interferon gamma signaling	11.2468	0.00E+00
	Cytokine Signaling in Immune system	4.468829	0
	Adaptive Immune System	3.089258	0.00E+00
	Immune System	2.916332	0.00E+00
**SC-α in vitro SCRNA-seq**	Cytokine Signaling in Immune system	10.06695	0
	Antigen processing-Cross presentation	24.9858	0
	Interferon gamma signaling	28.67935	0
	Interferon alpha/beta signaling	45.40897	0
	Antigen Presentation: Folding, assembly and peptide loading of class I MHC	52.77	2.51E-10
	Class I MHC mediated antigen processing & presentation	8.000842	7.03E-10
**SC-β in vitro**	Cytokine Signaling in Immune system	7.912271	0
	Interferon gamma signaling	20.52838	0

**SCRNA-seq**	Interferon alpha/beta signaling	37.03158	0
	Adaptive Immune System	3.673768	2.10E-06
	Antigen processing-Cross presentation	11.22169	4.29E-06
	Signaling by Interleukins	4.328366	2.35E-05
**CD4 T cells in vitro SCRNA-seq**	Axon guidance	4.14862	0
	rRNA processing in the nucleus and cytosol	7.731868	0
	Major pathway of rRNA processing in the nucleolus and cytosol	8.149807	0
	Regulation of expression of SLITs and ROBOs	9.385884	0
	Eukaryotic Translation Initiation	13.07857	0
	Formation of a pool of free 40S subunits	14.92786	0
**CD8 T cells in vitro SCRNA-seq**	Innate Immune System	3.233159	2.43E-06
	Signaling by Interleukins	3.684541	0.004806
	Cytokine Signaling in Immune system	2.804107	0.031589
	Immunoregulatory interactions between a Lymphoid and a non-Lymphoid cell	6.018084	0.043057
	CD28 co-stimulation	13.75562	0.043057
	Cell surface interactions at the vascular wall	5.798446	0.043057
**NK cells in vitro SCRNA-seq**	Immunoregulatory interactions between a Lymphoid and a non-Lymphoid cell	7.902484	3.34E-08
	Cytokine Signaling in Immune system	3.210722	1.60E-07
	Innate Immune System	2.505698	3.71E-06
	Signaling by Interleukins	3.453186	9.57E-06
	Adaptive Immune System	2.759598	1.28E-05
	Interferon Signaling	3.737693	0.015391

Table S3 – Gene Ontology (GO) terms of biological processes of upregulated genes in a given cell population. Related to results presented in Figures 1 and 2.

	**Term**	**Enrichment Ratio**	**FDR**
**SC-α in vivo SCRNA-seq**	negative regulation of immune system process	3.550315	0
	activation of immune response	3.260719	0
	immune effector process	3.242858	0
	leukocyte activation	3.059432	0
	regulation of immune system process	2.865024	0
	leukocyte mediated immunity	2.802487	0
**SC-β in vivo SCRNA-seq**	response to type I interferon	23.08666	0
	cellular response to interferon-gamma	13.937	0
	cytokine-mediated signaling pathway	7.124298	0
	cellular response to cytokine stimulus	6.297969	0
	defense response	4.812677	0
	immune response	4.639788	0
**SC-EC in vivo SCRNA-seq**	negative regulation of immune system process	4.736849	0
	activation of immune response	4.37812	0
	immune effector process	4.06874	0
	leukocyte activation	3.892765	0
	regulation of immune system process	3.769293	0

	leukocyte mediated immunity	3.251988	0
**SC-α in vitro SCRNA-seq**	immune response	6.221897	0
	defense response	7.70163	0
	response to cytokine	9.497585	0
	innate immune response	10.52735	0
	defense response to other organism	12.09548	0
	response to interferon-gamma	22.02441	0
**SC-β in vitro SCRNA-seq**	immune response	5.299158	0
	defense response	6.792044	0
	immune effector process	5.322702	0
	response to cytokine	7.575455	0
	innate immune response	9.393048	0
	response to virus	15.0535	0
**CD4 T cells in vitro SCRNA-seq**	immune response	2.924152	0
	regulation of immune system process	3.126378	0
	cell activation	3.026399	0
	leukocyte activation	3.222791	0
	establishment of protein localization to organelle	4.911323	0
	protein targeting	5.584208	0
**CD8 T cells in vitro SCRNA-seq**	immune response	3.501908	2.02E-12
	leukocyte activation	4.200101	7.00E-11
	cell motility	3.541587	6.17E-10
	response to cytokine	4.154282	6.21E-10
	cell migration	3.635175	1.93E-09
	regulation of immune response	4.43575	2.74E-09
**NK cells in vitro SCRNA-seq**	cell activation	3.159824	4.04E-13
	regulation of immune response	3.803824	4.04E-13
	response to cytokine	3.39481	1.51E-12
	cellular response to cytokine stimulus	3.474709	3.75E-12
	cytokine-mediated signaling pathway	3.923604	1.08E-10
	lymphocyte activation	3.960806	6.91E-10

Supplemental Experimental Procedures

- **KEY RESOURCES TABLE**
- **CONTACT FOR REAGENT AND RESOURCE SHARING**
- **EXPERIMENTAL MODEL AND SUBJECT DETAILS**
- **METHOD DETAILS**
  - **hESC cell culture and differentiation**
  - **Cell transplantation**
  - **Secretion assays**
  - **In vivo bioluminescence imaging**
  - **Flow cytometry**
  - **Immunofluorescence Microscopy**

- **Magnetic enrichment using CD49a**
- **Human primary immune cell isolation and co-culture assays**
- **Tissue/cell preparation and library preperation for single cell RNA sequencing**
- **In vivo single cell RNA sequencing analysis**
- **In vitro single cell RNA sequencing analysis**
- **Lentivirus Preparation and Transduction**
- **Whole genome CRISPR screen in vivo and analysis**
- **Generation of hESC knockout lines**

- **QUANTIFICATION AND STATISTICAL ANALYSIS**
- **GRAPHIC ILLUSTRATIONS**
- **DATA AVAILABILITY**

KEY RESOURCES TABLE

REAGENT or RESOURCE	SOURCE	IDENTIFIER
**Antibodies**		
Rat anti-C-peptide	Developmental Studies Hybridoma Bank (DHSB)	GN-ID4, RRID: AB_2255626
Mouse anti-NKX6.1	DHSB	F55A12, RRID: AB_532379
Goat anti-NANOG	R&D Systems	AF1997
Goat anti-SOCS1	Life Technologies	Cat#PA517938
Mouse anti-glucagon Alexa 647	Santa Cruz Biotech	Cat#SC-514592
Rabbit anti-STAT1	abcam	Cat#ab2415
Mouse anti-CD8	BD Biosciences	HIT8a, Cat#550372, RRID: AB_393643
Mouse anti- NKX6.1 PE-conjugated	BD Biosciences	Cat#563023
Mouse anti-pSTAT1 Alexa 647-conjugated	Biolegend	Cat#666410
Mouse anti-SOCS1 Alexa a88-conjugated	Santa Cruz Biotech	Cat#SC-518028
Mouse anti-IP10 PE-conjugated	Biolegend	Cat#519504
Mouse Anti-HLA-ABC Alexa 647-conjugated	Biolegend	W6/32, Cat#311414
Mouse Anti-HLA-ABC PE-conjugated	Biolegend	W6/32, Cat#311406
Mouse anti-HLA-E PE-conjugated	BD Biosciences	Cat#12995342
Mouse anti-CD119 PE-conjugated	Biolegend	GIR-94, Cat#308704
Rat anti CD16/CD32 (Fc Block)	BD Biosciences	Cat# 553141, RRID: AB_394656
Rat anti murine CD45 Alexa 647-conjugated	Biolegend	Cat#03124

Mouse anti-CD45 Alexa a88-conjugated	Biolegend	Cat#304017
Mouse anti-CD3 APC-conjugated	Biolegend	UCHT1, Cat#300412
Mouse anti-CD3 PE-conjugated	Biolegend	UCHT1, Cat#300408
Mouse anti-PD-L1 APC-conjugated	Biolegend	29E.2A3, Cat#329708
Mouse anti-CD3 PB-conjugated	Biolegend	UCHT1, Cat#300417
Mouse anti-CD8 PE-conjugated	Biolegend	T8-Leu2, Cat#344706
Mouse anti-CD4 PE/Cy7-conjugated	Biolegend	RPA-T4, Cat#300512
Mouse anti-CD69 Alexa 647-conjugated	Biolegend	FN50, Cat#310918
Mouse anti-CD25 Alexa 700-conjugated	Biolegend	M-A251, Cat#356118
Mouse anti-CD107a FITC-conjugated	ThermoFisher Scientific	H4A3, Cat# BDB555800
Mouse anti-CD56 APC-conjugated	Biolegend	Cat#362504
Mouse anti-CD49a PE-conjugated	BD Biosciences	Cat#559596
Mouse anti-Oct4 Alexa 647-conjugated	BD Pharmingen	Cat#653710
Mouse anti-Sox2 PE-conjugated	BD Pharmingen	Cat#560291
Mouse anti-SSEA4 V450-conjugated	BD Pharmingen	Cat#561156
Mouse IgG1 PB-conjugated	Biolegend	Cat#400131
Mouse IgG1 Alexa 488-conjugated	Biolegend	Cat#400132
Mouse IgG3, κ V450- conjugated	BD Biosciences	Cat#561600
Mouse IgG1 PE-conjugated	Biolegend	Cat#400114
Mouse IgG2b, κ PE-conjugated	Biolegend	Cat#400314
Mouse IgG2a, κ PE-conjugated	Biolegend	Cat#400214
Mouse IgG2a, κ Alexa 647-conjugated	Biolegend	Cat#400234
Mouse IgG2b, κ Alexa 647-conjugated	Biolegend	Cat#400330
Mouse IgG1 Alexa 647-conjugated	Biolegend	Cat#400136
Mouse IgG2a, κ APC-conjugated	BD Biosciences	Cat#17472442
Mouse IgG2b, κ APC-conjugated	Biolegend	Cat#400322
Mouse IgG2b, κ Alexa 700-conjugated	Biolegend	Cat#400334
Mouse IgG1 Alexa PE/Cy7-conjugated	Biolegend	Cat#400126
Donkey anti-mouse Alexa 647	Life Technologies	Cat#A31571
Donkey anti-rabbit Alexa 488	Life Technologies	Cat#A21206
Donkey anti-rabbit Alexa 594	Life Technologies	Cat#A21209
Donkey anti-goat Alexa 647	Life Technologies	Cat#A21447
Donkey anti-rat 488	Life Technologies	Cat# A21208
**Virus vectors**		
lentiCRISPRv2	Broad institute	RRID: Addgene_52961
pLX_307	Broad institute	RRID: Addgene_41392

pHDM-vsvG, -tat, rev, gag/pol	Harvard Medical School DNA Resource Core	N/A
**Biological Samples**		
Human Peripheral Blood apheresis collars	Kraft Family Blood Donor Center, Brigham and Woman's Hospital	N/A
**Oligonucleotides**		
Non targeting gRNA TTTACGATCTAGCGGCGTAG	This paper	N/A
B2M gRNA GCTACTCTCTCTTTCTGGCC	This paper	N/A
CXCL10 gRNA (for virus and HDR) GTAATCAACCTGTTAATCCA	Virus: eSpCas9-LentiCRISPR v2 by Genescript<br>HDR: IDT (Alt-R)	N/A
STAT1 gRNA (for virus) TGCTGGCACCAGAACGAATG	eSpCas9-LentiCRISPR v2 by Genescript	N/A
STAT1 gRNA#1 (for HDR) Alt-R AAAGCTGGTGAACCTGCTCC	Alt-R IDT	N/A
STAT1 gRNA#2 (for HDR) Alt-R GCAGCTTGACTCAAAATTCC	Alt-R IDT	N/A
IFNGR1 gRNA	eSpCas9-LentiCRISPR v2 by Genescript	N/A
CXCL10 Endo Forward ATCATTGGTCACCTTTTAGTGT	IDT	N/A
CXCL10 Endo Reverse ATAATACCTTCGAGTCTGCAAC	IDT	N/A
CXCL10 Edited Reverse TATAGATCTCTCGTGGGATCAT	IDT	N/A
CXCL10 gRNA cut site Forward TTCTGGATTCAGACATCTCTTC	IDT	N/A
CXCL10 gRNA cut site Reverse TTTGCTAAGTCAACTGTAATGC	IDT	N/A
STAT1 Endo Forward AAGATTCACTTGTGTCTGCTCT	IDT	N/A
STAT1 Endo Reverse TATATTGATCATCCAGCTGTGA	IDT	N/A

STAT1 Edited Forward AGATAAATGCCTGCTCTTTACT	IDT	N/A
STAT1 gRNA cut site Forward GGTCAGATGGTGGTGTAAGTAC	IDT	N/A
STAT1 gRNA cut site Reverse CCCTTCACTTTCTATGTCAAAT	IDT	N/A
**Chemicals, Peptides, and Recombinant Proteins**		
Activin A	R&D Systems	Cat#338-AC
Rock Inhibitor Y-27632	DNSK	Cat#DNSK-KI15-02
Chir99021	Stemgent	Cat#04-0004-10
KGF	Peprotech	Cat#100-19
Retinoic acid	Sigma-Aldrich	Cat# R2625
LDN193189	Sigma-Aldrich	Cat#SML0559
Sant1	Sigma-Aldrich	Cat#S4572
PBDU	EMD Millipore	Cat#524390
XXI	EMD Millipore	Cat#565790
Alk5i II	Axxora	Cat#ALX-270-445
T3	EMD Millipore	Cat#642511
Betacellulin	ThermoFisher Scientific	Cat# 565790
Human IFN gamma	R&D Systems	Cat#285IF
Human IL-2	Peprotech	Cat#200-02
Thapsigargin	Sigma-Aldrich	Cat#T9033
**Critical Commercial Assays**		
Chromium Next GEM Single Cell 3’ Kit v3.1, 16 rxns	10X Genomics	PN-1000268
Chromium Next GEM Chip G Single Cell Kit, 48 rxns	10X Genomics	PN-1000120
Dual Index Kit TT Set A, 96 rxns	10X Genomics	PN-1000215
Kapa qPCR Library Quantification Kit, Complete Universal Kit	Roche Sequencing Solutions	Cat#07960140001
High Sensitivity D5000 Tape	Agilent Technologies	5067-5592
High Sensitivity D5000 Reagents |	Agilent Technologies	5067-5593
**Experimental Models: Cell Lines**		
Human ESC line Hues8	HSCI	HVRDe008-A
Human ESC line Hues8 GAPDH-luciferase (GL)	HSCI	HVRDe008-A-1
Lenti-X™ 293T Cell Line	Takara Bio	Cat#632180

Software and Algorithms		
GSEA 4.1.0	UC San Diego and Broad Institute	N/A
FlowJo v10.8.1	BD (Becton, Dickinson and Company)	N/A
GraphPad Prism9	GraphPad Software	N/A
R v4.0.3	R	N/A
Seurat suite v4.0.6	Hao et al., 2021	N/A
Scanpy v1.8.1	Wolf et al., 2018	N/A

CONTACT FOR REAGENT AND RESOURCE SHARING

Further information and requests for resources and reagents should be directed to and will be fulfilled by the Lead Contact, Douglas A. Melton (dmelton@harvard.edu).

EXPERIMENTAL MODEL AND SUBJECT DETAILS

All procedures were performed in accordance with the Institutional Review Board (IRB) guidelines at Harvard University under IRB and Embryonic Stem Cell Research Oversight Committee (ESCRO) Protocols E00024. All animal experiments were performed in accordance with Harvard University International Animal Care and Use Committee regulations.

METHOD DETAILS

hESC cell culture and differentiation

Human embryonic stem-cell (hESC) Hues8 maintenance and differentiation was carried out as previously described (Pagliuca et al., 2014). Induced pluripotent stem-cell lines were obtained from stocks maintained by the Melton laboratory. hESC line was maintained in cluster suspension culture format using mTeSR-1 (Stem Cell Technologies, 85850) in 500-ml spinner flasks (Corning, VWR) spinning at 70 r.p.m. in an incubator at 37 °C, 5% CO_2 and 100% humidity. Cells were passaged every 72h or 96h: induced human pluripotent stem-cell clusters were dissociated to single cells using gentle cell dissociation reagent (Stem Cell Technologies; 07174) and light mechanical disruption, counted and seeded at 0.6 M cells/ml in mTeSR-1 + 10 μM Y27632 (ROCK inhibitor). Cell lines were authenticated by DNA fingerprinting, karyotyping (Cell Line Genetics) and all lines tested negative on routine mycoplasma contamination verifications. Differentiation flasks were started 72 h after passaging, by replacing mTeSR-1 medium with the appropriate differentiation medium including growth factors and small molecule supplements as previously described (Veres et al., 2019):

SC-β cells protocol

Stage 1: 24 hours in S1 medium supplemented with Activin A (100ng/ml), CHIR99021 (1.4µg/ml) and Rock Inhibitor (10µM), followed by 48 hours Activin A (100ng/ml) only.

Stage 2: 72 hours in S2 medium supplemented with KGF (50ng/ml) and Rock Inhibitor (10µM).

Stage 3: 48 hours in S3 medium supplemented with KGF (50ng/ml), LDN193189 (200nM), Sant1 (0.25µM), retinoic acid (2µM), PBDU (500nM) and Rock Inhibitor (10µM).

Stage 4: 5 days in S3 medium supplemented with KGF (50ng/ml), Sant1 (0.25µM), retinoic acid (0.1µM) and Rock Inhibitor (10µM).

Stage 5: 7 days in BE5 medium supplemented with Betacellulin (20ng/ml), XXI (1µM), Alk5i-II (10 µM) and T3 (1µM). Sant1 (0.25µM) was added in the first three days, and retinoic acid was added at 0.1µM in the first three days, then at 0.025µM.

Stage 6: 14-21 days in S3 medium, changed every 48 hours.

During feeds, the differentiating clusters were allowed to gravity-settle for 5–10 min, medium was aspirated, and 300 ml of pre-warmed medium was added. All experiments involving human cells were approved by the Harvard University IRB and ESCRO committees.

Cell transplantation

Pre-surgery animals were housed in groups within sterile cages with unrestricted access to food and water. Ambient temperature was maintained between 18 and 25 °C, humidity 30−70% with 12 h light/dark cycles. All animal research was conducted under Harvard IACUC approval. Transplantation of cell clusters was performed as previously described (Pagliuca et al., 2014). Briefly, 5M cells were injected under the kidney capsule of male NSG-(Kb Db)^{null} (IA^{null}) (DKO) (Jackson Labs; 025216) >8 wk old mice. Post-surgery, mice were single housed and monitored for up to 18 weeks after transplantation. For allograft rejections assays, 50M of human primary peripheral mononuclear cells (PBMCs) were injected intraperitoneally.

Kidney grafts were harvested, processed for sequencing or stained as described below.

For *in vivo* glucose stimulated insulin secretion (GSIS) and graft function monitoring, Human insulin and C-peptide were quantified from mouse blood plasma collected from the facial vein at fasted (overnight for 16h) and 30 min post-injection of glucose at 2g/kg bodyweight.

Secretion assays

Mouse plasma was used to measure human insulin or human C-peptide by ELISA (ALPCO Diagnostics, 80-INSHUU-E10 and 80-CPTHU-E10 respectively) as described in the manufacturer's protocol. Supernatant from treated cell cultures was used to measure human CXCL10 by ELISA (BioLegend, 439904) as described in the manufacturer's protocol.

Flow cytometry (FC)

All stained cells were analyzed using the Attune NxT (Thermo Fischer) flow cytometer. Data analysis was performed with FlowJo (BD) software. For IFN induced protein detection, SC-islets were treated with 20ng/ml recombinant human (rh)IFNγ, 48hr prior to FC staining. For intracellular staining (ICS) of CXCL10, SC-islets were also treated with 2μM monensin for 6hrs (BioLegend, #420701) to block secretion.

Intracellular staining

Differentiated SC-islet clusters, sampled from suspension cultures (1–2 ml), were dissociated using TrypLE Express (Gibco, 12604013) at 37 °C, mechanically disrupted to form single cells, fixed using 1% paraformaldehyde (PFA) overnight and stored at 4 °C. For staining, fixed single cells were incubated in Perm/Wash Buffer (BD Biosciences, 554723) for 30 min at room temperature, then incubated in Perm/Wash Buffer with primary antibodies (1 h at room temperature), washed three times with Perm/Wash Buffer, incubated with secondary antibodies in Perm/Wash Buffer (1 h at room temperature), washed three times and resuspended in Perm/Wash Buffer.

Surface marker staining

PBS containing 4% Fetal Bovine Serum (FBS) was used as blocking and staining buffer. Immune cells or other dissociated single cells were washed and blocked with blocking buffer for 30 min at 4 °C, then incubated in blocking buffer with conjugated antibodies (1h at at 4 °C), washed three times with blocking buffer, fixed using 1% PFA overnight and stored at 4 °C.

Human lymphocyte staining from mouse whole blood

PBS containing 2% FBS was used as blocking and staining buffer. Cell suspensions from whole blood were washed with blocking buffer and preincubated with Mouse BD Fc Block™ (anti CD16/CD32 Ab, BD Biosciences). Cells were then incubated in blocking buffer with conjugated antibodies (1h at at 4 °C), washed with blocking buffer and fixed with BD FACS lysing solution (BD Biosciences) to lyse red blood cells. For analysis of human immune cells murine cells were identified and excluded by staining with anti-murine CD45 Ab. %CD4+ and %CD8+ were gated from hCD45+/hCD3+

Immunofluorescence Microscopy

Differentiated clusters or tissues were fixed in 4% PFA for overnight at 4 °C, transferred to 30% sucrose overnight, frozen in OCT (Tissue-Tek) and cryostat sectioned. For staining, slides were incubated in CAS block (ThermoFisher, 008120) with primary antibody overnight at 4 °C, washed three times, incubated in secondary antibody for 2 h at room temperature, washed, mounted in ProLong Diamond Antifade Mountant with DAPI, covered with coverslips and sealed with clear nail polish. Representative regions were imaged using Zeiss.Z2 with Apotome microscope.

Magnetic enrichment using CD49a

Stage 6 SC-islet clusters were dissociated using TrypLE Express for 20 min at 37°C. Cells were then quenched with S3 + 10% FBS and spun down. Remaining undissociated cell clusters were mechanically dissociated using a P1000 pipette. The dissociated single cells were resuspended in sorting buffer (PBS + 1% BSA + 2 mM EDTA) and filtered through a 37-µm mesh filter. Cells were counted and resuspended at a density of 10 million cells per 300 µL in 15 mL conical tubes. Cells were stained at room temperature for 20 min using a 1:100 dilution of anti-human CD49a PE-conjugated (BD Biosciences) antibody, covered from light and agitated every 3 minutes. Stained cells were washed twice with 15 mL of sorting buffer by spinning down (5 min, 300 g) and resuspended to their initial density of 10 million cells per 300 µl. To label with microbeads, 40 µL of anti-PE UltraPure MACS microbreads (Miltenyi 130-105-639) were added for each 10 million cells, and the cell solution was incubated for 15 min at 4°C, agitated every 5 min. The stained cells were washed twice as above and resuspended to a target density of 25–30 million cells per 500 µl. Volumes of 500 µL (containing no more than 30 million cells) were then magnetically separated on LS columns (Miltenyi 130-042-401) in a QuadroMACS separator (Miltenyi 130-090-976) using the recommended protocol. Successful PE enrichment was verified by live-cell flow cytometry on an Attune NxT (Invitrogen) flow cytometer.

Human primary immune cell isolation and co-culture assays

We obtained healthy donors' blood derived apheresis collars from Brigham and Woman's Hospital. Human PBMCs were isolated using the density gradient medium, Ficoll-Paque Plus (GE health care life sciences, 17144002) and the SepMate tubes (Stem Cell Technologies, 85450); T cells and NK cells were isolated using RosetteSep Human T Cell Isolation Kit (Stem Cell Technologies, 15061 and 15065, respectively). PBMCs or isolated cells were cultured in T-cell media: X-VIVO 10 (Lonza, 04-380Q) media supplemented with 5% Human AB Serum (Valley Biomedical, HP1022HI), 5% Fetal Bovine Serum (ThermoFisher Scientific, A3840101), 1% Penicillin/Streptomycin (ThermoFisher Scientific, 15070063), GlutaMAX (ThermoFisher Scientific, 35050061), MEM Non-Essential Amino Acids (ThermoFisher Scientific, 11140050).

For co-culture assays, SC-islets were used as target cells. SC-islet clusters were dissociated using TrypLE Express (Gibco, 12604013) at 37 °C, mechanically disrupted to form single cells, ten thousand cells were plated per well on 96-well V-shaped bottom plates and allowed to reaggregate for 48hrs in S3 media. SC-islets were then treated with antibodies (as described) and/or thapsigargin, 5uM (Sigma Aldrich, T9033) for 5 hours before the co-culture assay. Cells were washed to remove residual thapsigargin and Immune cells (PBMCs/T-cells/NK), pre-labeled with Cell Trace Violet (CTV; ThermoFischer Scientific, C34571) were added at a ratio of 5:1 (immune;target cells) in T-cell media.

T cell activation and proliferation assays

After a 48 hours co-culture, the PBMCs/T-cell cell fraction was removed from top cell suspension (SC-islet cluster settle in the bottom). A portion of the cells were taken for flow cytometry staining for CD3+ T cells and activation markers CD69 and CD25. Results are presented as Medial Fluorescent Intensity (MFI), adjusted to baseline MFI of T-cells in an unstimulated PBMC control. The other portion was seeded on 96-well low adherence round bottom plates and allowed to expand for 7-days in T-cell media containing 20 U/ml rhIL-2. Cells were taken for flow cytometry staining and analysis of CD3+ T cells gated cells, while the frequency of CTV negative cells served as a marker for proliferated cells. PBMCs/T cells activated with Dynabeads Human T-Activator CD3/CD28 beads (ThermoFisher Scientific, 111.61) for 48 hours were used as positive control for both assays.

NK cell activation/degranulation assays

During co-culture of SC-islets and NK cells, a CD107a antibody was added to bind internalizing degranulation marker CD107a. After a 48 hours co-culture, the NK cell fraction was removed from top cell suspension and taken for flow cytometry staining and analysis of CD56+CD107a+ cells.

SC-islet and SC-β apoptosis assays

After a 48 hours co-culture, the SC-islet clusters were dissociated using TrypLE Express at 37 °C, to form single cells, fixed O.N. with 1% formaldehyde (CytoFix; Fisher Scientific, BDB554655) and then stained for flow cytometry using C-peptide antibody and terminal deoxynucleotidyl transferase dUTP nick end labeling (TUNEL) for apoptosis with the In Situ Cell Death Detection Kit (Roche Diagnostics, 12156792910). Percent apoptosis was calculated relative to baseline TUNEL staining of SC-islets with no PBMCs.

Tissue/cell preparation and library preparation for single cell RNA sequencing

For graft extraction, mice were euthanized, and the transplanted kidney was removed. SC-islets graft was peeled off the kidney, was sliced into small pieces and digested in 2mg/mL collagenase D (Sigma, 11088858001) in RPMI (GIBCO, 11875-085) for 45 min in 37 °C. Additional breaking was used by pipetting and by filtering through a 40μm cell strainer. Cells were then centrifuged and resuspended in 0.5% BSA in PBS and a magnetic mouse depletion kit (Miltenyi, 130-104-694) was used to remove residual mouse cells.

For co-cultured experiment, cells were collected from 96 wells after 48hrs of co-cultured and SC-islets dissociated using TrypLE Express at 37 °C, to form single cells and quenched with S3 media.

Harvested cells from both sources were centrifuged, resuspended in 0.04% BSA in PBS, counted (LUNA-FX7 Automated Cell Counter), adjusted to 1000 cells/μl and sent to the Harvard University Bauer Sequencing Core for 10X Chromium Single Cell 3’ Library preparation and sequencing.

All samples were loaded into Chip G per the user guide from 10x Genomics, no alterations were made at any step of the protocol (Part No. CG000315). GEMs were formed targeting 10,000 cells and reverse transcription completed immediately after. The cDNA was cleaned from the GEM reagents, amplified for a total of 11 cycles and verified via TapeStation (Agilent Technologies). Amplified cDNA was diluted and ran on the 4200 TapeStation instrument using High Sensitivity D5000 tape and reagents (Part No. 5067-5592 & 5067-5593). The amplified cDNA was fragmented, end repaired, and A-tailed followed by adaptor ligation, and PCR amplification for a total of 12 cycles with each sample receiving a unique set of dual indices (Part No. 1000215). Final libraries were diluted and ran using the High Sensitivity D5000 tape and reagents (Part No. 5067-5592 & 5067-5593) on the 4200 TapeStation (Agilent Technologies). Libraries were quantified via Kapa qPCR using the Complete Universal Kit (Part No. 07960140001, Roche Sequencing Solutions) and the CFX96 Touch Real-Time PCR Detection System (Bio-Rad Laboratories). Libraries were sequenced on an Illumina NovaSeq instrument using the parameters outlined in the user guide (Read1: 28 bp, i7 index: 10 bp, i5 index: 10 bp, Read2: 90 bp).

***In vivo* single cell RNA sequencing analysis**

Raw sequencing files were processed using Cell ranger 5.0.0 (10X Genomics). Illumina basecall files were converted to fastq format. Samples were aligned to the GRCh38 genome using STAR aligner (Dobin et al., 2013). Graft samples were also aligned to a GRCh38/mm10 hybrid genome to obtain the human/mouse percentage for each cell. Cells with less than a 50% of aligned reads mapping to the human reference, were discarded.

Processed scRNASeq data was analyzed in R version 4.0.3 using the Seurat suite version 4.0.6 (Hao et al., 2021). Count matrices were loaded into a Seurat object filtering out genes detected in less than 3 cells and cells with less than 200 genes. Quality control filtering was adjusted for each sample as indicated in **Supplementary File 2**. A total of 35,647 cells from 14 samples passed these quality control steps (**Supplementary File 2)**. Data normalization and scaling were performed using Seurat's SCTransform function adding the mitochondrial percentage as an additional regressed variable.

Samples were integrated in two ways. Grafts injected with PBMCs (hPi) and graft samples without injection (control) were integrated to assess the graft component. hPi samples and control PBMC samples were integrated to explore differences in the immune component. In both cases, samples were integrated following the default integration guidelines for SCT transformed datasets from https://satijalab.org/seurat/articles/integration_introduction.html, with 3000 variable features.

To explore transcriptional heterogeneity and to perform initial cell clustering, principal component analysis and nonlinear dimensional reduction using Uniform Manifold Approximation and Projection (UMAP) (McInnes et al., 2018) were applied using 40

dimensions and resolution 1.0 for the integrated hPi and control samples, and 30 dimensions and 0.8 resolution for the integrated hPi and PBMCs. Cell types were assigned to clusters using known marker genes.

Differential expression at the gene level between sample types was performed with DESeq2 1.30.1 (Love et al., 2014) (https://bioconductor.org/packages/DESeq2/) using a pseudobulk approach where counts are aggregated for each cluster at the sample level.

Seurat's plotting functions were used to obtain violin plots. Tidyverse packages (Hadley Wickham (2017). tidyverse: Easily Install and Load the 'Tidyverse'. R package version 1.2.1. https://CRAN.R-project.org/package=tidyverse) were used for data processing.

The distribution of the SC alpha, SC_beta and SC_EC clusters were further analyzed. By selecting the cells from these clusters and reclustering them. Principal component analysis and nonlinear dimensional reduction using UMAP (McInnes et al., 2018) were applied using 10 dimensions and resolution 0.1.

In vitro single cell RNA sequencing analysis

Raw sequencing files were processed using Cell ranger 5.0.0 (10X Genomics). Illumina basecall files were converted to fastq format. Samples were aligned to the GRCh38 genome using STAR aligner (Dobin et al., 2013).

Processed scRNASeq data was analyzed in R version 4.0.3 using the Seurat suite version 4.0.6 (Hao et al., 2021). Count matrices were loaded into a Seurat object filtering out genes detected in less than 3 cells and cells with less than 200 genes. During quality additional filtering was used. Cells with less than 1000 genes, less than 3000 UMIs or with a higher mitochondrial percentage than 15%, were discarded. A total of 42,922 cells from 8 samples passed these quality control steps . Data normalization and scaling were performed using Seurat's SCTransform function adding the mitochondrial percentage as an additional regressed variable. All samples were integrated using Harmony (Korsunsky et al., 2019) regressing the time point variable.

To explore transcriptional heterogeneity and perform initial cell clustering, principal component analysis and nonlinear dimensional reduction using UMAP (McInnes et al., 2018) were applied using 20 dimensions and resolution 0.3. Cell types were assigned to clusters using known marker genes. An evaluation of the UMAP plots showed that 61 cells had been overcorrected (i.e: endocrine cells in the PBMCs only samples or vice versa) during the integration and were removed from the analysis.

For differential expression and dot plot generation, data was also processed using Scanpy (version 1.8.1) (Wolf et al., 2018) to annotate the cell types. Cell ranger output was filtered to retain cells with no more than 15% MT transcripts and 4000 highly variable genes were identified using the highly_variable_genes function with the Seurat v3 option.

Cell types were assigned to clusters using known marker genes. Results of identified cell types were then visualized using dotplot plotting functions.

Using the annotations, differential expression was measured using fold change between treatment conditions and the Mann-Whitney hypothesis test p-values after correction for multiple comparisons using the FDR procedure implemented in the multipletests function from the statsmodels library.

Lentivirus Preparation and Transduction

Lentiviral particles were produced by transfecting 293T cells (Takara Bio) with the packaging vectors pHDM-vsvg, pHDM-tat, pHDM-rev, and pHDM-gag/pol along with lentiviral backbone vectors using the TransIT-293 transfection reagent (Mirus). Lentiviral particles were collected 48 h and 72 h post transfection and concentrated using the PEG-IT virus precipitation reagent (Fisher Scientific, Waltham, MA, USA) overnight at 4 °C followed by centrifugation at 1500 g for 30 min at 4 °C and stored at −80 °C.

The lentiviral vector lentiCRISPRv2 [a gift from Feng Zhang (Addgene plasmid # 52961 ; http://n2t.net/addgene:52961; RRID:Addgene_52961 (Sanjana et al., 2014)] was used to clone guide RNAs (gRNA sequences described in Key resource table; custom clone service from GensScript). For overexpression, lentiviral vectors containing open reading frame (ORF) sequences of eGFP, *CD274*, *CXCL10* and *SOCS1*, cloned into pLX_307, were obtained from the Broad institute inventory.

For transduction, cell clusters collected from spinner flask suspension cultures were dissociated in TrypLE Express (Life Technologies) for 7 min, followed by mechanical dissociation and centrifugation at 300 g for 5 min at room temperature (RT). Cell pellets were resuspended at a density of 2.5 million cells/mL in the stage-matched medium with polybrene reagent (Santa Cruz) at 10 µg/mL. Single-cell suspensions were combined with concentrated lentiviral particles and allowed to reaggregate in spinner flasks, in a humid 37 °C incubator and 5% CO_2.

Whole genome CRISPR screen in vivo and analysis

Brunello pooled library pooled plasmid DNA in a 1 vector system (lentiCRISPRv2 backbone; Addgene # 73179) was obtained from the Broad institute Genetic Perturbation Platform (GPP), to generate pooled lentivirus. Lentivirus and SC-islet transduction was as described above. To determine a titer that will lead to a multiplicity of infection (MOI) that is less than 1, SC-islets were seeded in 6 well plates and treated with different virus volumes per cell number. After 2-3 days transduced SC-islets were treated with puromycin (9µg/ml) and cell counts were taken after 4 days to evaluate cell death ratios compared to a control well with no selection.

Library transduced (LT) SC-islets were transplanted under the kidney capsule of NSG-MHC^{null} nice and PBMCs were injected at week 4 after transplantation. Full experiment layout is described in the results section and in Figure 1**.** Retrieved graft tissue were homogenized (Polytron PT 1200E, KINEMATICA) and a Quick-DNA™ Midiprep Plus Kit

(Zymo Research, D4075) was used to extract genomic DNA (gDNA). gDNA was submitted to GPP for PCR amplification of the integrated construct containing a barcode sequence, and Illumina sequencing to determine the abundance of each gRNA in each sample. Sequencing resulted in 132,183,231 matching reads which consists of a 82% of total reads. PoolQ v3 software was used to deconvolute sequencing files and quantitate gRNA barcodes counts in each sample (https://portals.broadinstitute.org/gpp/public/software/poolq).

To identify genes that may influence graft depletion, we estimate the gene by environment interactions of KO allele targets and PBMC graft environments (hPi), which can be interpreted as a difference in PBMC depletion between the KO and WT alleles. A separate model is fit for each of the target genes using observed sequenced read counts as the outcome and all available data across mouse and guide replicates. Read counts are modeled as negative binomial, with additive random effects for targeting guide and mouse, and fixed effects representing the graft allele and condition. The full model is given by:

log(counts) ~ 1+KO+PBMC+KO*PBMC+(1|Mouse)+(1|Guide)

Significance of the KO*PBMC interaction is evaluated using a likelihood ratio test comparing with a reduced model that only includes the additive effects. All of the models are fit using the glmer.nb function from the lme4 R package. To correct for multiple comparisons, p-values are adjusted using the Benjamini-Hochberg FDR procedure, implemented in the R function p.adjust with option 'fdr'. A filter was applied to remove any gene hits that were not expressed in at least 10% of either SC-α or SC-β cells in at least one experimental condition across *in vivo* and *in vitro* scRNA-seq experiments (Figures 1 and 2). To visualize the results from a subset of selected CRISPR targets, we produced boxplots of the full model predictions for each allele and treatment combination.

Generation of hESC knockout lines

Gene modified (GM) lines were generated by homology directed repair (HDR), via nucleofection of a Cas9/sgRNA ribonucleoprotein complex (RNP) and a targeting vector. The targeting vector (OriGene) was designed to facilitate the in-frame integration of GFP or luciferase cassettes with puromycin resistance into exon 2 or exon 3 of the CXCL10 or STAT1 loci, respectively (**Figures 4A and 4B**). Culture and expansion were performed on Matrigel® (Corning) coated plates with mTeSR™ Plus media (Stem Cell Technologies). Cells were clump passaged every 72h or 96h. For nucleofection Hues8 monolayers were dissociated into single cells with Accutase (Stem Cell Technologies), and $1x10^6$ cells were nucleofected using the 4D-Nucleofector (Lonza) with 5 µg targeting plasmid and RNP (120 pmol targeting sgRNA and 104 pmol Alt-R Cas9 (IDT), according to the manufacturer's instructions. Nucleofected cells were then plated in a matrigel-coated tissue culture plate containing mTeSR™ Plus, cloneR (Stem Cell Technologies) and 7.5 µM RS-1 (Xcessbio). After 48h, puromycin (0.5µg/ml) was added and surviving colonies were transferred to 96-well plates for PCR and expansion. Genomic DNA was

extracted and purified using Quick-DNA™ (Zymo Research), and target cassette knock-in was confirmed by PCR analysis (**Figure S4A**) using Phusion® Hot Start Flex 2x master mix and primer sets (see key resource table) that amplify the wild type and targeted genomic alleles (blue and red arrows, respectively in **Figure 4A and 4B**). Several heterozygous clones were acquired from each knockout, and we selected a CXCL10-GFP (C10G) clone and a STAT1-luciferase (ST1L) clone that contained the integrated transgene in one allele along with a nonhomologous end-joining (NHEJ) mutation in the intact endogenous allele, determined by PCR and Sanger (GENEWIZ) sequencing (**Figure S4A**). Overall, C10G and ST1L contained null mutations in both allelles.

QUANTIFICATION AND STATISTICAL ANALYSIS

Statistical analysis was performed by unpaired Student's *t*-tests as indicated, using the Prism v9. All data are presented mean ± SD. $p<0.05$ was considered statistically significant. Sufficient sample size was estimated without the use of a power calculation. Data analysis was not blinded.

GRAPHIC ILLUSTRATIONS

Graphic illustrations in the manuscript were created with BioRender.com under BioRender's Academic License Terms.

DATA AVAILABILITY

scRNA-seq and pooled CRISPR screen data generated during this study are available at NCBI GEO accession number GSE200104, and composed of listed SubSeries related to specific experiments described in this paper.

REFERENCES

Dobin, A., Davis, C.A., Schlesinger, F., Drenkow, J., Zaleski, C., Jha, S., Batut, P., Chaisson, M., and Gingeras, T.R. (2013). STAR: ultrafast universal RNA-seq aligner. Bioinformatics 29, 15-21.

Hao, Y., Hao, S., Andersen-Nissen, E., Mauck, W.M., 3rd, Zheng, S., Butler, A., Lee, M.J., Wilk, A.J., Darby, C., Zager, M., et al. (2021). Integrated analysis of multimodal single-cell data. Cell 184, 3573-3587 e3529.

Korsunsky, I., Millard, N., Fan, J., Slowikowski, K., Zhang, F., Wei, K., Baglaenko, Y., Brenner, M., Loh, P.R., and Raychaudhuri, S. (2019). Fast, sensitive and accurate integration of single-cell data with Harmony. Nat Methods 16, 1289-1296.

Love, M.I., Huber, W., and Anders, S. (2014). Moderated estimation of fold change and dispersion for RNA-seq data with DESeq2. Genome Biol 15, 550.

McInnes, L., Healy, J., and Melville, J. (2018). UMAP: Uniform Manifold Approximation and Projection for Dimension Reduction, pp. arXiv:1802.03426.

Pagliuca, F.W., Millman, J.R., Gurtler, M., Segel, M., Van Dervort, A., Ryu, J.H., Peterson, Q.P., Greiner, D., and Melton, D.A. (2014). Generation of functional human pancreatic beta cells in vitro. Cell 159, 428-439.

Sanjana, N.E., Shalem, O., and Zhang, F. (2014). Improved vectors and genome-wide libraries for CRISPR screening. Nat Methods 11, 783-784.

Veres, A., Faust, A.L., Bushnell, H.L., Engquist, E.N., Kenty, J.H., Harb, G., Poh, Y.C., Sintov, E., Gurtler, M., Pagliuca, F.W., et al. (2019). Charting cellular identity during human in vitro beta-cell differentiation. Nature 569, 368-373.

Wolf, F.A., Angerer, P., and Theis, F.J. (2018). SCANPY: large-scale single-cell gene expression data analysis. Genome Biol 19, 15.
