# Supplementary material for: Whole-genome CRISPR screening identifies genetic manipulations to reduce immune rejection of stem cell-derived islets
Source: Stem Cell Reports. 2022 Sep 1;17(9):1976–90. doi: 10.1016/j.stemcr.2022.08.002 (PMC9481918; doi:10.1016/j.stemcr.2022.08.002)
Supplement: Document S2. Article plus supplemental information [file mmc4.pdf]

Stem Cell Reports

Article

OPEN ACCESS

Whole-genome CRISPR screening identifies genetic manipulations to reduce immune rejection of stem cell-derived islets

Elad Sintov,[1,*] Igor Nikolskiy,[1] Victor Barrera,[2] Jennifer Hyoje-Ryu Kenty,[1] Alexander S. Atkin,[1] Dario Gerace,[1] Shannan J. Ho Sui,[2] Kyle Boulanger,[1] and Douglas A. Melton[1,3,*]

[1]Department of Stem Cell and Regenerative Biology, Harvard Stem Cell Institute, Harvard University, Cambridge, MA, USA

[2]Bioinformatics Core, Department of Biostatistics, Harvard T.H. Chan School of Public Health, Boston, MA, USA

[3]Howard Hughes Medical Institute, Chevy Chase, MD 20815, USA

*Correspondence: sintov@fas.harvard.edu (E.S.), dmelton@harvard.edu (D.A.M.)

https://doi.org/10.1016/j.stemcr.2022.08.002

SUMMARY

Human embryonic stem cells (hESCs) provide opportunities for cell replacement therapy of insulin-dependent diabetes. Therapeutic quantities of human stem cell-derived islets (SC-islets) can be produced by directed differentiation. However, preventing allo-rejection and recurring autoimmunity, without the use of encapsulation or systemic immunosuppressants, remains a challenge. An attractive approach is to transplant SC-islets, genetically modified to reduce the impact of immune rejection. To determine the underlying forces that drive immunogenicity of SC-islets in inflammatory environments, we performed single-cell RNA sequencing (scRNA-seq) and whole-genome CRISPR screen of SC-islets under immune interaction with allogeneic peripheral blood mononuclear cells (PBMCs). Data analysis points to "alarmed" populations of SC-islets that upregulate genes in the interferon (IFN) pathway. The CRISPR screen *in vivo* confirms that targeting IFNγ-induced mediators has beneficial effects on SC-islet survival under immune attack. Manipulating the IFN response by depleting chemokine ligand 10 (CXCL10) in SC-islet grafts confers improved survival against allo-rejection compared with wild-type grafts in humanized mice. These results offer insights into the nature of immune destruction of SC-islets during allogeneic responses and provide targets for gene editing.

INTRODUCTION

Nearly 100 years ago the first type 1 diabetes (T1D) patient was treated with a "pancreatic extract," which led to the discovery of insulin (Banting et al., 1922). Since then, the basis of T1D has been shown to be an autoimmune elimination of pancreatic insulin-producing β cells. While acknowledging the impressive technological advances to manage T1D (Kovatchev, 2019), exogenous insulin administration with regular monitoring remains the primary treatment for T1D. In parallel, cadaveric islet or pancreas transplants (Shapiro et al., 2000), have proved to be effective in controlling blood glucose levels, but this treatment is limited by the lack of a consistent and readily available supply of organs/islets and the requirement for systemic immunosuppressants (Shapiro et al., 2017). The prospect of using human pluripotent stem cells (hPSCs) as an unlimited source for β cell differentiation and replacement has been advanced by developing methods to differentiate human stem cells into functional human islets (Helman and Melton, 2021; Nostro et al., 2015; Pagliuca et al., 2014; Rezania et al., 2014; Russ et al., 2015). The first reports of human clinical trials using progenitor cells (Ramzy et al., 2021) or fully differentiated and functional SC-islets (Businesswire, 2021) speak directly to this possibility.

In the light of these encouraging, albeit initial, clinical reports, a major challenge remains of protecting SC-islets from an immune response. The use of immunosuppressants can lead to complications as well as graft impairment in the long term (Lehmann et al., 2008). Encapsulation methods can provide immune protection and graft extraction advantage, but have not yet been determined to be effective (Henry et al., 2018).

Beyond encapsulation, efforts to modify the patient's immune system have been pursued to blunt or modify the immune response. This includes the use of antibodies to block co-stimulation and amplifying regulatory T cells (Herold et al., 2019; Orban et al., 2011; Raffin et al., 2020). Complementing this approach is genetic modification of the target itself, the SC-islets, to make them opaque or less immunogenic. Strategies include β-2-microglobulin (B2M) or human leukocyte antigen (HLA)-I/II depletions (Castro-Gutierrez et al., 2021; Deuse et al., 2019; Han et al., 2019; Parent et al., 2021; Wang et al., 2015) to prevent donor antigen presentation to T cells, and expression of immune check point inhibitors such as programmed death-ligand 1 (PD-L1) (Castro-Gutierrez et al., 2021; Harding et al., 2019; Yoshihara et al., 2020). Other approaches include expression of CD47 (Deuse et al., 2019, 2021) and HLA-E (Gornalusse et al., 2017) to reduce natural killer (NK) killing when HLA-A, -B, and -C are absent. Another variation is to remove HLA-A and HLA-B but retain one HLA-C allele, requiring only a small number of compatible lines to cover most of recipient populations across the world (Xu et al., 2019). All these promising strategies derive from previous knowledge and studies in other contexts; e.g., maternal-fetal immune

This is an open access article under the CC BY license (http://creativecommons.org/licenses/by/4.0/).

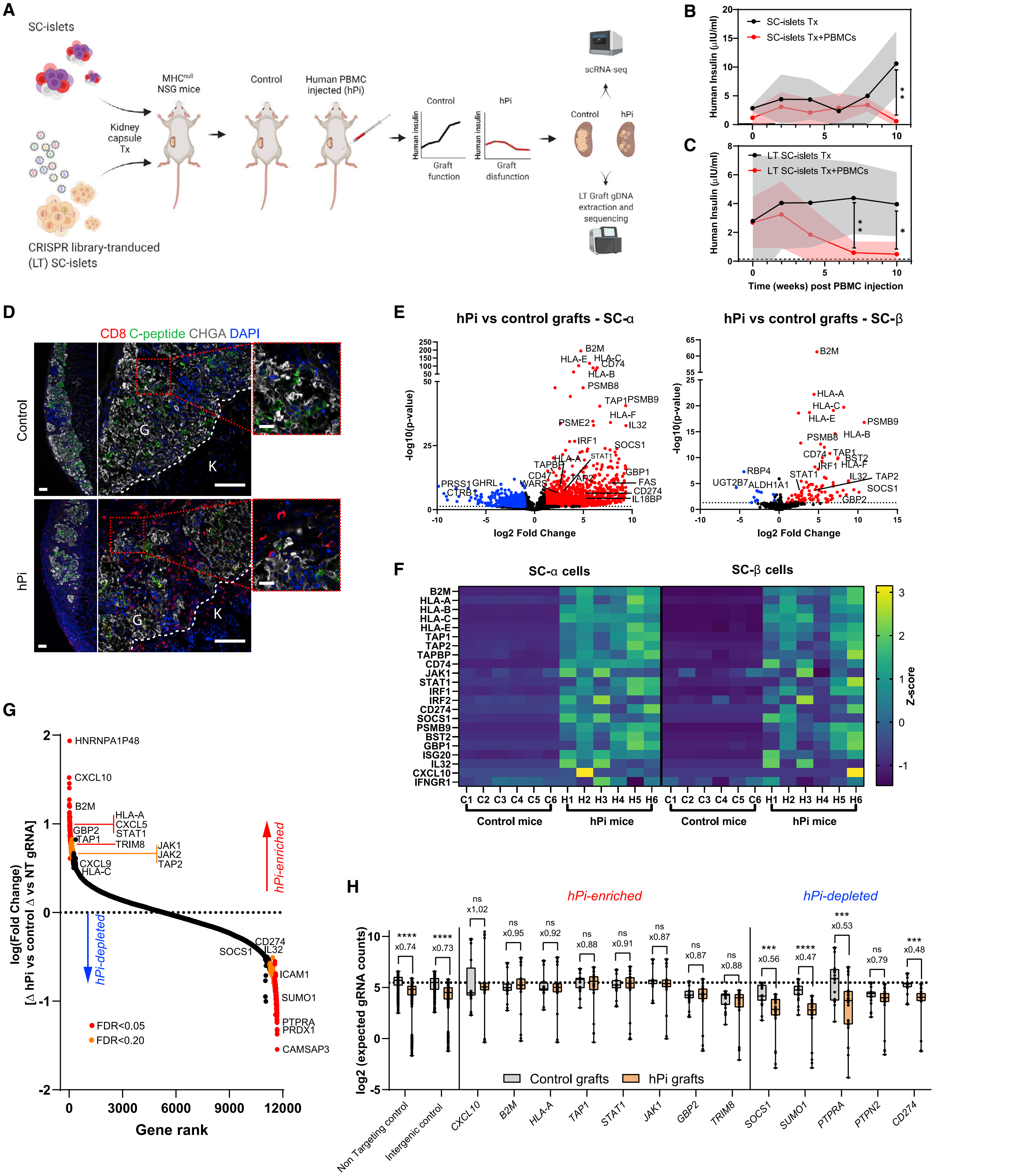

(legend on next page)

interactions and the ability of cancer cells to avoid immune elimination. Of note, there are few reports of endocrine cell-related targets for immune modulation of β cell survival and function (Cai et al., 2020; Wei et al., 2018).

Here we pursue a complementary approach by first defining the immune interaction with SC-islets, studying the interaction between the human allogeneic immune system and SC-islets with a focus on the transcriptional responses. Using single-cell RNA sequencing (scRNA-seq) and whole-genome CRISPR screening, we find that the JAK/STAT type II interferon (IFN) pathway is a leading modulator of early and late inflammatory response events both *in vitro* and *in vivo*. While manipulating the upstream and central mediators of the JAK/STAT pathway provides reduction of SC-islet immunogenicity, the findings indicate that a practical and promising approach is to target downstream components, specifically by depleting the chemokine ligand 10 (CXCL10).

RESULTS

Single-cell transcriptional analysis reveals "alarm" genes that drive immunogenicity of SC-islets

To study immune responses in the context of human allogeneic graft rejection, we chose the Hu-PBL-NSG-MHC^{null} humanized mouse (Brehm et al., 2019). NOD-scid IL-2 receptor subunit γ (IL2rg)^{null} (NSG) immunocompromised mice, which lack murine major histocompatibility complex (MHC) class I and II, were transplanted (under the kidney capsule, n = 12) with 5M (Million) SC-islets (HLA-A2 positive), followed by human PBMC injection (termed hPi-mice; 50M/mouse, n = 6) from healthy unmatched donors (HLA-A2 negative). The lack of murine MHC allowed us to monitor the graft function for prolonged durations without the risk of xenogeneic graft-versus-host disease (GVHD). Half of the SC-islet transplanted cohort (n = 6 mice) was used as the control, without PBMC injection (Figure 1A). Graft function failure was determined by human insulin detection in fasting mouse blood 30 min after glucose injection (Figure 1B). Reduction in graft size (Figures S1A) and the loss of function to a glucose challenge are attributed primarily to human T cells retained in mouse tissues (Figures S1B and S1C) for the entire experiment. CD8 cytotoxic T cells can be clearly seen infiltrating the SC-islet grafts (Figure 1D) of hPi-mice mice in week 10 and in proximity to endocrine (chromogranin A+) and SC-β cells (C-peptide+). Note that SC-islets contain several pancreatic hormone-producing cell populations, including glucagon-expressing SC-α and insulin-expressing SC-β. At 10 weeks post PBMC injections, we observed that both SC-α and SC-β numbers are reduced in hPi-mouse grafts (hPi grafts) compared with controls (Figure S1D), as expected for an allogeneic response.

Since graft elimination by PBMCs is incomplete and residual endocrine cells remain in the hPi-mice grafts, we were able to retrieve the SC-islet grafts for single-cell RNA sequencing (scRNA-seq) analysis (Augsornworawat et al., 2020). These samples were used for 10x Genomics mRNA expression library preparation and Illumina sequencing. Datasets were integrated from multiple graft and cell samples (see section "experimental procedures"). As seen in Uniform Manifold Approximation and Projection (UMAP) plots (Figures S1G and S1H), grafted endocrine cells (SC-Endo) from control and hPi-mice maintain their cell identity based on gene markers for SC-α (*INS−GCG+*),

Figure 1. Single-cell transcriptional profile and whole-genome CRISPR screen of SC-islet grafts in an *in vivo* humanized model
(A) SC-islets or CRISPR library transduced (LT) SC-islets were transplanted in MHC^{null} NSG mice. Half of each mice cohort was injected with human PBMCs, and human insulin was monitored until graft failure was observed. Grafted cells were then extracted (week 10 post PBMCs) and analyzed by scRNA-seq for gene expression, or by gDNA sequencing for gRNA abundance.
(B and C) SC-islet graft failure was assayed in fasted mouse blood by human insulin detection over time, 30 min post glucose.
(B) n = 6–8 per group of SC-islet transplanted mice.
(C) n = 6 per group of LT SC-islet transplanted mice.
(D) Immunofluorescence (IF) staining of kidney SC-islet grafts sections at week 10 after PBMC injection. Bars represent 100 μm in left (×5) and center (×20) and 20 μm in magnified view (right). Kidney (K) and graft (G) margins are outlined. CHGA, chromogranin A.
(E and F) scRNA-seq analysis of SC-islet grafts.
(E) Volcano plot of differential expressed genes in SC-β and SC-α in hPi versus control grafts.
(F) Differential expression of selected genes in different populations, presented as a heatmap. Each row specifies a *Z* score of the specified gene in all graft samples, in the indicated endocrine population.
(G) Analysis of enriched and depleted gene KOs. Rank is plotted against fold changes (hPi versus control) of gRNA counts (×4 integrated per gene) relative to integrated non-targeting (NT) gRNA counts (×941). Significant genes are color coded based on false discovery rate (FDR) as indicated.
(H) Boxplot presenting individual gRNAs counts (full model predictions) from mice replicates (n = 6 per condition times n = 4 targeting gRNAs, n = 85 for NT gRNAs, or n = 50 for intergenic gRNAs) with genes of interest with positive and negative enrichment in screen. Box lines represent median values. Dashed line represents mean of NT gRNA counts in control mice. Error bars or shaded areas are mean ± SD; ns, not significant; *p < 0.05; **p < 0.01; ***p < 0.001; ****p < 0.0001, unpaired two-tailed t test.

SC-β (*INS*+*GCG*−), and SC-enterochromaffin cells (SC-EC; *TPH1*+). hPi grafts had fewer endocrine cells (Figure S1I) compared with controls (~50% reduction), consistent with flow cytometry staining (Figure S1D).

Single-cell technology allows a focus on specific cells populations within heterogeneous SC-islets (Figures S1G–S1I). SC-α, SC-β, and SC-EC exhibited similar patterns of upregulated genes in PBMC infiltrated grafts (Figures 1E, S1J, and Data S1). This suggests that the response in this model system is not specific to a cell population within SC-islets and all transplanted cells are immunogenic.

Among the most upregulated genes are transcripts involved in antigen presentation (*B2M*; *HLA-A*, *-B*, *-C*, *-F*; *TAP1/2*; *CD74*; *PSMB9*), inflammatory pathway mediators (*STAT1*, *JAK1/2*, *IRF1/2*) and pro-inflammatory cytokines, including *IL32*. These genes induce T cell activation and inflammation. In addition, genes that are inhibitory to the immune system are upregulated; e.g. *HLA-E*, *SOCS1*, *CD274* (PD-L1), and *WARS*. Upregulation of these genes suggests an induction of IFN type I (IFNα/β) and II (IFNγ) pathways, through JAK/STAT signaling (Platanias, 2005) (Figures 1E, 1F, S1J, and S1K). A key IFN type II upstream component, the IFNγ receptor gene *IFNGR1*, does not appear to change in hPi-mouse grafts compared with controls (Figure 1F). Pathway analyses confirms the SC-islet response as IFN-driven, one that alarms the immune system through antigen presentation and that can lead to apoptosis of target cells (Tables S1–S3).

Whole-genome CRISPR screen confirms the role of IFN response genes that set the fate for SC-islet survival

Transcriptional responses of SC-islets during the immune interaction described above provide clues to genes that could be manipulated to dampen immune recognition. However, changes in expression per se might represent a pro- or anti-stimulatory response or no effect. To explore this issue, a whole-genome screen using a CRISPR lentivirus library (Doench et al., 2016) was performed.

The Brunello CRISPR library consists of a pool of 76,441 human targeting guide RNAs (gRNAs) and 1,000 control gRNAs (non-targeting [NT] or intergenic) in a lentiviral vector that expresses Cas9. The pooled library targets 19,114 human genes, most of them by four gRNAs per gene. To avoid multiple different gRNAs in cells and a nonspecific effect on the screen results (Doench, 2018), a low infection lentivirus titer (MOI < 1) was used. Library transduced cells (LT SC-islets) were allowed at least 10 days for CRISPR editing, before transplantation to the NSG-MHC^{null} mouse model, where PBMCs were injected to half of the cohort (hPi-mice, n = 6; control mice, n = 6) (Figure 1A). hPi-mice retained levels of circulating T cells throughout the experiment (Figure S1E). Graft function and subsequent failure due to human PBMC injection was assessed (Figures 1C and S1F). When hPi graft failure was confirmed, 10 weeks after PBMC injection (Figure 1C), both control and hPi grafts were recovered from kidney sites, genomic DNA (gDNA) was extracted, and gRNA regions were amplified by PCR for Illumina sequencing.

The response to PBMCs (graft infiltration) was assessed by gRNA counts from hPi LT SC-islet grafts compared with control LT SC-islet grafts, in relation to NT control gRNA counts in the two environments (see section "experimental procedures"). Essential/housekeeping genes are not evaluated because their gRNA transduced cells will have been eliminated shortly after lentiviral infection. This analysis identifies genes that increase or decrease the chance of transplanted SC-islets survival following PBMC injection (Figure 1G). Approximately 12,000 genes that are expressed in SC-islets (by scRNA-seq datasets) were ranked based on enrichment/depletion following PBMC injection. Results show reduction in total and control (NT or intergenic) gRNA reads in all hPi grafts compared with control, confirming cell elimination and graft rejection (Figures 1H left and S1M). Knockout (KO) perturbations that increase survival are positively enriched in hPi (positive values in Figure 1G) and eliminate the difference in gRNA counts between conditions (Figure 1H center). KO perturbations that decrease survival are depleted in hPi (negative values in Figure 1G) and intensify the difference in gRNA counts between conditions (Figure 1H right). We interpret hPi-enriched gene KOs as pro-survival (tolerizing) under immune attack, whereas the opposite occurs with hPi-depleted genes.

Consistent with expressed transcripts (Figures 1E and 1F), the results point to JAK/STAT signaling for antigen processing/presentation and chemokine secretion. Most prominent were the enrichments of *B2M*, *HLA-A*, *TAP1/2*, *STAT1*, *JAK1/2*, and *CXCL10* gRNAs in LT SC-islet hPi grafts (Figure 1G). KOs of these genes contribute to survival in hPi (Figure 1H).

The observed protective effect of HLA-I KOs is consistent with previous reports (Castro-Gutierrez et al., 2021; Deuse et al., 2019; Han et al., 2019; Parent et al., 2021; Wang et al., 2015). *TAP1* and *TAP2* gRNA enrichments in hPi suggest that immune protection could also be gained by disrupting transport of cytosolic peptides to HLA class I molecules (Scholz and Tampe, 2005).

Interestingly, one of the top hPi-enriched gene perturbations in this screen was for CXCL10 (IP10), an IFN-induced chemokine. Chemokine signaling plays an important role in immune cell recruitment to an inflamed tissue. Other chemokine gRNAs that are hPi-enriched include *CXCL5* and *CXCL9*. *CXCL9* is also an IFN-stimulated gene (ISG) that binds the CXCR3 receptor. *CXCL5* is known to have chemotactic and activating functions on neutrophils (Chang et al., 1994).

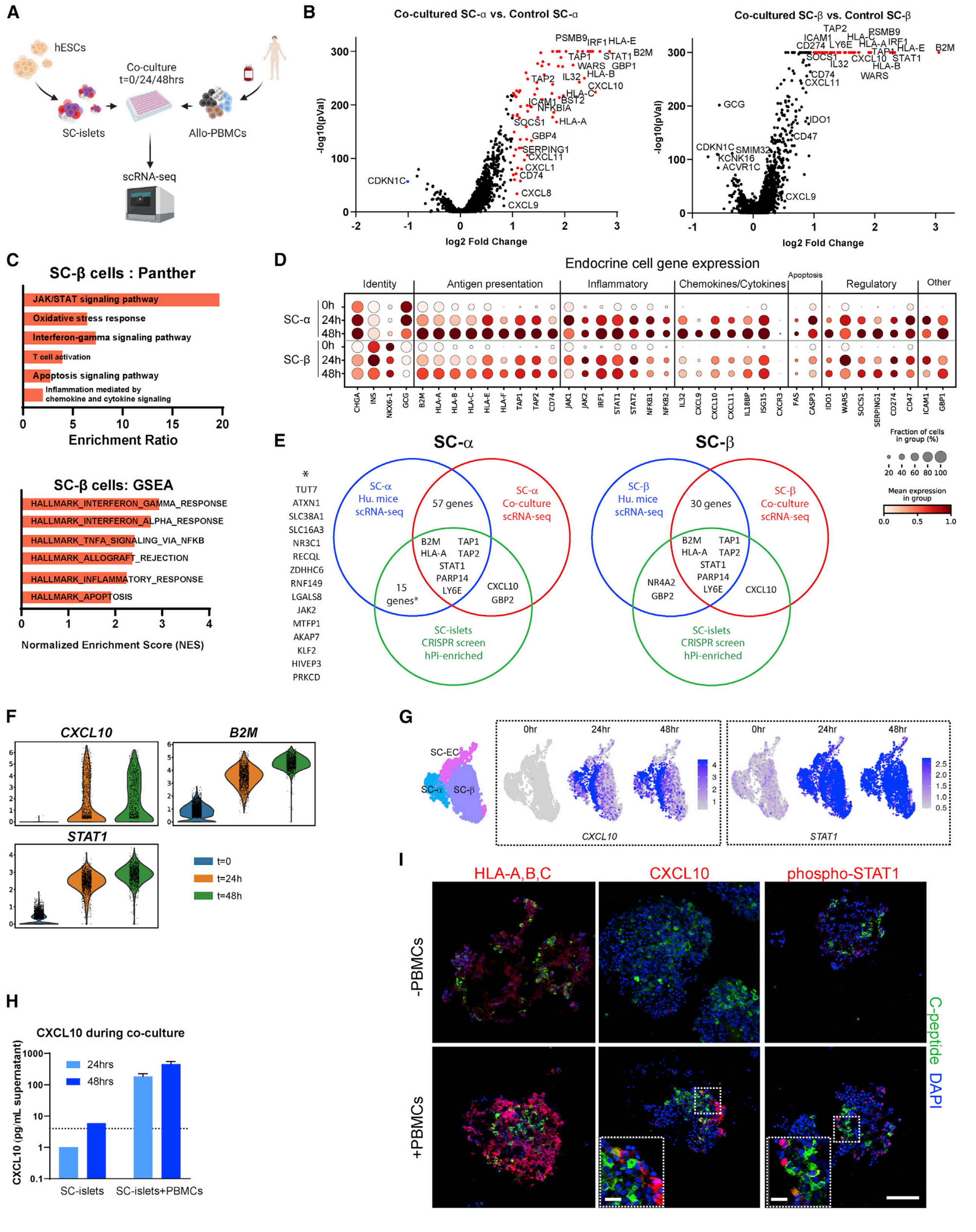

(legend on next page)

Apart from the canonical mediators of the IFN pathway (*STAT1* and *JAK1/2*), other notable hPi-enriched perturbations are *HNRNPA1P48*, *GBP2*, and *TRIM8*. hnRNP proteins are involved RNA processing and splicing (Clarke et al., 2021). *GBP2* is an IFNγ-induced GTPase involved in protective immunity against microorganisms (Tretina et al., 2019) and is also a marker for an efficient T cell response in breast carcinomas (Godoy et al., 2014). TRIM8 is a RING finger protein that inhibits the JAK/STAT suppressor SOCS1 (Toniato et al., 2002), and therefore might act as a IFNγ pathway inducer.

The bottom of Figure 1G shows gene hits that are beneficial to graft survival under immune infiltration of PBMCs. Artificially expressing these genes may help slow or prevent immune destruction. One example is *PTPRA*, a negative regulator of JAK/STAT signaling (Gurzov et al., 2015; Stanley et al., 2015). The difference of *PTPRA* gRNA counts between hPi and control graft is larger than that observed in NT gRNAs, emphasizing the essentiality of PTPRA for graft survival (Figure 1H). Another tyrosine phosphatase, *PTPN2*, is a T1D risk gene (Barrett et al., 2009; Espino-Paisan et al., 2011) but was ranked lower as a beneficial gene in our screen (Figure 1H). In addition, suppressor of cytokine signaling 1 (*SOCS1*), also a negative regulator of JAK/STAT (Galic et al., 2014; Solomon et al., 2011), was upregulated in our scRNA-seq data (Figures 1E and 1F) and exhibited potency as a tolerizing gene (Figure 1H). Other examples that showed a protective effect include small ubiquitin-like modifier 1 (*SUMO1*), which inhibits STAT1 (Rogers et al., 2003), and the tolerizing surface molecule PD-L1 (*CD274*) (Castro-Gutierrez et al., 2021; Yoshihara et al., 2020). *IL32*, *ICAM1*, and *PRDX1* are known to be pro-inflammatory in other systems (Min et al., 2018; Ribeiro-Dias et al., 2017; Yonekawa and Harlan, 2005) and it is unclear why their gRNAs were hPi depleted.

SC-islets are responsible for early-stage immune cell activation through alarm genes

To compensate for limitations of the hPi-mouse model (Shultz et al., 2019) and for unassessed early events (grafts that are retrieved at week 10), we performed an *in vitro* co-culture of allogeneic PBMCs and SC-islet clusters. SC-islet clusters were enriched for β cells (using CD49A magnetic sorting; SC-α and SC-EC still remain at lower numbers) (Veres et al., 2019), dissociated and reaggregated to obtain a more uniform cell count between wells. SC-islets were co-cultured with human allogeneic PBMCs for 24 or 48 h. As controls (time [t] = 0), SC-islets remained in culture without PBMC addition. These samples, in addition to PBMCs alone (t = 0), were used for scRNA-seq (Figure 2A). Prior to co-culture, all SC-islets (controls included) were treated with thapsigargin to enhance and accelerate T cell activation by inducing an ER stress response that was previously shown to recapitulate aspects of autoimmunity (Leite et al., 2020). Differential expression analysis of integrated data from all samples focused on cell populations of interest (Figures S2A–S2C).

CD4, CD8 T cells, and NK cells, at 24- and 48-h co-culture with SC-islets, displayed gene expression profiles of immune activation compared with control (Figure S2D; Tables S1–S3). Transcripts for T cell co-stimulation molecules (including *CD28*, *CD58* [LFA-3], *CD40LG*, *TNFRSF9* [4-1BB], *TNFRSF4* [OX40]) and other activation markers (*IL2RA* [CD25], *CD38*) are upregulated in T cells as well as inhibitory and exhaustion markers (*HAVCR2* [TIM-3], *LAG3*, *PDCD1* [PD-1]) (Figure S2D top). Co-inflammatory cytokines (*IFNG* and *TNF*) and chemokines (*XCL1/2*) are expressed over time in NK and T cells, while anti-inflammatory cytokines (*IL10* and *TGFB1*) are either undetected or downregulated. T cells and NK sensitization to pro-inflammatory chemokines was increased based on elevated levels

Figure 2. Early response of immune-challenged SC-islets profiled by single-cell transcription analysis after co-culture with human allogeneic PBMCs

(A) hESC-derived SC-islets were co-cultured with human allogeneic PBMCs (n = 2 donors) for 0, 24, and 48h, followed by scRNA-seq for gene expression.

(B) Volcano plot of differential expressed genes in SC-α or SC-β after 24-h co-culture with PBMCs compared with control (t = 0).

(C) Pathway analysis and gene set enrichment analysis (GSEA) of upregulated genes in co-cultured SC-β (48 h).

(D) Dot plot representing expression of selected inflammatory genes in groups of SC-α and SC-β over time in co-culture with PBMCs.

(E) Venn diagrams feature significantly upregulated genes (log2 fold change >1 and adjusted p values <0.05) obtained from *in vivo* (blue) and *in vitro* (red) SC-α/SC-β scRNA-seq data (Figures 1 and 2) that are common to CRISPR screen hits (positively enriched in hPi-mice, log2 fold change >1) (green).

(F) Violin plots of SC-β timed expression of selected genes. See also Figure S2F.

(G) UMAP plots of SC-islet cells expressing *CXCL10* or *STAT1* over time in co-culture with PBMCs. Specific endocrine cell type clustering is indicated.

(H) ELISA for human CXCL10, from supernatant of co-culture of SC-islets and PBMCs. n = 2 donors. Error bars are mean ± SD. Dashed line is the lower detection limit, while any data below it is extrapolated.

(I) IF staining of SC-islet clusters ±48-h co-culture with PBMC. C-peptide staining (green) for SC-β and DAPI (blue) for nuclei. Bars represent 100 μm in main panels and 50 μm in magnified panels.

of *CXCR3*, a chemokine receptor that binds CXCL9/10/11 (Figure S2D center). Other prominent transcripts are those that play a part in CTL (cytotoxic T lymphocyte) and NK killing functions (Figure S2D bottom: *PRF1*, *GZMB*, *FASLG*), further indications of an allogeneic response in this co-culture system.

We focused on gene expression in SC-α and SC-β cells compared with controls without PBMC addition. Similar to what was observed for the *in vivo* analysis (Figure 1), upregulated profiles did not differ between co-cultured SC-α and SC-β (Figure 2B) and consisted of clear IFN responses through the JAK/STAT pathway with implications for T cell activation (*B2M*, HLA-I genes), inflammation (e.g., *NFKB1/2*), apoptosis signaling (*FAS*, *CASP3*), and allo-rejection (Figures 2C, 2D, S2E and Tables S1–S3; pathway analysis, and gene set enrichment analysis (GSEA)).

The *in vitro* and *in vivo* experiments described (Figures 1 and 2) point to the conclusion that JAK/STAT signaling in SC-islets is a direct and early consequence of IFN signals received from PBMCs. The unbiased whole-genome screening provides further confirmation of IFN signaling as a critical signaling cascade. We compare readouts from these assays in Figure 2E and find seven common genes upregulated in immune-challenged SC-β and SC-α, engrafted or co-cultured. These genes reflect the widely known importance of antigen processing (*TAP1/2*) and presentation (*B2M*, *HLA-A*) by MHC class I in the initiation of immune responses. *STAT1* links the external signal of IFNγ (also IFNα and β) receptors with the downstream effect that consist of MHC-I stimuli, and secreted agents like CXCL10.

In vivo, very few SC-islets cells continue to express *CXCL10* at week 10, while other ISGs maintain or increase their levels in both experimental models (Figures 1E, 1F, and S1L). Comparatively, *CXCL10* was one of the top upregulated genes in co-culture, slightly more in SC-α than in SC-β cells (Figures 2B and 2D center and 2F). These results, following the CRISPR screen (Figure 1), provide further evidence that CXCL10 is essential for an IFN-triggered immune response. Other chemokines, *CXCL9* and *CXCL11*, were also upregulated in SC-islets *in vitro*. Chemokine signaling may contribute to the early inflammatory response that was missed due to the graft retrieval timing in our *in vivo* model (Figure 1). It is also possible that CXCL10-expressing cells in the SC-islet grafts are eliminated in the PBMC-injected mice. Regardless, CXCL10 is upregulated in parts of the co-cultured SC-islet endocrine population in scRNA-seq analysis (up to 3-fold in SC-β, high versus low CXCL10 cells) and immunofluorescent staining (Figures 2G and 2I) and can be attributed to IFNγ induction (Figure S2G). Furthermore, higher CXCL10 levels are detected in co-culture supernatants compared with SC-islets only (Figure 2H). In all, CXCL10 appears to have a pivotal role in early alloimmune responses.

Given that the JAK/STAT pathway is highly upregulated in SC-islets during co-culture with PBMCs, we examined genes that activate this pathway, along with the IFNγ receptor, intracellular regulator *STAT1*, negative regulator *SOCS1*, and downstream effectors *B2M* and *CD274* (Figures 2F and S2F). STAT1, a master regulator of the JAK/STAT pathway (Gurzov et al., 2016), is enriched in a GSEA transcription factor motif analysis (Figure S2E). Further evidence for the pathway importance in SC-islet immunogenicity comes from co-culture and external IFN stimuli, wherein STAT1 is phosphorylated and translocated to the nuclei of SC-islet cells, and transcription of IFN response elements are induced (Moore et al., 2011) (Figures 2G, 2I, S2G, and S2H).

CXCL10 affects SC-islet immunogenicity

To assess CXCL10 as a target for genetic manipulation compared with other known tolerizing perturbations (β2M KO and PD-L1 overexpression), we co-cultured human allogeneic PBMCs with SC-islets that had been transduced with lentivirus vectors (Figure 3A). For gene KO, vectors expressed Cas9 and gRNAs to *CXCL10* and *B2M*. Overexpression (OE) vectors expressed either *CXCL10* or PD-L1 (*CD274*). All perturbations of target protein expression were assessed compared with NT gRNA or eGFP OE under IFNγ stimuli (Figure S3A). At 48 h after co-culture, SC-islets were stained for apoptotic markers with the focus on SC-β viability (C-peptide staining) (Figure 3B). *CXCL10* and β2M depletions improved viability of SC-β under immune attack by PBMCs (Figure 3B) by more than 2-fold. In addition, a destructive effect of CXCL10 overexpression in SC-β cells under immune attack can be seen by the 50% increase of apoptosis in SC-β overexpressing CXCL10, compared with eGFP overexpression (and comparable with PD-L1) (Figure 3B). PBMCs, prelabeled with cell trace violet to measure proliferation rates, showed reduced T cell proliferation when co-cultured with CXCL10-depleted SC-islets, compared with NT (Figure S3B). Reduced CXCL10 secretion in CXCL10 KO co-cultures was observed (Figure S3C).

CXCR3 is a chemokine receptor expressed on T helper cells, CD8 T cells, NK cells, and monocytes that react with IFN-inducible chemokines, CXCL9/10/11. CXCR3 has a role in chemotaxis and cell proliferation signals (Loetscher et al., 1996) and can also influence T cell polarization to a specific effector linage (Wildbaum et al., 2002). To evaluate the CXCL10-CXCR3 interaction in SC-islet immunogenicity, PBMC and SC-islet co-culture experiments were performed with a blocking antibody to CXCR3 (Figure 3C). Anti-CXCR3 Ab treatment prior to co-culture with SC-islet reduced T cell activation (CD25

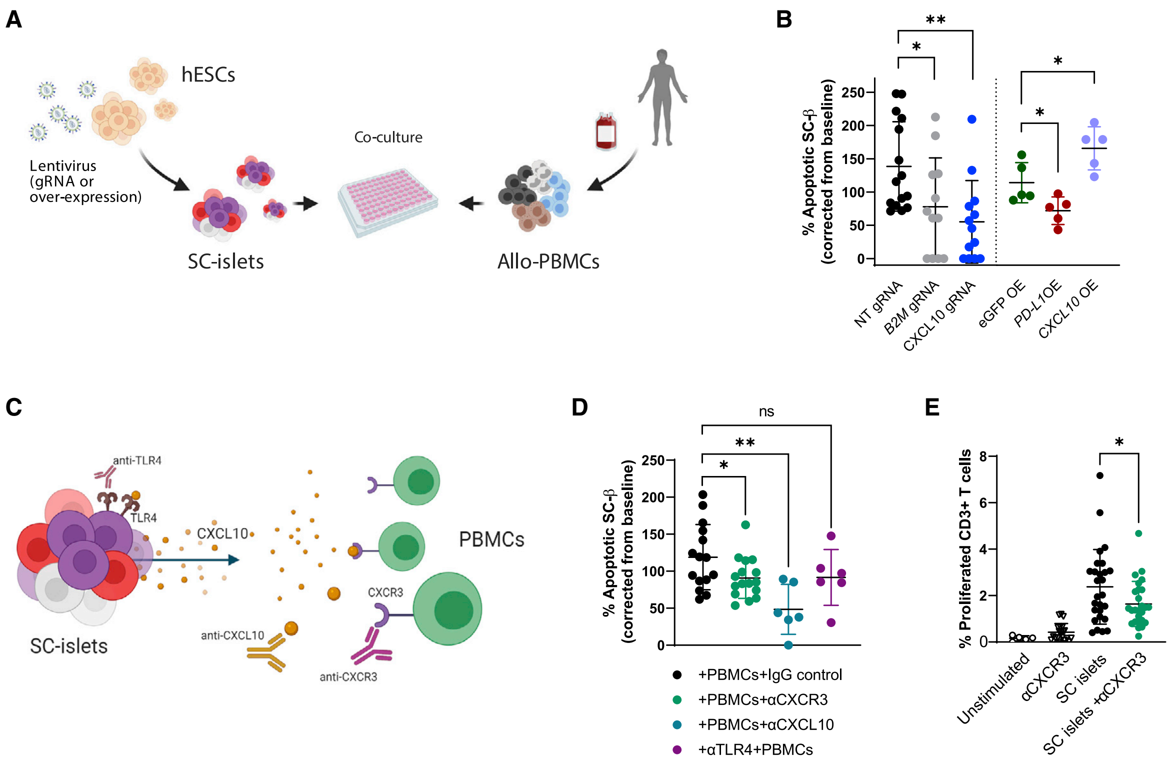

Figure 3. Immunogenicity of CXCL10 expressing SC-islets

(A) Transduced SC-islets with lentiviruses carrying Cas9 + gRNA (KO) or a given open reading frame (ORF) insert (overexpression [OE]), were co-cultured with allogeneic PBMCs.

(B) Flow cytometry for %TUNEL+ (apoptotic) SC-β cells (C-peptide+), following 48-h PBMC co-culture. Apoptosis was calculated by fraction from baseline (%TUNEL without PBMC). gRNA lentivirus transduced SC-islets were compared with non-targeting (NT) gRNA, and OE transduced SC-islets were compared with eGFP OE. n = 3 for ×5 PBMC donors (left; KO), n = 2–3 for ×2 donors (right; OE).

(C) Blocking antibodies prior to/with co-cultures: PBMCs with anti-CXCR3, or SC-islets with anti-TLR4, or anti-CXCL10 during co-culture.

(D) Flow cytometry analysis for apoptotic SC-β, following 48-h PBMC co-culture. n = 3 for ×2–6 donors.

(E) PBMCs were labeled with cell trace violet (CTV) prior to co-culture. Following a 48-h co-culture, PBMCs were separated and allowed to grow for 7 days. $CD3^{+}$ were gated for the CTV-negative fraction of divided cells. n = 5 for ×3 donors. Error bars are mean ± SD. ns, not significant; *p < 0.05; **p < 0.01; ***p < 0.001, unpaired two-tailed t test.

and CD69 activation marker staining), proliferation and the subsequent SC-β apoptotic effect (Figures 3D, 3E, and S3D). An anti-CXCL10 neutralizing antibody added during co-culture also improved SC-β viability (Figure 3D). Since CXCL10 is thought to induce apoptosis through binding to Toll-like receptor 4 (TLR4) in β cells (Schulthess et al., 2009), we treated SC-islets (pre-co-culture) with a TLR4 blocking antibody, which did not significantly reduce apoptosis in this assay (Figure 3D). Overall, these results point to T cell-mediated SC-islet killing through CXCR3 induction, led by CXCL10.

Immunogenicity of *CXCL10* and *STAT1* KO hESC lines assessed *in vitro*

In the light of aforementioned results, two Hues8 hESCs CRISPR KO lines, *CXCL10* KO and *STAT1* KO, were generated with the rationale of diminishing IFN signaling through a master regulator (STAT1) or by confining the effect to one downstream mediator (CXCL10).

Null mutations were created for CXCL10-GFP (C10G) and STAT1-luciferase (ST1L) lines by homology directed repair (HDR) (see section "experimental procedures;" Figures 4A, 4B, and S4A). KO lines displayed normal karyotypes (Figure S4B) and pluripotency marker expression (Figure S4C). These KO lines were compared with a wild-type (WT) Hues8 line or a luciferase expressing Hues8 line (GAPDH-luciferase [GL]; (Gerace et al., 2021)) as controls. C10G, ST1L, and control lines were differentiated successfully into SC-islets (Pagliuca et al., 2014; Veres et al., 2019) and exhibited glucose-stimulated insulin secretion (GSIS) in transplanted mice (Figures S4D–S4F).

C10G SC-islets had very low levels of intracellular CXCL10 staining and almost undetectable CXCL10 secretion after IFNγ stimulation (Figures 4C and 4D). IFNγ

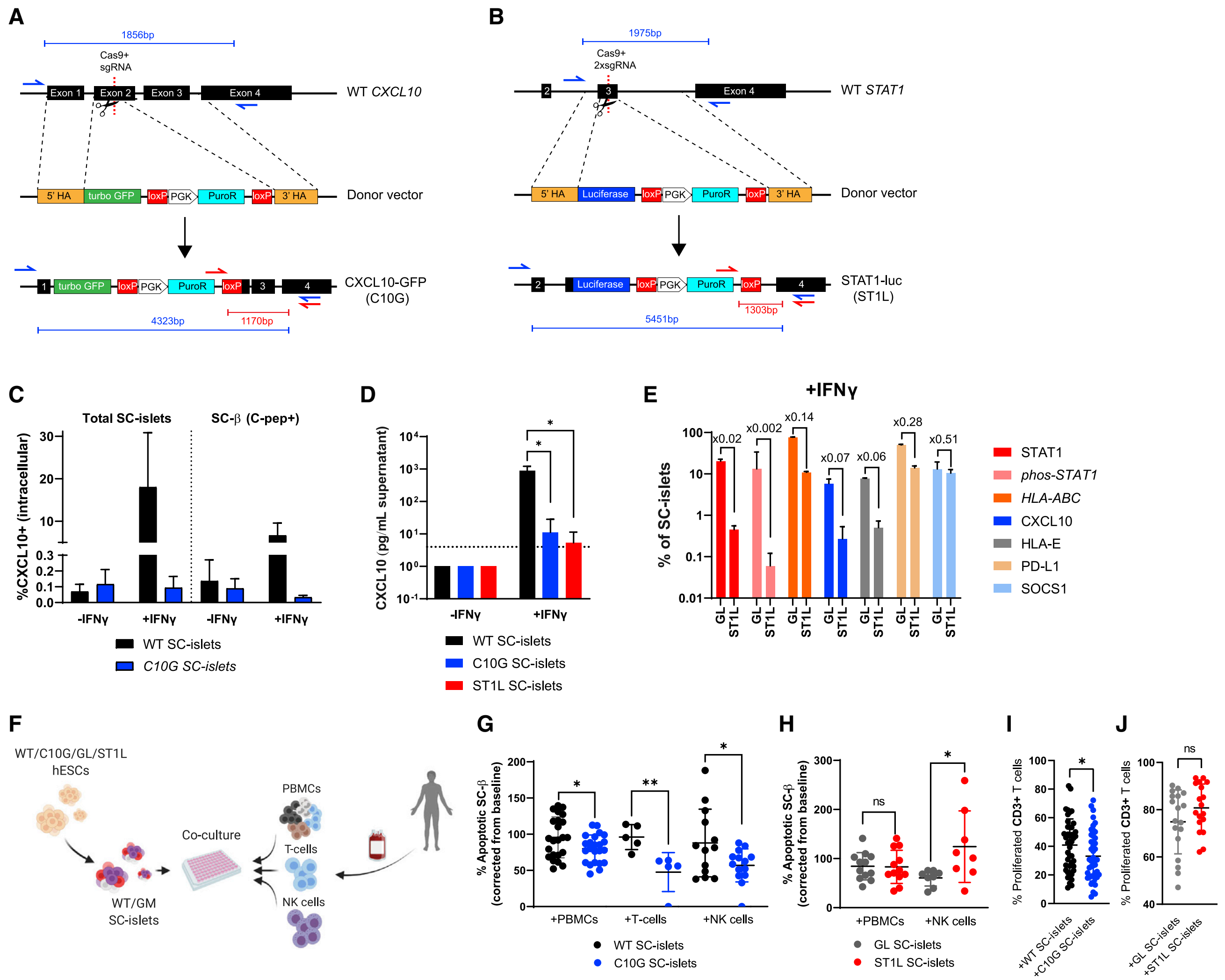

Figure 4. Generation and performance of *CXCL10* KO and *STAT1* KO hESC lines

(A and B) Scheme of targeting the (A) CXCL10 or (B) STAT1 locus in hESCs using CRISPR. Red and blue arrows are PCR primers for genotyping as shown in Figure S4A.

(C) Flow cytometry of intracellular CXCL10 protein in WT/C10G SC-islets and SC-β (C-peptide+) ± rhIFNγ for 48 h n = 3–5.

(D) CXCL10 ELISA of supernatants from ±rhIFNγ-treated WT/C10G/ST1L SC-islets. Dashed line is the lower detection limit, while any data below it is extrapolated.

(E) Flow cytometry for protein expression in rhIFNγ-treated GAPDH-luciferase (GL) or ST1L SC-islets. n = 3–4.

(F–J) Gene-modified (GM; C10G/ST1L) and control (WT/GL) lines were differentiated into SC-islets, and co-cultured with human PBMCs or purified T cells/NK cells. Apoptosis was calculated by fraction from baseline (%TUNEL without PBMCs). (G) Apoptotic WT or C10G SC-β cells (n = 4 for ×6 PBMC donors, n = 2–3 ×2 T cell donors, n = 4 × 4 NK cell donors).

(H) Apoptotic GL or ST1L SC-β cells (n = 4 for ×2 PBMC or NK cell). (I and J) Proliferated CD3 T cell following co-culture with indicated GM SC-islets (I) n = 9 for ×5 donors and (J) n = 9 for ×2 donors). Error bars are mean ± SD. ns, not significant; $^*p < 0.05$; $^{**}p < 0.01$, unpaired two-tailed t-test.

treatment of GL SC-islets induced phosphorylated STAT1 that was impaired in ST1L SC-islets (Figure 4E). The absence of STAT1 in ST1L also led to desensitization to IFNγ, as shown by the downregulation of HLA proteins and CXCL10 as well as inhibitory proteins (HLA-E, PD-L1, and SOCS1) (Figures 4D and 4E).

Gene-modified (GM) and control SC-islets were co-cultured with allogeneic PBMCs. To evaluate the contribution of specific immune populations on SC-islet killing, we also co-cultured GM SC-islets with blood purified T cells (C10G only) and NK cells (Figure 4F). Compared with WT, C10G co-cultures displayed significant protective

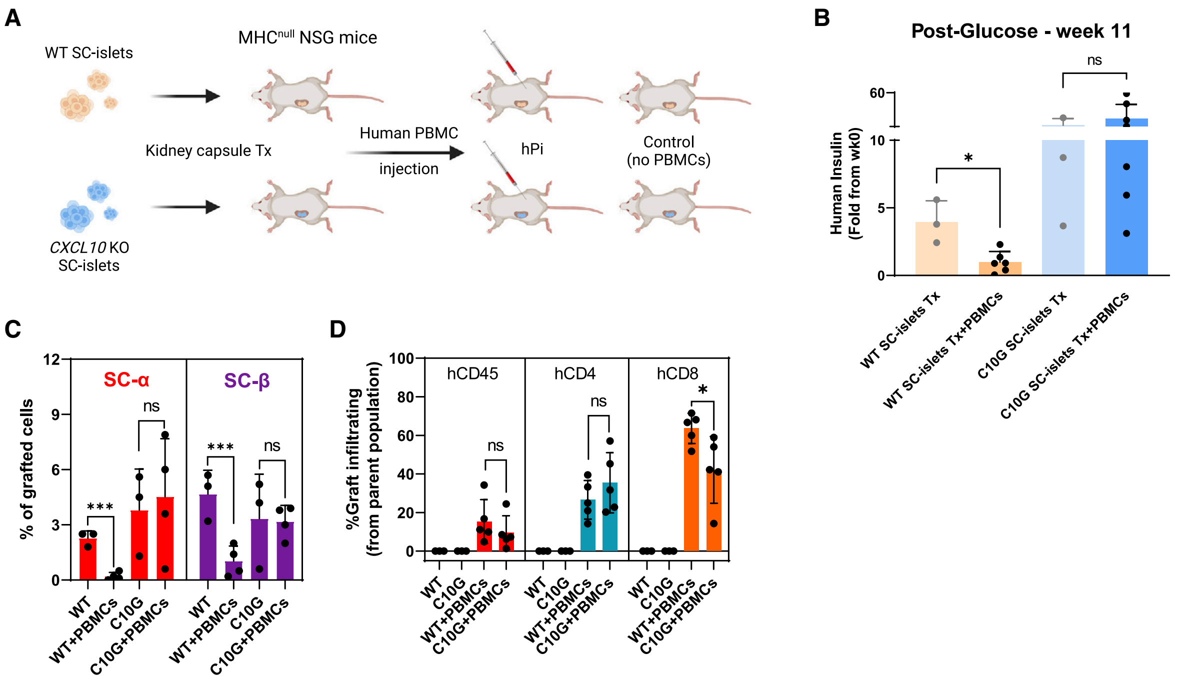

Figure 5. *CXCL10* KO SC-islet grafts evade alloimmune attack in humanized mice

(A) WT or C10G SC-islets were transplanted into MHC^{null} NSG mice (n = 10 from each line). n = 6–7 mice from each group injected with human PBMCs (n = 2 human donors), while the remainder served as control (n = 3 per group).

(B) Graft failure at week 11 after PBMC injections, as measured by human insulin in fasted mice plasma, 30 min after glucose injection to fasted mice. Data presented as fold increase from t = 0 before PBMC injections.

(C) Flow cytometry of SC-α (glucagon+/C-peptide−) and SC-β (glucagon−/C-peptide+) in extracted grafts at week 18 post PBMC injection. n = 3–4 mice per group.

(D) Flow cytometry of human T cells in hPi-mouse graft infiltrating at week 18 post PBMC injection. n = 3–5 mice per group. Error bars are mean ± SD. ns, not significant; *p < 0.05; **p < 0.01, unpaired two-tailed t test.

performances against allo-PBMCs, T cells, and NK cells based on improved SC-β (Figure 4G) and SC-islet (Figure S4G) viability and reduced activation and proliferation of T cells (Figures 4I and S4H) in co-cultured PBMCs. In contrast, ST1L did not significantly reduce the response to PBMCs, and more SC-islets were apoptotic after NK cell co-culture (Figures 4H and S4G). T cells from ST1L and GL control SC-islets, co-cultured with PBMCs, show the same level of activation and proliferation (Figures 4J and S4I). Diminished inhibitory signals such as PD-L1 and SOCS1 (Figure 4E) may explain why ST1L does not reduce the immune response to PBMCs, and the reduced expression of HLA class I may be the cause for increased NK killing (Figures 4H and S4G).

CXCL10-deficient SC-islets are hypoimmunogenic *in vivo*

Since full *STAT1* depletion (ST1L) shows unimpressive results in reducing the immune response *in vitro* (Figures 4H and 4J), we focused on C10G for *in vivo* studies.

Using the *in vivo* model (Figure 1), C10G or WT SC-islets were transplanted (n = 20), followed by PBMC injection (hPi) from two human donors, leaving three mice in each group without PBMC injection as controls (Figure 5A). Beginning at week 11 after PBMC injection, graft failure was observed in hPi-mice transplanted with WT SC-islets, continuing through week 17, whereas WT control grafts remained functional. Interestingly, C10G SC-islet graft insulin levels remained stable and even increased over time, with no significant difference between hPi and control mice (Figures 5B and S5A). At the end of the experiment (week 17 post PBMC), kidney capsule grafts were extracted and stained for endocrine and T cell markers. Consistent with insulin measurements (Figure 5B), we observed a decline in the number of SC-β (and SC-α) in WT hPi grafts, but not in C10G hPi grafts compared with controls (Figure 5C). The improved survival of SC-islets can be attributed to the lower frequency of infiltrating human CD8 T cells, comparing C10G hPi grafts with WT hPi grafts (Figure 5D), while circulating human lymphocyte levels did not change (Figure S5B).

In all, SC-islets with impaired ability to express CXCL10 are not only hypoimmunogenic *in vitro* (Figure 4) but are also cable of evading immune attack *in vivo* within an allograft.

DISCUSSION

This study used two approaches to reveal genes that drive SC-islet immunogenicity: transcript analysis characterized the responses to immune challenge, and CRISPR genome screening helped assess the cause of those responses.

In responding to allogeneic immune cells, the strongest effect in SC-islets is upregulation of ISGs. The results show that T cells are activated in immune environments and express IFNγ, among many other inflammatory genes. The secreted IFNγ leads to an inflammatory cascade in which ISGs are upregulated in SC-islets. A plausible explanation for T cell activation is by antigen presentation through MHC class I molecules.

The most striking observation was the involvement of chemokines secreted by SC-islets. These results suggest that CXCL10 has a role in the early stage of immune-graft interaction. *CXCL10*-KO SC-islet cells in an allogeneic *in vivo* model survived longer compared with surrounding cells with other perturbations. Furthermore, the *in vitro* and *in vivo* allogeneic experiments demonstrate that CXCL10-deficient SC-islets are immune evasive compared with WT. *CXCL10*-deficient SC-islets (C10G) have 2-fold increased survival capability under immune challenge by allogeneic T cells or NK cells. Upon engraftment in a humanized allogeneic *in vivo* model, C10G avoid immune destruction 7 weeks longer than WT SC-islets. CXCL10 as a secreted chemokine plays a determining role as a recruiter of immune cells to an SC-islet transplant site, and depleting it keeps those grafts out of the reach of a human immune system.

CXCL10 is one of the most upregulated chemokines in primary human islets (Eizirik et al., 2012) and hPSC-derived islets (Demine et al., 2020; Dettmer et al., 2022) under pro-inflammatory conditions. Islets of recent-onset T1D show CXCL10 expression in regions where infiltrating lymphocytes express CXCR3 (Roep et al., 2010; Uno et al., 2010). Our results show that CXCL10 expression is not exclusive to SC-β cells but is also differentially expressed by other SC-endocrine cells. A recent study also demonstrated the contribution of pancreatic α cells to CXCL10 expression in NOD mice and in recent-onset T1D islets (Nigi et al., 2020).

In our previous study using a T1D autologous *in vitro* model, CXCL10 was highly secreted from iPSC-islets during co-culture with matched T1D PBMCs (Leite et al., 2020). In this current work, CXCL10 expression was seen in co-cultures but not in late stages of graft rejections, supporting the view of CXCL10 as a first responder or alarm protein at the onset of SC-islet interactions with a hostile immune system. In T1D, islet CXCL10 expression occurs in early stages (Roep et al., 2010; Uno et al., 2010) and serum levels of CXCL10 are elevated in recent-onset compared with long-term T1D individuals (auto-Ab+) (Shimada et al., 2001). Mouse islet isografts expressed high levels of Cxcl10 at day 2 after transplantation into diabetic C57BL/6 mice, but in a lesser degree by day 100 (Bender et al., 2017). Furthermore, analysis of plasma samples from human islet transplant patients revealed that CXCL10 was among the highest released inflammatory mediators and peaked 24 h post transplantation (Yoshimatsu et al., 2017).

In T1D, pancreatic islets react to pro-inflammatory cytokines by inducing the NF-κB and STAT1 signaling that contribute to the immune destruction mechanism of β cells (Cnop et al., 2005; Eizirik et al., 2012). Although our experiments were done in an allogeneic setting, both transcription factors were upregulated in SC-islets, but only *STAT1* depletion showed up as a hit in the CRISPR screen. However, when *STAT1* KO (ST1L) SC-islets were used, this rescue was not reproduced (Figure 4). The reason might derive from the observation that STAT1-deficient SC-islets lose immune-inducing elements such as HLA molecules and CXCL10, but also suffer from loss of immune-inhibitory functions like PD-L1 and SOCS1.

Downstream to STAT1 is the transcription factor IRF1, which has anti-inflammatory effects in β cells through the induction of SOCS1 (Moore et al., 2011). SOCS1 and PTPN2 are negative regulators of cytokine signaling (Chong et al., 2002; Elvira et al., 2022; Moore et al., 2009) and are both associated with T1D risk loci (Onengut-Gumuscu et al., 2015; Ram and Morahan, 2017). Previous reports have shown that SOCS1 overexpression in NOD mice islets prevent diabetes (Flodstrom-Tullberg et al., 2003), and delays allogeneic islet graft rejection in mouse models (Solomon et al., 2011). Our data show that, under PBMCs + SC-islet interactions, both *IRF1* and *SOCS1* are differentially upregulated. *SOCS1* KO and *PTPN2* KO SC-islets were depleted in hPi grafts in our CRISPR screen, along with *PTPRA* KO, another PTP family member (Stanley et al., 2015).

Based on ST1L's unconvincing results (Figure 4), "pan-JAK/STAT" diminishing strategies should be considered cautiously. These approaches include *SOCS1* overexpression and *IFNGR1* KO. Transgenic lines of *SOCS1* OE or *IFNGR1* KO might have consequences of losing the inflammatory negative regulation feedback of JAK/STAT signaling. PD-L1 downregulation under JAK/STAT silencing will expose SC-islets to T cell attack, while HLA downregulation will result in NK cell recognition and killing. It may be useful to co-edit such stem cell lines with additional modification(s) that will address these concerns.

The analyses presented in this paper include many other genes that may be targeted to control the immune response against SC-islets. Modulation of ISGs by identified hits from our *in vivo* CRISPR screen (e.g., *TRIM8*, *SUMO1*), or others of unclear function (e.g., *IL32*, *CAMSAP3*), were not considered here but may have the potential to reduce immunogenicity. Nevertheless, this study points to opportunities for future applications of SC-islet as a cell replacement therapy for T1D.

Limitations of the study

An optimal pooled screen would be one that relies on a robust assay with a selection force that separates cells using a phenotype of interest (Doench, 2018). Although we were able to acquire gene hits from the described *in vivo* CRISPR screen, the assay (hPi model) is not flawless. T cells are the only immune cells that engraft successfully and persist long term, leaving out other immune cells that may also contribute to SC-islet graft destruction, in particular NK cells (Shultz et al., 2019). In addition, pooled screens can benefit from survival selection of cells that could proliferate and amplify the enrichment signal. The enrichment in our screen is based solely on differentiated post-mitotic cells.

EXPERIMENTAL PROCEDURES

Contact for reagent and resource sharing

Further information and requests for resources and reagents should be directed to and will be fulfilled by the corresponding author, Douglas A. Melton (dmelton@harvard.edu).

Experimental model and subject details

All procedures were performed in accordance with the Institutional Review Board (IRB) guidelines at Harvard University under IRB and Embryonic Stem Cell Research Oversight Committee (ESCRO) protocol E00024. All animal experiments were performed in accordance with Harvard University International Animal Care and Use Committee regulations.

Quantification and statistical analyses

Statistical analysis was performed by unpaired Student's t tests as indicated, using Prism v9. All data are presented mean ± SD. $p < 0.05$ was considered statistically significant. Sufficient sample size was estimated without the use of a power calculation. Data analysis was not blinded.

Graphic illustrations

Graphic illustrations in the manuscript were created with BioRender.com under BioRender's academic license terms.

Data and code availability

scRNA-seq and pooled CRISPR screen data generated during this study are available at NCBI (GEO: GSE200104) and are composed of listed sub-series related to specific experiments described in this paper.

SUPPLEMENTAL INFORMATION

Supplemental information can be found online at https://doi.org/10.1016/j.stemcr.2022.08.002.

AUTHOR CONTRIBUTIONS

E.S. conceived the study. E.S., J.HR.K., A.S.A., and K.B. performed the experiments. D.G. was involved in the experimental design and provided technical support. I.N., V.B., and S.H.S. analyzed the scRNA-seq data. I.N. and E.S. analyzed the CRISPR screen data. E.S. and D.A.M. wrote the manuscript. D.A.M. designed and supervised the research.

ACKNOWLEDGMENTS

We thank J. Babon, R. Pop, N. Sharon and D. Bavli for helpful discussions and feedback on the manuscript; E. Engquist, Y. Yu, K. Biles, J. Zhang, E. Robinson, M. Hinderhofer, and Kyle Boulanger for technical support in stem cell culture maintenance and differentiation; N. Ramirez and C. Gerhardinger from the Bauer Core Facility at Harvard University for 10X support; E. Rosardo-Oliveiri and A. Veres for help with scRNA-seq analysis; and J. Doench and the Genetic Perturbation Plat-form of the Broad Institute for CRISPR screen advice and support. D.A.M. is an investigator of the Howard Hughes Medical Institute (HHMI). E.S. is supported by a JDRF post-doctoral fellowship (3-PDF-2018-590-A-N). V.B. and S.H.S. were supported in part by the Harvard Medical School (HMS) Foundry and Harvard Catalyst (UL 1TR002541). Computations in this paper were run on the HMS O2 cluster supported by the HMS Research Computing Group. This work was supported by grants from the Harvard Stem Cell Institute, Helmsley Charitable Trust (2015PG-T1D044), JDRF (5-SRA-2014-284-Q-R), and the JPB Foundation (award #1094).

CONFLICTS OF INTEREST

D.A.M. is a founder of Semma Therapeutics and an employee of Vertex Pharmaceuticals, which has licensed technologies from Harvard and HHMI. E.S. and K.B. are now Vertex employees. All other authors declare no competing interests. A patent related to this work was filed by Harvard University.

Received: July 13, 2022
Revised: August 5, 2022
Accepted: August 6, 2022
Published: September 1, 2022

REFERENCES

Augsornworawat, P., Maxwell, K.G., Velazco-Cruz, L., and Millman, J.R. (2020). Single-cell transcriptome profiling reveals beta cell maturation in stem cell-derived islets after transplantation. Cell Rep. *34*, 108850.

Banting, F.G., Best, C.H., Collip, J.B., Campbell, W.R., and Fletcher, A.A. (1922). Pancreatic extracts in the treatment of diabetes mellitus. Can. Med. Assoc. J. *12*, 141–146.

Barrett, J.C., Clayton, D.G., Concannon, P., Akolkar, B., Cooper, J.D., Erlich, H.A., Julier, C., Morahan, G., Nerup, J., Nierras, C., et al. (2009). Genome-wide association study and meta-analysis find that over 40 loci affect risk of type 1 diabetes. Nat. Genet. *41*, 703–707.

Bender, C., Christen, S., Scholich, K., Bayer, M., Pfeilschifter, J.M., Hintermann, E., and Christen, U. (2017). Islet-expressed CXCL10 promotes autoimmune destruction of islet isografts in mice with type 1 diabetes. Diabetes *66*, 113–126.

Brehm, M.A., Kenney, L.L., Wiles, M.V., Low, B.E., Tisch, R.M., Burzenski, L., Mueller, C., Greiner, D.L., and Shultz, L.D. (2019). Lack of acute xenogeneic graft- versus-host disease, but retention of T-cell function following engraftment of human peripheral blood mononuclear cells in NSG mice deficient in MHC class I and II expression. FASEB J. *33*, 3137–3151.

Businesswire (2021). Vertex Announces Positive Day 90 Data for the First Patient in the Phase 1/2 Clinical Trial Dosed with VX-880, a Novel Investigational Stem Cell-Derived Therapy for the Treatment of Type 1 Diabetes. https://www.businesswire.com/news/home/20211018005226/en/.

Cai, E.P., Ishikawa, Y., Zhang, W., Leite, N.C., Li, J., Hou, S., Kiaf, B., Hollister-Lock, J., Yilmaz, N.K., Schiffer, C.A., et al. (2020). Genome-scale in vivo CRISPR screen identifies RNLS as a target for beta cell protection in type 1 diabetes. Nat. Metab. *2*, 934–945.

Castro-Gutierrez, R., Alkanani, A., Mathews, C.E., Michels, A., and Russ, H.A. (2021). Protecting stem cell derived pancreatic beta-like cells from diabetogenic T cell recognition. Front. Endocrinol. *12*, 707881.

Chang, M.S., McNinch, J., Basu, R., and Simonet, S. (1994). Cloning and characterization of the human neutrophil-activating peptide (ENA-78) gene. J. Biol. Chem. *269*, 25277–25282.

Chong, M.M.W., Thomas, H.E., and Kay, T.W.H. (2002). Suppressor of cytokine signaling-1 regulates the sensitivity of pancreatic beta cells to tumor necrosis factor. J. Biol. Chem. *277*, 27945–27952.

Clarke, J.P., Thibault, P.A., Salapa, H.E., and Levin, M.C. (2021). A comprehensive analysis of the role of hnRNP A1 function and dysfunction in the pathogenesis of neurodegenerative disease. Front. Mol. Biosci. *8*, 659610.

Cnop, M., Welsh, N., Jonas, J.C., Jörns, A., Lenzen, S., and Eizirik, D.L. (2005). Mechanisms of pancreatic beta-cell death in type 1 and type 2 diabetes: many differences, few similarities. Diabetes *54*, S97–S107.

Demine, S., Schiavo, A.A., Marín-Cañas, S., Marchetti, P., Cnop, M., and Eizirik, D.L. (2020). Pro-inflammatory cytokines induce cell death, inflammatory responses, and endoplasmic reticulum stress in human iPSC-derived beta cells. Stem Cell Res. Ther. *11*, 7.

Dettmer, R., Niwolik, I., Cirksena, K., Yoshimoto, T., Tang, Y., Mehmeti, I., Gurgul-Convey, E., and Naujok, O. (2022). Proinflammatory cytokines induce rapid, NO-independent apoptosis, expression of chemotactic mediators and interleukin-32 secretion in human pluripotent stem cell-derived beta cells. Diabetologia *65*, 829–843.

Deuse, T., Hu, X., Agbor-Enoh, S., Jang, M.K., Alawi, M., Saygi, C., Gravina, A., Tediashvili, G., Nguyen, V.Q., Liu, Y., et al. (2021). The SIRPalpha-CD47 immune checkpoint in NK cells. J. Exp. Med. *218*, e20200839.

Deuse, T., Hu, X., Gravina, A., Wang, D., Tediashvili, G., De, C., Thayer, W.O., Wahl, A., Garcia, J.V., Reichenspurner, H., et al. (2019). Hypoimmunogenic derivatives of induced pluripotent stem cells evade immune rejection in fully immunocompetent allogeneic recipients. Nat. Biotechnol. *37*, 252–258.

Doench, J.G. (2018). Am I ready for CRISPR? A user's guide to genetic screens. Nat. Rev. Genet. *19*, 67–80.

Doench, J.G., Fusi, N., Sullender, M., Hegde, M., Vaimberg, E.W., Donovan, K.F., Smith, I., Tothova, Z., Wilen, C., Orchard, R., et al. (2016). Optimized sgRNA design to maximize activity and minimize off-target effects of CRISPR-Cas9. Nat. Biotechnol. *34*, 184–191.

Eizirik, D.L., Sammeth, M., Bouckenooghe, T., Bottu, G., Sisino, G., Igoillo-Esteve, M., Ortis, F., Santin, I., Colli, M.L., Barthson, J., et al. (2012). The human pancreatic islet transcriptome: expression of candidate genes for type 1 diabetes and the impact of pro-inflammatory cytokines. PLoS Genet. *8*, e1002552.

Elvira, B., Vandenbempt, V., Bauza-Martinez, J., Crutzen, R., Negueruela, J., Ibrahim, H., Winder, M.L., Brahma, M.K., Vekeriotaite, B., Martens, P.J., et al. (2022). PTPN2 regulates the interferon signaling and endoplasmic reticulum stress response in pancreatic beta-cells in autoimmune diabetes. Diabetes *71*. https://doi.org/10.2337/db21-0443.

Espino-Paisan, L., de la Calle, H., Fernández-Arquero, M., Figueredo, M.A., de la Concha, E.G., Urcelay, E., and Santiago, J.L. (2011). A polymorphism in PTPN2 gene is associated with an earlier onset of type 1 diabetes. Immunogenetics *63*, 255–258.

Flodstrom-Tullberg, M., Yadav, D., Hägerkvist, R., Tsai, D., Secrest, P., Stotland, A., and Sarvetnick, N. (2003). Target cell expression of suppressor of cytokine signaling-1 prevents diabetes in the NOD mouse. Diabetes *52*, 2696–2700.

Galic, S., Sachithanandan, N., Kay, T.W., and Steinberg, G.R. (2014). Suppressor of cytokine signalling (SOCS) proteins as guardians of inflammatory responses critical for regulating insulin sensitivity. Biochem. J. *461*, 177–188.

Gerace, D., Boulanger, K.R., Hyoje-Ryu Kenty, J., and Melton, D.A. (2021). Generation of a heterozygous GAPDH-Luciferase human ESC line (HVRDe008-A-1) for in vivo monitoring of stem cells and their differentiated progeny. Stem Cell Res *53*, 102371.

Godoy, P., Cadenas, C., Hellwig, B., Marchan, R., Stewart, J., Reif, R., Lohr, M., Gehrmann, M., Rahnenführer, J., Schmidt, M., et al. (2014). Interferon-inducible guanylate binding protein (GBP2) is associated with better prognosis in breast cancer and indicates an efficient T cell response. Breast Cancer *21*, 491–499.

Gornalusse, G.G., Hirata, R.K., Funk, S.E., Riolobos, L., Lopes, V.S., Manske, G., Prunkard, D., Colunga, A.G., Hanafi, L.A., Clegg, D.O., et al. (2017). HLA-E-expressing pluripotent stem cells escape allogeneic responses and lysis by NK cells. Nat. Biotechnol. *35*, 765–772.

Gurzov, E.N., Stanley, W.J., Brodnicki, T.C., and Thomas, H.E. (2015). Protein tyrosine phosphatases: molecular switches in metabolism and diabetes. Trends Endocrinol. Metab. *26*, 30–39.

Gurzov, E.N., Stanley, W.J., Pappas, E.G., Thomas, H.E., and Gough, D.J. (2016). The JAK/STAT pathway in obesity and diabetes. FEBS J. *283*, 3002–3015.

Han, X., Wang, M., Duan, S., Franco, P.J., Kenty, J.H.R., Hedrick, P., Xia, Y., Allen, A., Ferreira, L.M.R., Strominger, J.L., et al. (2019). Generation of hypoimmunogenic human pluripotent stem cells. Proc. Natl. Acad. Sci. USA *116*, 10441–10446.

Harding, J., Vintersten-Nagy, K., Shutova, M., Yang, H., Tang, J.K., Massumi, M., Izaidfar, M., ZohrehIzadifar, Zhang, P., Li, C., et al. (2019). Induction of long-term allogeneic cell acceptance and formation of immune privileged tissue in immunocompetent hosts. Preprint at bioRxiv. https://doi.org/10.1101/716571.

Helman, A., and Melton, D.A. (2021). A stem cell approach to cure type 1 diabetes. Cold Spring Harb. Perspect. Biol. *13*, a035741.

Henry, R.R., Pettus, J., Wilensky, J., Shapiro, A.J., Senior, P.A., Roep, B., Wang, R., Kroon, E.J., Scott, M., D'amour, K., and Foyt, H.L. (2018). Initial clinical evaluation of VC-01TM combination product—a stem cell–derived islet replacement for type 1 diabetes (T1D). Diabetes *67*, 138.

Herold, K.C., Bundy, B.N., Long, S.A., Bluestone, J.A., DiMeglio, L.A., Dufort, M.J., Gitelman, S.E., Gottlieb, P.A., Krischer, J.P., Linsley, P.S., et al. (2019). An anti-CD3 antibody, teplizumab, in relatives at risk for type 1 diabetes. N. Engl. J. Med. *381*, 603–613.

Kovatchev, B. (2019). A century of diabetes technology: signals, models, and artificial pancreas control. Trends Endocrinol. Metab. *30*, 432–444.

Lehmann, R., Spinas, G.A., Moritz, W., and Weber, M. (2008). Has time come for new goals in human islet transplantation? Am. J. Transplant. *8*, 1096–1100.

Leite, N.C., Sintov, E., Meissner, T.B., Brehm, M.A., Greiner, D.L., Harlan, D.M., and Melton, D.A. (2020). Modeling type 1 diabetes in vitro using human pluripotent stem cells. Cell Rep. *32*, 107894.

Loetscher, M., Gerber, B., Loetscher, P., Jones, S.A., Piali, L., Clark-Lewis, I., Baggiolini, M., and Moser, B. (1996). Chemokine receptor specific for IP10 and mig: structure, function, and expression in activated T-lymphocytes. J. Exp. Med. *184*, 963–969.

Min, Y., Kim, M.J., Lee, S., Chun, E., and Lee, K.Y. (2018). Inhibition of TRAF6 ubiquitin-ligase activity by PRDX1 leads to inhibition of NFKB activation and autophagy activation. Autophagy *14*, 1347–1358.

Moore, F., Colli, M.L., Cnop, M., Esteve, M.I., Cardozo, A.K., Cunha, D.A., Bugliani, M., Marchetti, P., and Eizirik, D.L. (2009). PTPN2, a candidate gene for type 1 diabetes, modulates interferon-gamma-induced pancreatic beta-cell apoptosis. Diabetes *58*, 1283–1291.

Moore, F., Naamane, N., Colli, M.L., Bouckenooghe, T., Ortis, F., Gurzov, E.N., Igoillo-Esteve, M., Mathieu, C., Bontempi, G., Thykjaer, T., et al. (2011). STAT1 is a master regulator of pancreatic {beta}-cell apoptosis and islet inflammation. J. Biol. Chem. *286*, 929–941.

Nigi, L., Brusco, N., Grieco, G.E., Licata, G., Krogvold, L., Marselli, L., Gysemans, C., Overbergh, L., Marchetti, P., Mathieu, C., et al. (2020). Pancreatic alpha-cells contribute together with beta-cells to CXCL10 expression in type 1 diabetes. Front. Endocrinol. *11*, 630.

Nostro, M.C., Sarangi, F., Yang, C., Holland, A., Elefanty, A.G., Stanley, E.G., Greiner, D.L., and Keller, G. (2015). Efficient generation of NKX6-1+ pancreatic progenitors from multiple human pluripotent stem cell lines. Stem Cell Rep. *4*, 591–604.

Onengut-Gumuscu, S., Chen, W.M., Burren, O., Cooper, N.J., Quinlan, A.R., Mychaleckyj, J.C., Farber, E., Bonnie, J.K., Szpak, M., Schofield, E., et al. (2015). Fine mapping of type 1 diabetes susceptibility loci and evidence for colocalization of causal variants with lymphoid gene enhancers. Nat. Genet. *47*, 381–386.

Orban, T., Bundy, B., Becker, D.J., DiMeglio, L.A., Gitelman, S.E., Goland, R., Gottlieb, P.A., Greenbaum, C.J., Marks, J.B., Monzavi, R., et al. (2011). Co-stimulation modulation with abatacept in patients with recent-onset type 1 diabetes: a randomised, double-blind, placebo-controlled trial. Lancet *378*, 412–419.

Pagliuca, F.W., Millman, J.R., Gürtler, M., Segel, M., Van Dervort, A., Ryu, J.H., Peterson, Q.P., Greiner, D., and Melton, D.A. (2014). Generation of functional human pancreatic beta cells in vitro. Cell *159*, 428–439.

Parent, A.V., Faleo, G., Chavez, J., Saxton, M., Berrios, D.I., Kerper, N.R., Tang, Q., and Hebrok, M. (2021). Selective deletion of human leukocyte antigens protects stem cell-derived islets from immune rejection. Cell Rep. *36*, 109538.

Platanias, L.C. (2005). Mechanisms of type-I- and type-II-interferon-mediated signalling. Nat. Rev. Immunol. *5*, 375–386.

Raffin, C., Vo, L.T., and Bluestone, J.A. (2020). Treg cell-based therapies: challenges and perspectives. Nat. Rev. Immunol. *20*, 158–172.

Ram, R., and Morahan, G. (2017). Effects of type 1 diabetes risk alleles on immune cell gene expression. Genes *8*, E167.

Ramzy, A., Thompson, D.M., Ward-Hartstonge, K.A., Ivison, S., Cook, L., Garcia, R.V., Loyal, J., Kim, P.T.W., Warnock, G.L., Levings, M.K., et al. (2021). Implanted pluripotent stem-cell-derived pancreatic endoderm cells secrete glucose-responsive C-peptide in patients with type 1 diabetes. Cell Stem Cell *28*, 2047–2061.e5.

Rezania, A., Bruin, J.E., Arora, P., Rubin, A., Batushansky, I., Asadi, A., O'Dwyer, S., Quiskamp, N., Mojibian, M., Albrecht, T., et al. (2014). Reversal of diabetes with insulin-producing cells derived in vitro from human pluripotent stem cells. Nat. Biotechnol. *32*, 1121–1133.

Ribeiro-Dias, F., Saar Gomes, R., de Lima Silva, L.L., Dos Santos, J.C., and Joosten, L.A.B. (2017). Interleukin 32: a novel player in the control of infectious diseases. J. Leukoc. Biol. *101*, 39–52.

Roep, B.O., Kleijwegt, F.S., van Halteren, A.G.S., Bonato, V., Boggi, U., Vendrame, F., Marchetti, P., and Dotta, F. (2010). Islet inflammation and CXCL10 in recent-onset type 1 diabetes. Clin. Exp. Immunol. *159*, 338–343.

Rogers, R.S., Horvath, C.M., and Matunis, M.J. (2003). SUMO modification of STAT1 and its role in PIAS-mediated inhibition of gene activation. J. Biol. Chem. *278*, 30091–30097.

Russ, H.A., Parent, A.V., Ringler, J.J., Hennings, T.G., Nair, G.G., Shveygert, M., Guo, T., Puri, S., Haataja, L., Cirulli, V., et al. (2015). Controlled induction of human pancreatic progenitors produces functional beta-like cells in vitro. EMBO J. *34*, 1759–1772.

Scholz, C., and Tampe, R. (2005). The intracellular antigen transport machinery TAP in adaptive immunity and virus escape mechanisms. J. Bioenerg. Biomembr. *37*, 509–515.

Schulthess, F.T., Paroni, F., Sauter, N.S., Shu, L., Ribaux, P., Haataja, L., Strieter, R.M., Oberholzer, J., King, C.C., and Maedler, K. (2009). CXCL10 impairs beta cell function and viability in diabetes through TLR4 signaling. Cell Metab. *9*, 125–139.

Shapiro, A.M., Lakey, J.R., Ryan, E.A., Korbutt, G.S., Toth, E., Warnock, G.L., Kneteman, N.M., and Rajotte, R.V. (2000). Islet transplantation in seven patients with type 1 diabetes mellitus using a glucocorticoid-free immunosuppressive regimen. N. Engl. J. Med. *343*, 230–238.

Shapiro, A.M.J., Pokrywczynska, M., and Ricordi, C. (2017). Clinical pancreatic islet transplantation. Nat. Rev. Endocrinol. *13*, 268–277.

Shimada, A., Morimoto, J., Kodama, K., Suzuki, R., Oikawa, Y., Funae, O., Kasuga, A., Saruta, T., and Narumi, S. (2001). Elevated serum IP-10 levels observed in type 1 diabetes. Diabetes Care *24*, 510–515.

Shultz, L.D., Keck, J., Burzenski, L., Jangalwe, S., Vaidya, S., Greiner, D.L., and Brehm, M.A. (2019). Humanized mouse models of immunological diseases and precision medicine. Mamm. Genome *30*, 123–142.

Solomon, M., Flodström-Tullberg, M., and Sarvetnick, N. (2011). Beta-cell specific expression of suppressor of cytokine signaling-1 (SOCS-1) delays islet allograft rejection by down-regulating Interferon Regulatory Factor-1 (IRF-1) signaling. Transpl. Immunol. *24*, 181–188.

Stanley, W.J., Litwak, S.A., Quah, H.S., Tan, S.M., Kay, T.W.H., Tiganis, T., de Haan, J.B., Thomas, H.E., and Gurzov, E.N. (2015). Inactivation of protein tyrosine phosphatases enhances interferon signaling in pancreatic islets. Diabetes *64*, 2489–2496.

Toniato, E., Chen, X.P., Losman, J., Flati, V., Donahue, L., and Rothman, P. (2002). TRIM8/GERP RING finger protein interacts with SOCS-1. J. Biol. Chem. *277*, 37315–37322.

Tretina, K., Park, E.S., Maminska, A., and MacMicking, J.D. (2019). Interferon-induced guanylate-binding proteins: guardians of host defense in health and disease. J. Exp. Med. *216*, 482–500.

Uno, S., Imagawa, A., Saisho, K., Okita, K., Iwahashi, H., Hanafusa, T., and Shimomura, I. (2010). Expression of chemokines, CXC chemokine ligand 10 (CXCL10) and CXCR3 in the inflamed islets of patients with recent-onset autoimmune type 1 diabetes. Endocr. J. *57*, 991–996.

Veres, A., Faust, A.L., Bushnell, H.L., Engquist, E.N., Kenty, J.H.R., Harb, G., Poh, Y.C., Sintov, E., Gürtler, M., Pagliuca, F.W., et al. (2019). Charting cellular identity during human in vitro beta-cell differentiation. Nature *569*, 368–373.

Wang, D., Quan, Y., Yan, Q., Morales, J.E., and Wetsel, R.A. (2015). Targeted disruption of the beta2-microglobulin gene minimizes the immunogenicity of human embryonic stem cells. Stem Cells Transl. Med. *4*, 1234–1245.

Wei, Z., Yoshihara, E., He, N., Hah, N., Fan, W., Pinto, A.F.M., Huddy, T., Wang, Y., Ross, B., Estepa, G., et al. (2018). Vitamin D switches BAF complexes to protect beta cells. Cell *173*, 1135–1149.e15.

Wildbaum, G., Netzer, N., and Karin, N. (2002). Plasmid DNA encoding IFN-gamma-inducible protein 10 redirects antigen-specific T cell polarization and suppresses experimental autoimmune encephalomyelitis. J. Immunol. *168*, 5885–5892.

Xu, H., Wang, B., Ono, M., Kagita, A., Fujii, K., Sasakawa, N., Ueda, T., Gee, P., Nishikawa, M., Nomura, M., et al. (2019). Targeted disruption of HLA genes via CRISPR-cas9 generates iPSCs with enhanced immune compatibility. Cell Stem Cell *24*, 566–578.e7.

Yonekawa, K., and Harlan, J.M. (2005). Targeting leukocyte integrins in human diseases. J. Leukoc. Biol. *77*, 129–140.

Yoshihara, E., O'Connor, C., Gasser, E., Wei, Z., Oh, T.G., Tseng, T.W., Wang, D., Cayabyab, F., Dai, Y., Yu, R.T., et al. (2020). Immune-evasive human islet-like organoids ameliorate diabetes. Nature *586*, 606–611.

Yoshimatsu, G., Kunnathodi, F., Saravanan, P.B., Shahbazov, R., Chang, C., Darden, C.M., Zurawski, S., Boyuk, G., Kanak, M.A., Levy, M.F., et al. (2017). Pancreatic beta-cell-derived IP-10/CXCL10 isletokine mediates early loss of graft function in islet cell transplantation. Diabetes *66*, 2857–2867.

Stem Cell Reports, Volume *17*

Supplemental Information

Whole-genome CRISPR screening identifies genetic manipulations to reduce immune rejection of stem cell-derived islets

Elad Sintov, Igor Nikolskiy, Victor Barrera, Jennifer Hyoje-Ryu Kenty, Alexander S. Atkin, Dario Gerace, Shannan J. Ho Sui, Kyle Boulanger, and Douglas A. Melton

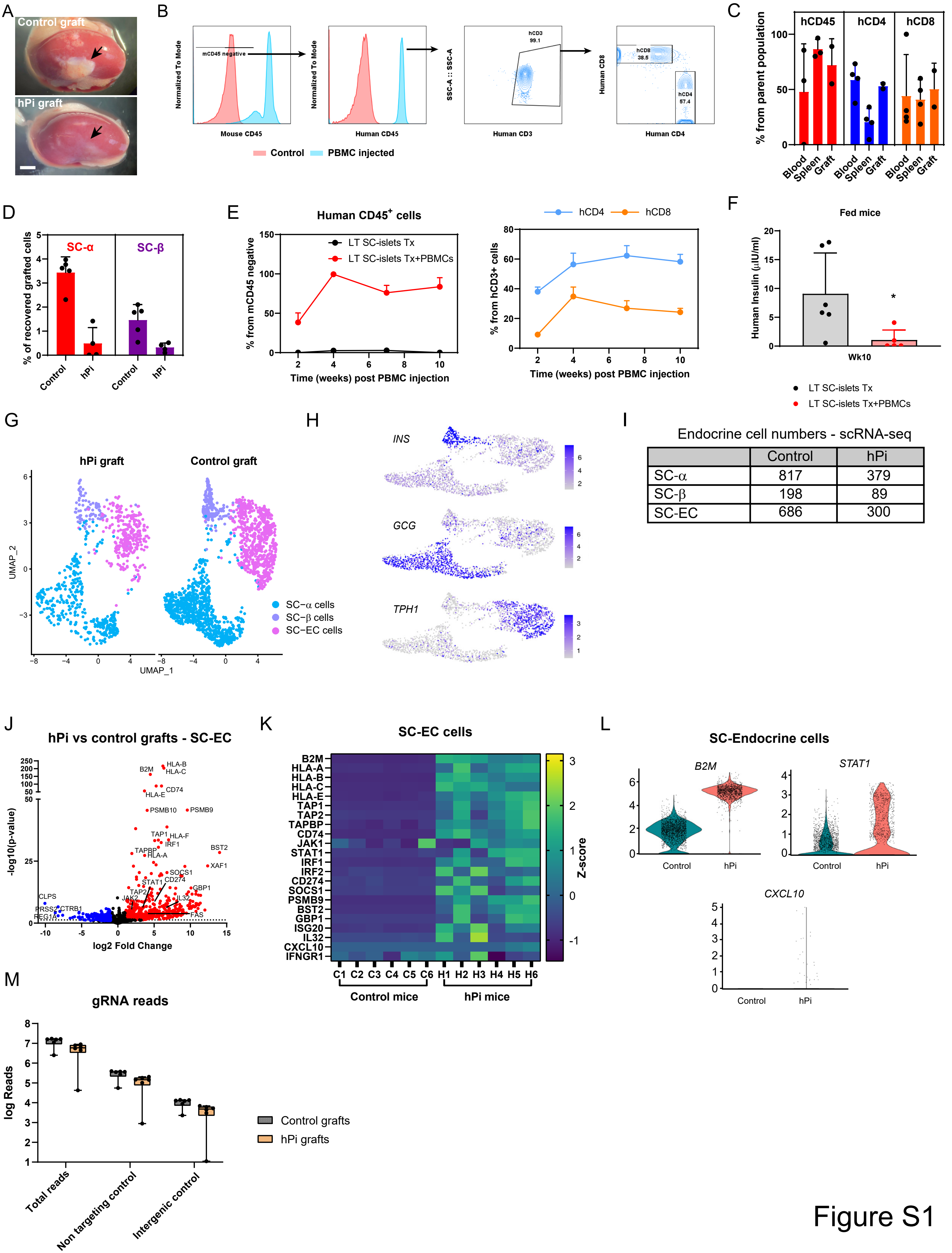
A
Control graft
hPi graft
B
Normalized To Mode
mCD45 negative
Mouse CD45
Human CD45
SSC-A :: SSC-A
hCD3
99.1
Human CD3
Human CD8
hCD8
38.5
hCD4
57.4
Human CD4
Control
PBMC injected
C
% from parent population
hCD45
hCD4
hCD8
Blood
Spleen
Graft
D
% of recovered grafted cells
SC-α
SC-β
Control
hPi
E
Human CD45+ cells
% from mCD45 negative
LT SC-islets Tx
LT SC-islets Tx+PBMCs
Time (weeks) post PBMC injection
hCD4
hCD8
% from hCD3+ cells
F
Fed mice
Human Insulin (µIU/ml)
Wk10
*
LT SC-islets Tx
LT SC-islets Tx+PBMCs
G
hPi graft
Control graft
UMAP_2
UMAP_1
SC−α cells
SC−β cells
SC−EC cells
H
INS
GCG
TPH1
I
Endocrine cell numbers - scRNA-seq
	Control	hPi
SC-α	817	379
SC-β	198	89
SC-EC	686	300
J
hPi vs control grafts - SC-EC
-log10(p-value)
log2 Fold Change
B2M
HLA-B
HLA-C
CD74
HLA-E
PSMB10
PSMB9
TAP1
HLA-F
IRF1
TAPBP
HLA-A
BST2
XAF1
SOCS1
STAT1
CD274
TAP2
JAK2
IL32
GBP1
FAS
CLPS
PRSS2
CTRB1
REG1A
K
SC-EC cells
B2M
HLA-A
HLA-B
HLA-C
HLA-E
TAP1
TAP2
TAPBP
CD74
JAK1
STAT1
IRF1
IRF2
CD274
SOCS1
PSMB9
BST2
GBP1
ISG20
IL32
CXCL10
IFNGR1
C1 C2 C3 C4 C5 C6 H1 H2 H3 H4 H5 H6
Control mice
hPi mice
Z-score
L
SC-Endocrine cells
B2M
STAT1
CXCL10
Control
hPi
M
gRNA reads
log Reads
Total reads
Non targeting control
Intergenic control
Control grafts
hPi grafts

Figure S1

Figure S1. Single cell transcriptional profile and whole genome CRISPR screen of SC-islet grafts in an *in vivo* humanized model. Related to Figure 1.

(A) Representative images of transplanted kidneys after 10 weeks with or without PBMC injection. Bar=2mm.

(B) Gating strategy used for flow cytometry of hPi/control mice blood to detect human T lymphocytes. %Human CD45+ are gated from mouse CD45 negative population. %CD4+ and %CD8+ are gated from hCD45+/hCD3+.

(C) Frequency of human T-lymphocytes in hPi mouse tissues, by flow cytometry.

(D) Frequency of SC-α (Glucagon+/C-peptide-) and SC-β (Glucagon-/C-peptide+) in recovered from SC-islet grafts, by flow cytometry.

(E) Flow cytometry of human T-lymphocytes in mice blood throughout the experiment, transplanted with library-transduced SC-islets (LT-SC-islets) ±PBMC injections (Control/hPi). %human CD45+ are gated from mouse CD45 negative population. %CD4+ and %CD8+ (only in hPi mice) are gated from hCD45+/hCD3+.

(F) Human Insulin detected by ELISA at the 10th week end point of the experiment, in non-fasted mice. Error bars are mean±SD. *p<0.05, **p<0.01, unpaired two-tailed t-test, LT SC-islets Tx+PBMCs (hPi) compared to the control.

(G) UMAP plots of human graft cells extracted from mice after 10 weeks with or without PBMC injection. Integration of n=6 mice per group. SC-endocrine cell clusters are indicated.

(H) Cluster identification (integration of n=12 mice) of specific SC-endocrine cells by gene markers (SC-β:*INS*+*GCG*-, SC-α: *INS*-*GCG*+, SC-EC: *TPH1*+).

(I) Cell counts of endocrine cell populations from scRNA-seq integrations.

(**J-L**) scRNA-seq analysis of SC-islet grafts. (**J**) Volcano plot of differential expressed genes in SC-EC in hPi vs. control grafts. **(K**) Differential expression of selected genes in SC-EC, presented as a heatmap. Each row specifies a z-score of the specified gene in all graft samples, in the indicated endocrine population. (**L**) Violin plots of selected genes, associated with the IFNγ response, expressed in SC-Endocrine cells.

(**M**) Total or control gRNA reads in CRISPR screen of mice replicates, compared between conditions (±PBMC; n=6 per condition). Box lines represent median values.

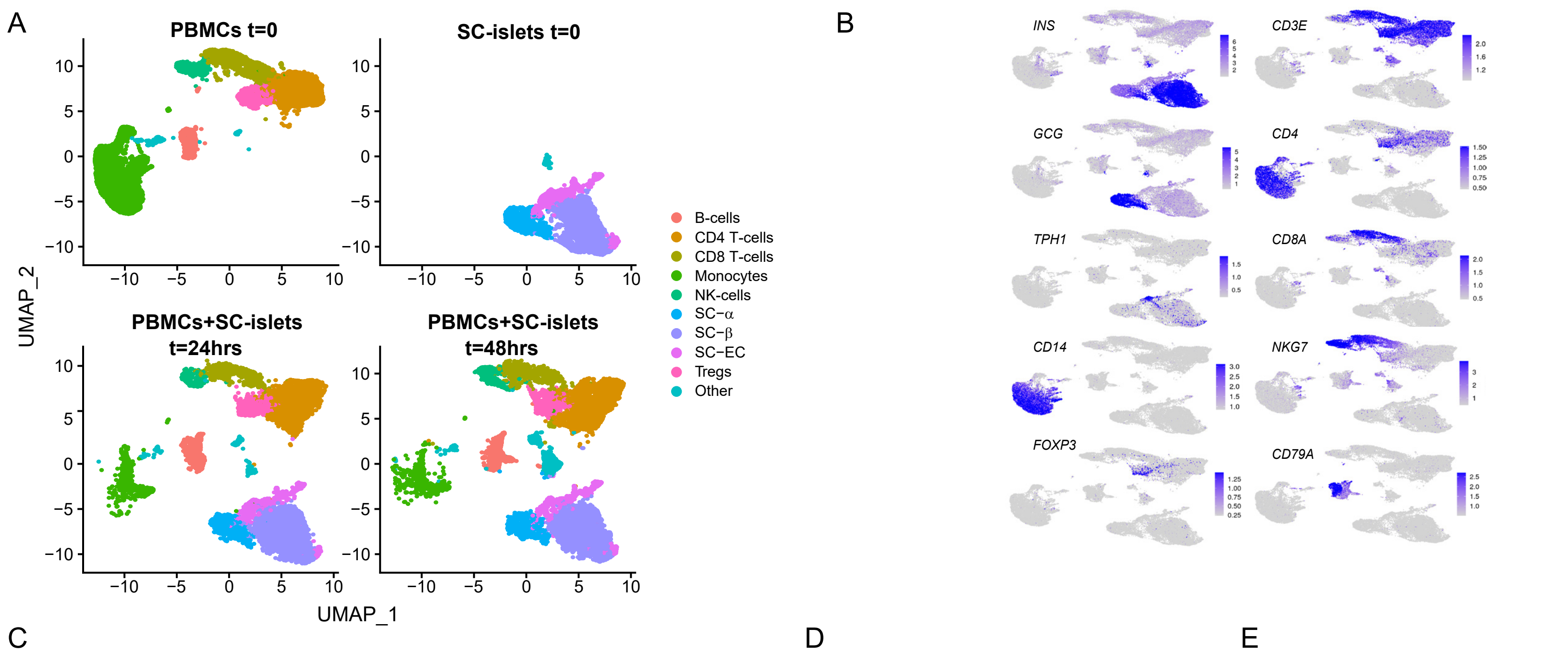

C

Cell numbers - scRNA-seq

	PBMC 0hr	SC-islet 0hr	CC 24hr	CC 48hr
CD4 T-cells	2737	0	2288	1977
CD8 T-cells	2391	0	477	866
B-cells	1140	0	729	859
Monocytes	7725	0	808	681
NK-cells	918	0	152	357
Treg	1217	0	711	654
SC-α	0	1618	877	593
SC-β	0	4723	2672	2006
SC-EC	0	875	573	468
Other	369	0	711	654

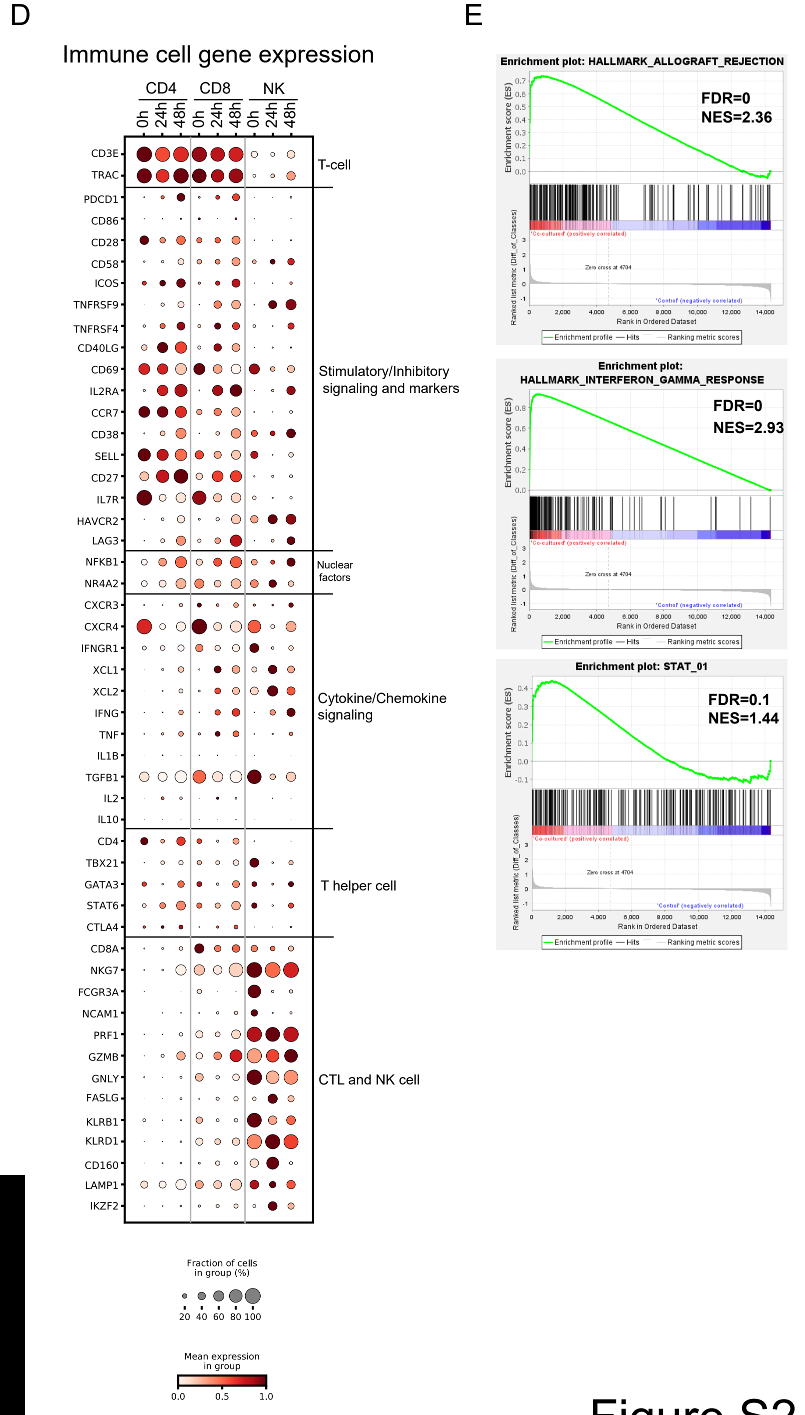

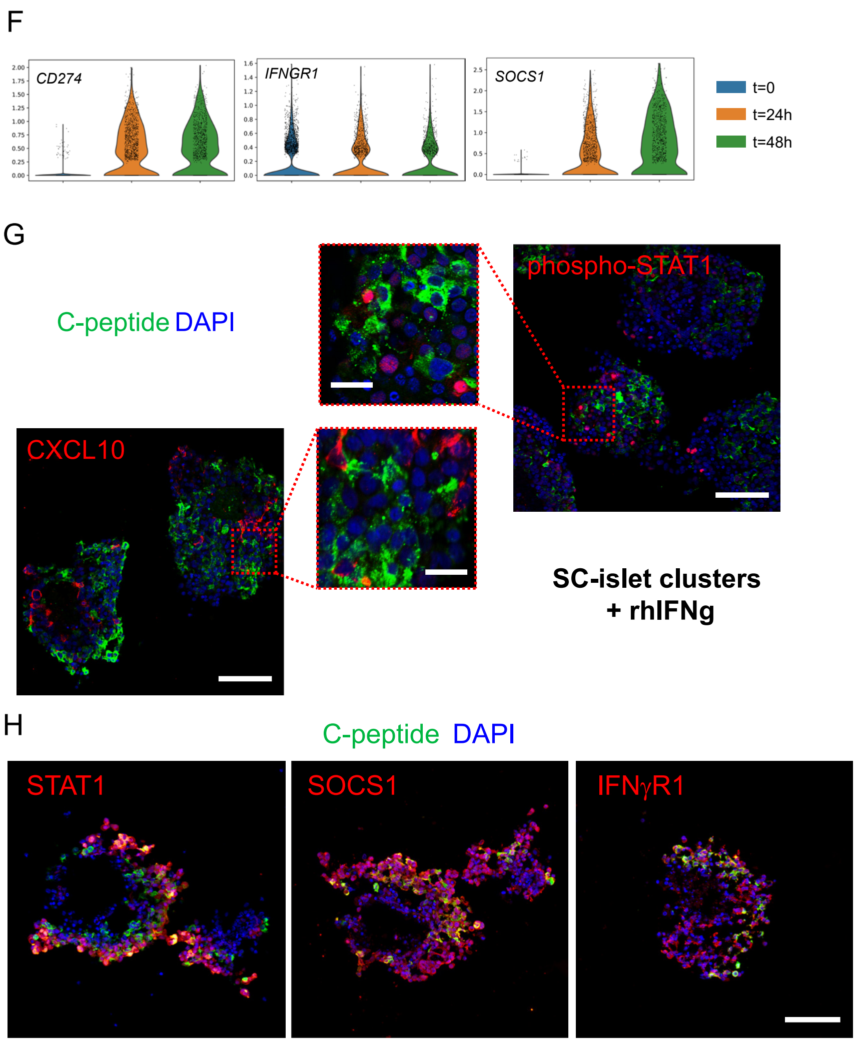

Figure S2

Figure S2. Early response of immune challenged SC-islets profiled by single cell transcription analysis after co-culture with human allogeneic PBMCs. Related to Figure 2.

(**A**) UMAP plots of PBMC+SC-islet co-cultured cells, immune/SC-islet cell clusters are indicated.

(**B**) Cluster identification (integration of all time points) of specific cells by gene markers.

(**C**) Cell counts of cell populations from scRNA-seq integrations. CC=co-culture.

(**D**) Dot plots representing expression of activation/inhibitory genes in specific immune populations, in response to timed SC-islet stimulation.

(**E**) Selected GSEA plots for interferon response and TF motifs, FDR values and normalized enrichment scores (NES) indicated.

(**F**) Violin plots of SC-β timed expression of selected genes.

(**G**) CXCL10 and phosphorylated STAT1 in SC-islet clusters 48hrs after treatment with 20ng/ml rhIFNγ. C-peptide staining for SC-β. Bars are 100μm in main panels and 20μm in magnified panels.

(**H**) IF staining of SC-islet clusters after 48hrs co-culture with PBMC. C-peptide staining (green) for SC-β and DAPI (blue) for nuclei. Bars are 100μm.

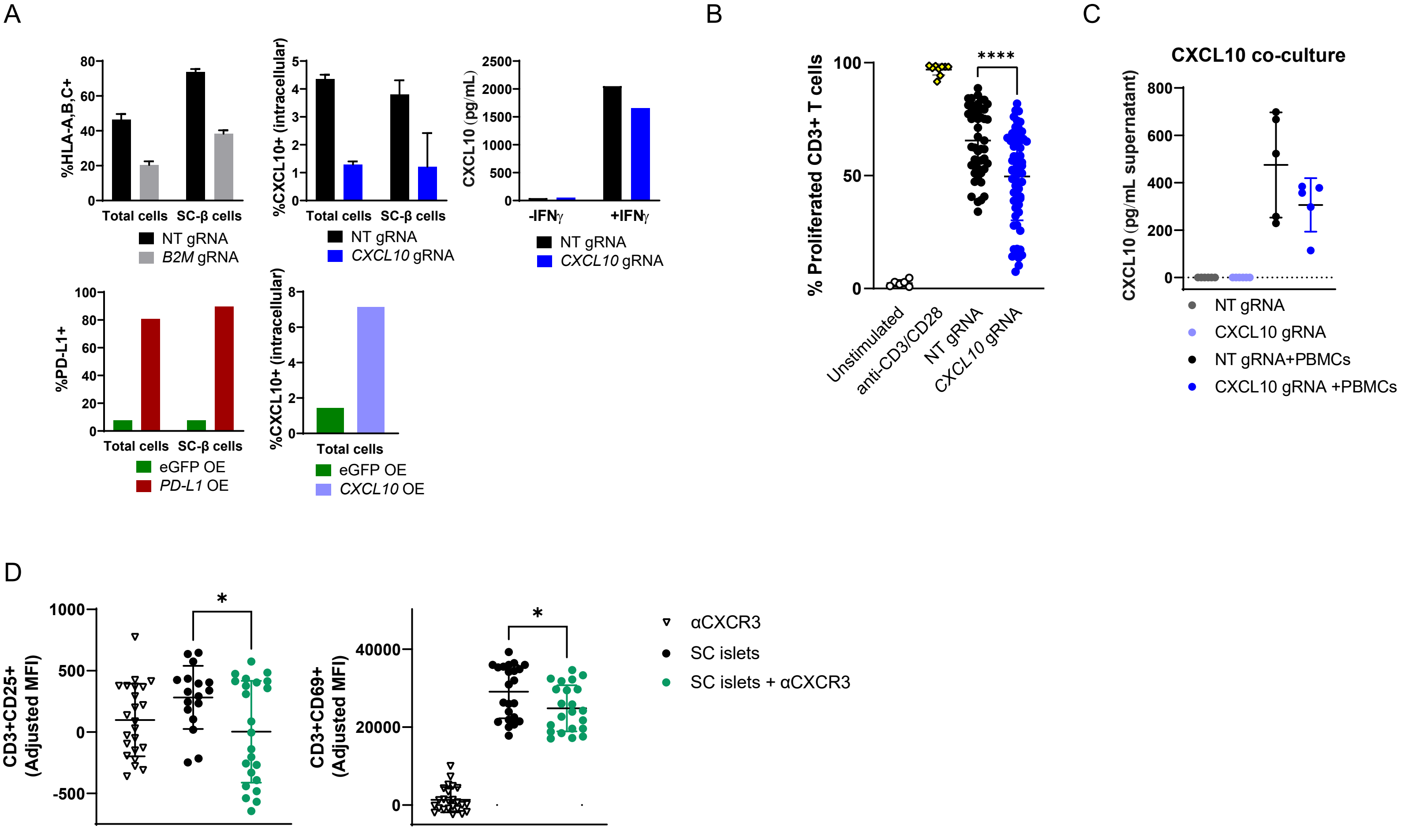
A
%HLA-A,B,C+
Total cells
SC-β cells
NT gRNA
B2M gRNA
%CXCL10+ (intracellular)
NT gRNA
CXCL10 gRNA
CXCL10 (pg/mL)
-IFNγ
+IFNγ
%PD-L1+
eGFP OE
PD-L1 OE
%CXCL10+ (intracellular)
CXCL10 OE
B
% Proliferated CD3+ T cells
****
Unstimulated
anti-CD3/CD28
NT gRNA
CXCL10 gRNA
C
CXCL10 co-culture
CXCL10 (pg/mL supernatant)
NT gRNA+PBMCs
CXCL10 gRNA +PBMCs
D
CD3+CD25+ (Adjusted MFI)
CD3+CD69+ (Adjusted MFI)
αCXCR3
SC islets
SC islets + αCXCR3

Figure S3

Figure S3. Immunogenicity of CXCL10 expressing SC-islets. Related to Figure 3.

(**A**) Flow cytometry analysis of protein expression of indicated perturbation in SC-islets or specifically in C-peptide+ SC-β 48hrs after rhIFNγ treatment. Where indicated CXCL10 secretion was measured by ELISA.

(**B**) Transduced SC-islets were co-cultured with Cell Trace Violet (CTV) labeled PBMC for 48hrs. PBMCs were then separated and allowed to grow in culture for an additional 7 days, followed by CD3 staining for flow cytometry. CD3+ were gated for the CTV negative fraction of divided cells. PBMCs treated with anti-CD3/CD28 activation beads served as positive control. n=12 for x5 PBMC donors (n=3 for controls).

C) ELISA for human CXCL10, from supernatant of co-culture of SC-islets transduced with NT/CXCL10 gRNA ±PBMCs, n=2-3 for x2 donors. Dashed line is the lower detection limit.

(**D**) Antibody treated (as indicated) SC-islets were co-cultured with PBMC for 48hrs. PBMCs were then separated and analyzed by flow cytometry for CD3+ T cell activation marker expression (CD25 or CD69). n=12 for x2 donors.

NT=Non-targeting, OE=overexpression. Error bars are mean±SD. ns=not significant, *p<0.05, **p<0.01, ***p<0.001, ****p<0.0001 unpaired two-tailed t-test.

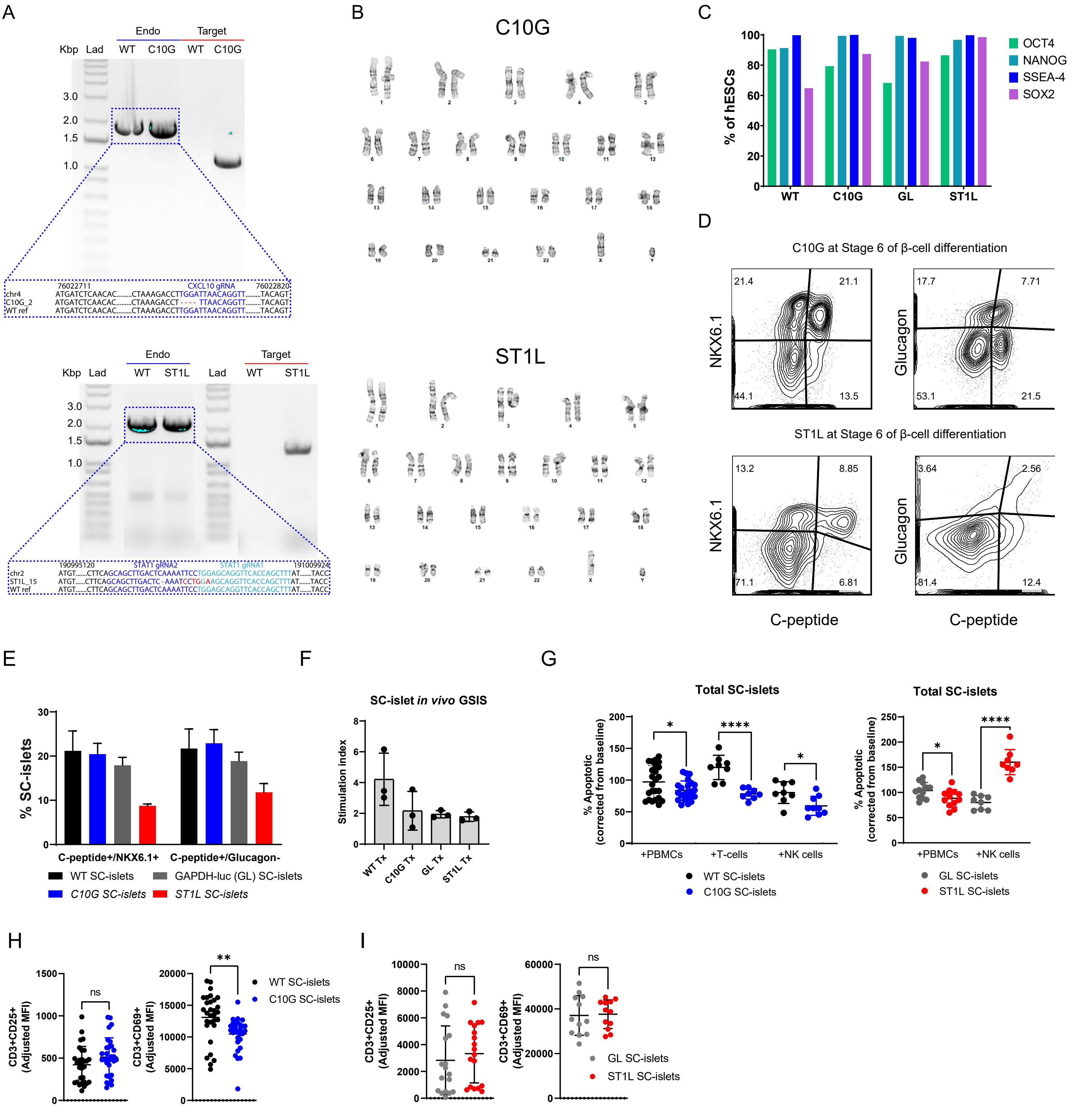
A
Endo
Target
Kbp
Lad
WT
C10G
WT
C10G
3.0
2.0
1.5
1.0
CXCL10 gRNA
chr4
C10G_2
WT ref
Endo
Target
Kbp
Lad
WT
ST1L
Lad
WT
ST1L
STAT1 gRNA2
STAT1 gRNA1
chr2
ST1L_15
WT ref
B
C10G
ST1L
C
% of hESCs
WT
C10G
GL
ST1L
OCT4
NANOG
SSEA-4
SOX2
D
C10G at Stage 6 of β-cell differentiation
ST1L at Stage 6 of β-cell differentiation
NKX6.1
Glucagon
C-peptide
E
% SC-islets
C-peptide+/NKX6.1+
C-peptide+/Glucagon-
WT SC-islets
GAPDH-luc (GL) SC-islets
C10G SC-islets
ST1L SC-islets
F
SC-islet in vivo GSIS
Stimulation index
WT Tx
C10G Tx
GL Tx
ST1L Tx
G
Total SC-islets
% Apoptotic (corrected from baseline)
+PBMCs
+T-cells
+NK cells
WT SC-islets
C10G SC-islets
GL SC-islets
ST1L SC-islets
H
CD3+CD25+ (Adjusted MFI)
CD3+CD69+ (Adjusted MFI)
ns
**
WT SC-islets
C10G SC-islets
I
ns
GL SC-islets
ST1L SC-islets

Figure S4

Figure S4. Generation and performance of *CXCL10* KO and *STAT1* KO hESC lines. Related to Figure 4.

(**A**) Clonal genotyping of endogenous or targeted allelles. Endogenous amplified PCR bands were isolated and sequenced for detection of indels, shown in dashed blue frames. Some lanes were cropped to show only relevant clones.

(**B**) Karyotyping analysis of G10G and ST1L hESC cell lines.

(**C**) Pluripotent marker expression by flow cytometry in all 4 lines: wild type (WT), C10G, GAPDH-luc (GL) and ST1L.

(**D**) Flow cytometry analysis to assess %SC-β (%C-peptide+/NKX6.1+ or %C-peptide+/glucagon) in C10G and ST1L hESC at stage 6 of the β-cell differentiation protocol.

(**E**) %SC-β in multiple batches of C10G and ST1L differentiations compared to control WT and GL lines. n=2-3 differentiations.

(**F)** SC-islet GSIS function assay of different lines, 12-15 weeks after transplantation into NSG-mice. Results presented as stimulation ratios of blood human insulin (ELISA) before and 30 min after glucose injection (2g/kg).

(**G**) Flow cytometry was used to assess %TUNEL+ SC-islets. Apoptosis was calculated by fraction from baseline (%TUNEL without PBMC). Left) WT or C10G SC-β cells (n=4 for x6 PBMC donors, n=2-3 x2 T-cell donors, n=4 x2 NK cell donors); Right) GL vs. ST1L SC-β cells (n=4 for x2 PBMC or NK cell donors).

(**H,I**) GM SC-islets were co-cultured with PBMC for 48hrs. PBMCs were then separated and analyzed by flow cytometry for CD3+ T cell activation marker expression (CD25 or CD69). (**H**) n=9 for x3 PBMC donors (**I**) n=9 for x2 PBMC donors

Error bars are mean±SD. ns=not significant, *p<0.05, **p<0.01, ****p<0.0001 unpaired two-tailed t-test.

A

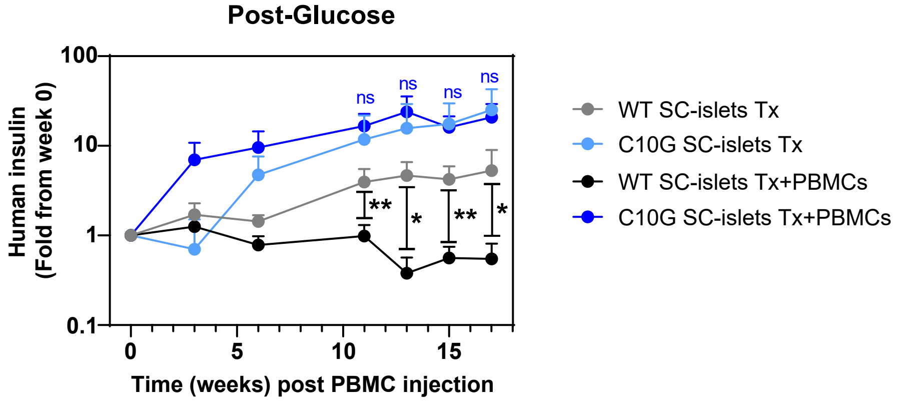
Post-Glucose
Human insulin (Fold from week 0)
100
10
1
0.1
0
5
10
15
Time (weeks) post PBMC injection
ns
ns
ns
ns
**
*
**
*
WT SC-islets Tx
C10G SC-islets Tx
WT SC-islets Tx+PBMCs
C10G SC-islets Tx+PBMCs

B

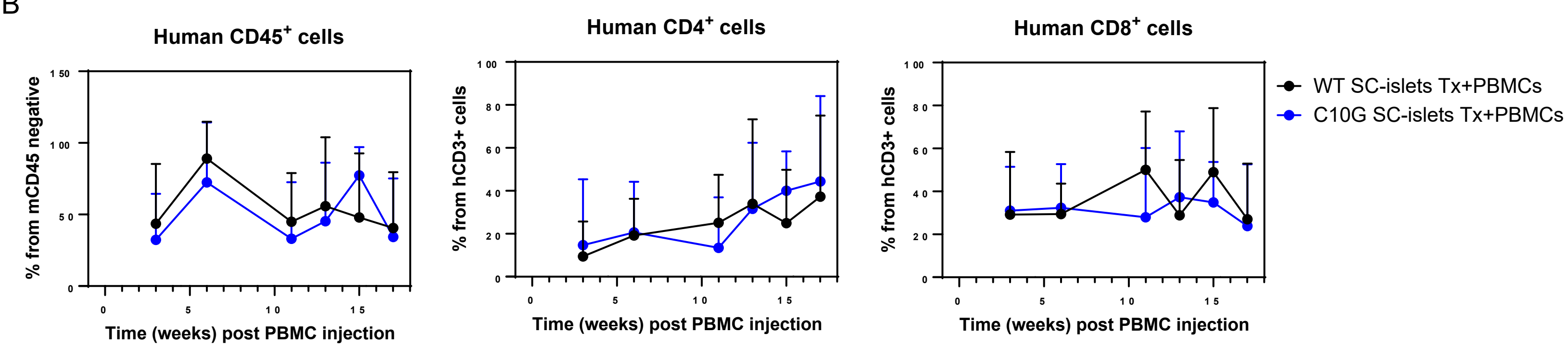
Human CD45+ cells
% from mCD45 negative
Time (weeks) post PBMC injection
Human CD4+ cells
% from hCD3+ cells
Time (weeks) post PBMC injection
Human CD8+ cells
% from hCD3+ cells
Time (weeks) post PBMC injection
WT SC-islets Tx+PBMCs
C10G SC-islets Tx+PBMCs

Figure S5

Figure S5. ***CXCL10*** **KO SC-islet transplantation in humanized mice. Related to Figure 5.**

(**A**) Graft failure continuously monitored over time after PBMC injections, as measured by human insulin (ELISA) in fasted mice plasma, 30 min after glucose injection to fasted mice. Data presented as fold increase from t=0 before PBMC injections.

(**B**) Flow cytometry of human T-lymphocytes in mice blood throughout the experiment, transplanted WT/C10G SC-islets +PBMC injections. %human CD45+ are gated from mouse CD45 negative population. %CD4+ and %CD8+ (only in hPi mice) are gated from hCD45+/hCD3+.

Error bars are mean±SD. ns=not significant, *p<0.05, **p<0.01, unpaired two-tailed t-test.

SUPPLEMENTARY TABLES

Table S1 - Panther pathway analysis of upregulated genes in a given cell population. Related to results presented in Figures 1 and 2.

	Term	Enrichment Ratio	False discovery rate (FDR)
**SC-α in vivo SCRNA-seq**	JAK/STAT signaling pathway	5.419432	7.69E-04
	FAS signaling pathway	2.950094	0.026945
	Apoptosis signaling pathway	2.916708	1.20E-06
	T cell activation	2.845202	2.69E-04
	Toll receptor signaling pathway	2.438744	0.028295
	Inflammation mediated by chemokine and cytokine signaling pathway	1.879865	0.00133
**SC-β in vivo SCRNA-seq**	JAK/STAT signaling pathway	15.10667	0.09678
	Oxidative stress response	6.568116	0.156495
	T cell activation	4.028444	0.594957
	PDGF signaling pathway	3.021333	0.623899
	Ras Pathway	3.237143	1
	Toll receptor signaling pathway	3.021333	1
**SC-EC in vivo SCRNA-seq**	Interferon alpha/beta signaling	14.13884	0
	Antigen Presentation: Folding, assembly and peptide loading of class I MHC	13.00773	3.19E-08
	Interferon gamma signaling	11.2468	0.00E+00
	Cytokine Signaling in Immune system	4.468829	0
	Adaptive Immune System	3.089258	0.00E+00
	Immune System	2.916332	0.00E+00
**SC-α in vitro SCRNA-seq**	T cell activation	6.972308	0.501353
	Apoptosis signaling pathway	4.84188	0.599738
	Interleukin signaling pathway	4.469428	1
	JAK/STAT signaling pathway	11.62051	1
	Interferon-gamma signaling pathway	6.45584	1
	Toll receptor signaling pathway	3.486154	1
**SC-β in vitro SCRNA-seq**	JAK/STAT signaling pathway	19.70435	0.043412
	Oxidative stress response	6.425331	0.589624
	Interferon-gamma signaling pathway	7.297907	0.85533
	T cell activation	3.94087	0.85533
	Apoptosis signaling pathway	2.736715	1
	Inflammation mediated by chemokine and cytokine signaling pathway	1.970435	1
**CD4 T cells in vitro SCRNA-seq**	T cell activation	4.028444	0.071289
	Inflammation mediated by chemokine and cytokine signaling pathway	2.454833	0.08497
	Cytoskeletal regulation by Rho GTPase	3.723474	0.08497
	PDGF signaling pathway	2.7192	0.134924
	JAK/STAT signaling pathway	7.553333	0.144705
	Integrin signalling pathway	2.2751	0.153289

**CD8 T cells in vitro SCRNA-seq**	Inflammation mediated by chemokine and cytokine signaling pathway	4.532	3.80E-04
	Apoptosis signaling pathway	4.196296	0.134094
	Integrin signalling pathway	3.185141	0.180917
	T cell activation	4.028444	0.446218
	Axon guidance mediated by Slit/Robo	8.392593	0.508594
	Toll receptor signaling pathway	4.532	0.508594
**NK cells in vitro SCRNA-seq**	Apoptosis signaling pathway	3.38411	4.47E-02
	T cell activation	3.898495	0.04465
	Inflammation mediated by chemokine and cytokine signaling pathway	2.375645	0.063865
	JAK/STAT signaling pathway	7.309677	0.158653
	Integrin signalling pathway	2.20171	0.183223
	FAS signaling pathway	3.536941	0.575141

Table S2 - Reactome pathway analysis of upregulated genes in a given cell population. Related to results presented in Figures 1 and 2.

	**Term**	**Enrichment Ratio**	**FDR**
**SC-α in vivo SCRNA-seq**	Interferon alpha/beta signaling	7.109533	0.00E+00
	Interferon gamma signaling	6.246232	0.00E+00
	Interferon Signaling	4.33996	0
	Programmed Cell Death	3.341008	6.27E-10
	Apoptosis	3.151512	2.09E-08
	Cytokine Signaling in Immune system	2.892824	0
**SC-β in vivo SCRNA-seq**	Interferon alpha/beta signaling	26.14641	0
	Interferon gamma signaling	19.60981	0
	Antigen processing-Cross presentation	14.57861	2.75E-12
	Interferon Signaling	13.73682	0
	Cytokine Signaling in Immune system	5.637821	0
	Immune System	3.252263	0
**SC-EC in vivo SCRNA-seq**	Interferon alpha/beta signaling	14.13884	0
	Antigen Presentation: Folding, assembly and peptide loading of class I MHC	13.00773	3.19E-08
	Interferon gamma signaling	11.2468	0.00E+00
	Cytokine Signaling in Immune system	4.468829	0
	Adaptive Immune System	3.089258	0.00E+00
	Immune System	2.916332	0.00E+00
**SC-α in vitro SCRNA-seq**	Cytokine Signaling in Immune system	10.06695	0
	Antigen processing-Cross presentation	24.9858	0
	Interferon gamma signaling	28.67935	0
	Interferon alpha/beta signaling	45.40897	0
	Antigen Presentation: Folding, assembly and peptide loading of class I MHC	52.77	2.51E-10
	Class I MHC mediated antigen processing & presentation	8.000842	7.03E-10
**SC-β in vitro**	Cytokine Signaling in Immune system	7.912271	0
	Interferon gamma signaling	20.52838	0

**SCRNA-seq**	Interferon alpha/beta signaling	37.03158	0
	Adaptive Immune System	3.673768	2.10E-06
	Antigen processing-Cross presentation	11.22169	4.29E-06
	Signaling by Interleukins	4.328366	2.35E-05
**CD4 T cells in vitro SCRNA-seq**	Axon guidance	4.14862	0
	rRNA processing in the nucleus and cytosol	7.731868	0
	Major pathway of rRNA processing in the nucleolus and cytosol	8.149807	0
	Regulation of expression of SLITs and ROBOs	9.385884	0
	Eukaryotic Translation Initiation	13.07857	0
	Formation of a pool of free 40S subunits	14.92786	0
**CD8 T cells in vitro SCRNA-seq**	Innate Immune System	3.233159	2.43E-06
	Signaling by Interleukins	3.684541	0.004806
	Cytokine Signaling in Immune system	2.804107	0.031589
	Immunoregulatory interactions between a Lymphoid and a non-Lymphoid cell	6.018084	0.043057
	CD28 co-stimulation	13.75562	0.043057
	Cell surface interactions at the vascular wall	5.798446	0.043057
**NK cells in vitro SCRNA-seq**	Immunoregulatory interactions between a Lymphoid and a non-Lymphoid cell	7.902484	3.34E-08
	Cytokine Signaling in Immune system	3.210722	1.60E-07
	Innate Immune System	2.505698	3.71E-06
	Signaling by Interleukins	3.453186	9.57E-06
	Adaptive Immune System	2.759598	1.28E-05
	Interferon Signaling	3.737693	0.015391

Table S3 – Gene Ontology (GO) terms of biological processes of upregulated genes in a given cell population. Related to results presented in Figures 1 and 2.

	**Term**	**Enrichment Ratio**	**FDR**
**SC-α in vivo SCRNA-seq**	negative regulation of immune system process	3.550315	0
	activation of immune response	3.260719	0
	immune effector process	3.242858	0
	leukocyte activation	3.059432	0
	regulation of immune system process	2.865024	0
	leukocyte mediated immunity	2.802487	0
**SC-β in vivo SCRNA-seq**	response to type I interferon	23.08666	0
	cellular response to interferon-gamma	13.937	0
	cytokine-mediated signaling pathway	7.124298	0
	cellular response to cytokine stimulus	6.297969	0
	defense response	4.812677	0
	immune response	4.639788	0
**SC-EC in vivo SCRNA-seq**	negative regulation of immune system process	4.736849	0
	activation of immune response	4.37812	0
	immune effector process	4.06874	0
	leukocyte activation	3.892765	0
	regulation of immune system process	3.769293	0

	leukocyte mediated immunity	3.251988	0
**SC-α in vitro SCRNA-seq**	immune response	6.221897	0
	defense response	7.70163	0
	response to cytokine	9.497585	0
	innate immune response	10.52735	0
	defense response to other organism	12.09548	0
	response to interferon-gamma	22.02441	0
**SC-β in vitro SCRNA-seq**	immune response	5.299158	0
	defense response	6.792044	0
	immune effector process	5.322702	0
	response to cytokine	7.575455	0
	innate immune response	9.393048	0
	response to virus	15.0535	0
**CD4 T cells in vitro SCRNA-seq**	immune response	2.924152	0
	regulation of immune system process	3.126378	0
	cell activation	3.026399	0
	leukocyte activation	3.222791	0
	establishment of protein localization to organelle	4.911323	0
	protein targeting	5.584208	0
**CD8 T cells in vitro SCRNA-seq**	immune response	3.501908	2.02E-12
	leukocyte activation	4.200101	7.00E-11
	cell motility	3.541587	6.17E-10
	response to cytokine	4.154282	6.21E-10
	cell migration	3.635175	1.93E-09
	regulation of immune response	4.43575	2.74E-09
**NK cells in vitro SCRNA-seq**	cell activation	3.159824	4.04E-13
	regulation of immune response	3.803824	4.04E-13
	response to cytokine	3.39481	1.51E-12
	cellular response to cytokine stimulus	3.474709	3.75E-12
	cytokine-mediated signaling pathway	3.923604	1.08E-10
	lymphocyte activation	3.960806	6.91E-10

Supplemental Experimental Procedures

- **KEY RESOURCES TABLE**
- **CONTACT FOR REAGENT AND RESOURCE SHARING**
- **EXPERIMENTAL MODEL AND SUBJECT DETAILS**
- **METHOD DETAILS**
    - **hESC cell culture and differentiation**
    - **Cell transplantation**
    - **Secretion assays**
    - **In vivo bioluminescence imaging**
    - **Flow cytometry**
    - **Immunofluorescence Microscopy**

- **Magnetic enrichment using CD49a**
- **Human primary immune cell isolation and co-culture assays**
- **Tissue/cell preparation and library preperation for single cell RNA sequencing**
- **In vivo single cell RNA sequencing analysis**
- **In vitro single cell RNA sequencing analysis**
- **Lentivirus Preparation and Transduction**
- **Whole genome CRISPR screen in vivo and analysis**
- **Generation of hESC knockout lines**

- **QUANTIFICATION AND STATISTICAL ANALYSIS**
- **GRAPHIC ILLUSTRATIONS**
- **DATA AVAILABILITY**

KEY RESOURCES TABLE

REAGENT or RESOURCE	SOURCE	IDENTIFIER
**Antibodies**		
Rat anti-C-peptide	Developmental Studies Hybridoma Bank (DHSB)	GN-ID4, RRID: AB_2255626
Mouse anti-NKX6.1	DHSB	F55A12, RRID: AB_532379
Goat anti-NANOG	R&D Systems	AF1997
Goat anti-SOCS1	Life Technologies	Cat#PA517938
Mouse anti-glucagon Alexa 647	Santa Cruz Biotech	Cat#SC-514592
Rabbit anti-STAT1	abcam	Cat#ab2415
Mouse anti-CD8	BD Biosciences	HIT8a, Cat#550372, RRID: AB_393643
Mouse anti- NKX6.1 PE-conjugated	BD Biosciences	Cat#563023
Mouse anti-pSTAT1 Alexa 647-conjugated	Biolegend	Cat#666410
Mouse anti-SOCS1 Alexa a88-conjugated	Santa Cruz Biotech	Cat#SC-518028
Mouse anti-IP10 PE-conjugated	Biolegend	Cat#519504
Mouse Anti-HLA-ABC Alexa 647-conjugated	Biolegend	W6/32, Cat#311414
Mouse Anti-HLA-ABC PE-conjugated	Biolegend	W6/32, Cat#311406
Mouse anti-HLA-E PE-conjugated	BD Biosciences	Cat#12995342
Mouse anti-CD119 PE-conjugated	Biolegend	GIR-94, Cat#308704
Rat anti CD16/CD32 (Fc Block)	BD Biosciences	Cat# 553141, RRID: AB_394656
Rat anti murine CD45 Alexa 647-conjugated	Biolegend	Cat#03124

Mouse anti-CD45 Alexa a88-conjugated	Biolegend	Cat#304017
Mouse anti-CD3 APC-conjugated	Biolegend	UCHT1, Cat#300412
Mouse anti-CD3 PE-conjugated	Biolegend	UCHT1, Cat#300408
Mouse anti-PD-L1 APC-conjugated	Biolegend	29E.2A3, Cat#329708
Mouse anti-CD3 PB-conjugated	Biolegend	UCHT1, Cat#300417
Mouse anti-CD8 PE-conjugated	Biolegend	T8-Leu2, Cat#344706
Mouse anti-CD4 PE/Cy7-conjugated	Biolegend	RPA-T4, Cat#300512
Mouse anti-CD69 Alexa 647-conjugated	Biolegend	FN50, Cat#310918
Mouse anti-CD25 Alexa 700-conjugated	Biolegend	M-A251, Cat#356118
Mouse anti-CD107a FITC-conjugated	ThermoFisher Scientific	H4A3, Cat# BDB555800
Mouse anti-CD56 APC-conjugated	Biolegend	Cat#362504
Mouse anti-CD49a PE-conjugated	BD Biosciences	Cat#559596
Mouse anti-Oct4 Alexa 647-conjugated	BD Pharmingen	Cat#653710
Mouse anti-Sox2 PE-conjugated	BD Pharmingen	Cat#560291
Mouse anti-SSEA4 V450-conjugated	BD Pharmingen	Cat#561156
Mouse IgG1 PB-conjugated	Biolegend	Cat#400131
Mouse IgG1 Alexa 488-conjugated	Biolegend	Cat#400132
Mouse IgG3, κ V450- conjugated	BD Biosciences	Cat#561600
Mouse IgG1 PE-conjugated	Biolegend	Cat#400114
Mouse IgG2b, κ PE-conjugated	Biolegend	Cat#400314
Mouse IgG2a, κ PE-conjugated	Biolegend	Cat#400214
Mouse IgG2a, κ Alexa 647-conjugated	Biolegend	Cat#400234
Mouse IgG2b, κ Alexa 647-conjugated	Biolegend	Cat#400330
Mouse IgG1 Alexa 647-conjugated	Biolegend	Cat#400136
Mouse IgG2a, κ APC-conjugated	BD Biosciences	Cat#17472442
Mouse IgG2b, κ APC-conjugated	Biolegend	Cat#400322
Mouse IgG2b, κ Alexa 700-conjugated	Biolegend	Cat#400334
Mouse IgG1 Alexa PE/Cy7-conjugated	Biolegend	Cat#400126
Donkey anti-mouse Alexa 647	Life Technologies	Cat#A31571
Donkey anti-rabbit Alexa 488	Life Technologies	Cat#A21206
Donkey anti-rabbit Alexa 594	Life Technologies	Cat#A21209
Donkey anti-goat Alexa 647	Life Technologies	Cat#A21447
Donkey anti-rat 488	Life Technologies	Cat# A21208
**Virus vectors**		
lentiCRISPRv2	Broad institute	RRID: Addgene_52961
pLX_307	Broad institute	RRID: Addgene_41392

pHDM-vsvG, -tat, rev, gag/pol	Harvard Medical School DNA Resource Core	N/A
**Biological Samples**		
Human Peripheral Blood apheresis collars	Kraft Family Blood Donor Center, Brigham and Woman's Hospital	N/A
**Oligonucleotides**		
Non targeting gRNA TTTACGATCTAGCGGCGTAG	This paper	N/A
B2M gRNA GCTACTCTCTCTTTCTGGCC	This paper	N/A
CXCL10 gRNA (for virus and HDR) GTAATCAACCTGTTAATCCA	Virus: eSpCas9-LentiCRISPR v2 by Genescript<br>HDR: IDT (Alt-R)	N/A
STAT1 gRNA (for virus) TGCTGGCACCAGAACGAATG	eSpCas9-LentiCRISPR v2 by Genescript	N/A
STAT1 gRNA#1 (for HDR) Alt-R AAAGCTGGTGAACCTGCTCC	Alt-R IDT	N/A
STAT1 gRNA#2 (for HDR) Alt-R GCAGCTTGACTCAAAATTCC	Alt-R IDT	N/A
IFNGR1 gRNA	eSpCas9-LentiCRISPR v2 by Genescript	N/A
CXCL10 Endo Forward ATCATTGGTCACCTTTTAGTGT	IDT	N/A
CXCL10 Endo Reverse ATAATACCTTCGAGTCTGCAAC	IDT	N/A
CXCL10 Edited Reverse TATAGATCTCTCGTGGGATCAT	IDT	N/A
CXCL10 gRNA cut site Forward TTCTGGATTCAGACATCTCTTC	IDT	N/A
CXCL10 gRNA cut site Reverse TTTGCTAAGTCAACTGTAATGC	IDT	N/A
STAT1 Endo Forward AAGATTCACTTGTGTCTGCTCT	IDT	N/A
STAT1 Endo Reverse TATATTGATCATCCAGCTGTGA	IDT	N/A

STAT1 Edited Forward AGATAAATGCCTGCTCTTTACT	IDT	N/A
STAT1 gRNA cut site Forward GGTCAGATGGTGGTGTAAGTAC	IDT	N/A
STAT1 gRNA cut site Reverse CCCTTCACTTTCTATGTCAAAT	IDT	N/A
**Chemicals, Peptides, and Recombinant Proteins**		
Activin A	R&D Systems	Cat#338-AC
Rock Inhibitor Y-27632	DNSK	Cat#DNSK-KI15-02
Chir99021	Stemgent	Cat#04-0004-10
KGF	Peprotech	Cat#100-19
Retinoic acid	Sigma-Aldrich	Cat# R2625
LDN193189	Sigma-Aldrich	Cat#SML0559
Sant1	Sigma-Aldrich	Cat#S4572
PBDU	EMD Millipore	Cat#524390
XXI	EMD Millipore	Cat#565790
Alk5i II	Axxora	Cat#ALX-270-445
T3	EMD Millipore	Cat#642511
Betacellulin	ThermoFisher Scientific	Cat# 565790
Human IFN gamma	R&D Systems	Cat#285IF
Human IL-2	Peprotech	Cat#200-02
Thapsigargin	Sigma-Aldrich	Cat#T9033
**Critical Commercial Assays**		
Chromium Next GEM Single Cell 3' Kit v3.1, 16 rxns	10X Genomics	PN-1000268
Chromium Next GEM Chip G Single Cell Kit, 48 rxns	10X Genomics	PN-1000120
Dual Index Kit TT Set A, 96 rxns	10X Genomics	PN-1000215
Kapa qPCR Library Quantification Kit, Complete Universal Kit	Roche Sequencing Solutions	Cat#07960140001
High Sensitivity D5000 Tape	Agilent Technologies	5067-5592
High Sensitivity D5000 Reagents |	Agilent Technologies	5067-5593
**Experimental Models: Cell Lines**		
Human ESC line Hues8	HSCI	HVRDe008-A
Human ESC line Hues8 GAPDH-luciferase (GL)	HSCI	HVRDe008-A-1
Lenti-X™ 293T Cell Line	Takara Bio	Cat#632180

Software and Algorithms		
GSEA 4.1.0	UC San Diego and Broad Institute	N/A
FlowJo v10.8.1	BD (Becton, Dickinson and Company)	N/A
GraphPad Prism9	GraphPad Software	N/A
R v4.0.3	R	N/A
Seurat suite v4.0.6	Hao et al., 2021	N/A
Scanpy v1.8.1	Wolf et al., 2018	N/A

CONTACT FOR REAGENT AND RESOURCE SHARING

Further information and requests for resources and reagents should be directed to and will be fulfilled by the Lead Contact, Douglas A. Melton (dmelton@harvard.edu).

EXPERIMENTAL MODEL AND SUBJECT DETAILS

All procedures were performed in accordance with the Institutional Review Board (IRB) guidelines at Harvard University under IRB and Embryonic Stem Cell Research Oversight Committee (ESCRO) Protocols E00024. All animal experiments were performed in accordance with Harvard University International Animal Care and Use Committee regulations.

METHOD DETAILS

hESC cell culture and differentiation

Human embryonic stem-cell (hESC) Hues8 maintenance and differentiation was carried out as previously described (Pagliuca et al., 2014). Induced pluripotent stem-cell lines were obtained from stocks maintained by the Melton laboratory. hESC line was maintained in cluster suspension culture format using mTeSR-1 (Stem Cell Technologies, 85850) in 500-ml spinner flasks (Corning, VWR) spinning at 70 r.p.m. in an incubator at 37 °C, 5% CO2 and 100% humidity. Cells were passaged every 72h or 96h: induced human pluripotent stem-cell clusters were dissociated to single cells using gentle cell dissociation reagent (Stem Cell Technologies; 07174) and light mechanical disruption, counted and seeded at 0.6 M cells/ml in mTeSR-1 + 10 µM Y27632 (ROCK inhibitor). Cell lines were authenticated by DNA fingerprinting, karyotyping (Cell Line Genetics) and all lines tested negative on routine mycoplasma contamination verifications. Differentiation flasks were started 72 h after passaging, by replacing mTeSR-1 medium with the appropriate differentiation medium including growth factors and small molecule supplements as previously described (Veres et al., 2019):

SC-β cells protocol

Stage 1: 24 hours in S1 medium supplemented with Activin A (100ng/ml), CHIR99021 (1.4μg/ml) and Rock Inhibitor (10μM), followed by 48 hours Activin A (100ng/ml) only.

Stage 2: 72 hours in S2 medium supplemented with KGF (50ng/ml) and Rock Inhibitor (10μM).

Stage 3: 48 hours in S3 medium supplemented with KGF (50ng/ml), LDN193189 (200nM), Sant1 (0.25μM), retinoic acid (2μM), PBDU (500nM) and Rock Inhibitor (10μM).

Stage 4: 5 days in S3 medium supplemented with KGF (50ng/ml), Sant1 (0.25μM), retinoic acid (0.1μM) and Rock Inhibitor (10μM).

Stage 5: 7 days in BE5 medium supplemented with Betacellulin (20ng/ml), XXI (1μM), Alk5i-II (10 μM) and T3 (1μM). Sant1 (0.25μM) was added in the first three days, and retinoic acid was added at 0.1μM in the first three days, then at 0.025μM.

Stage 6: 14-21 days in S3 medium, changed every 48 hours.

During feeds, the differentiating clusters were allowed to gravity-settle for 5–10 min, medium was aspirated, and 300 ml of pre-warmed medium was added. All experiments involving human cells were approved by the Harvard University IRB and ESCRO committees.

Cell transplantation

Pre-surgery animals were housed in groups within sterile cages with unrestricted access to food and water. Ambient temperature was maintained between 18 and 25 °C, humidity 30−70% with 12 h light/dark cycles. All animal research was conducted under Harvard IACUC approval. Transplantation of cell clusters was performed as previously described (Pagliuca et al., 2014). Briefly, 5M cells were injected under the kidney capsule of male NSG-(Kb Db)^{null} (IA^{null}) (DKO) (Jackson Labs; 025216) >8 wk old mice. Post-surgery, mice were single housed and monitored for up to 18 weeks after transplantation. For allograft rejections assays, 50M of human primary peripheral mononuclear cells (PBMCs) were injected intraperitoneally.

Kidney grafts were harvested, processed for sequencing or stained as described below.

For *in vivo* glucose stimulated insulin secretion (GSIS) and graft function monitoring, Human insulin and C-peptide were quantified from mouse blood plasma collected from the facial vein at fasted (overnight for 16h) and 30 min post-injection of glucose at 2g/kg bodyweight.

Secretion assays

Mouse plasma was used to measure human insulin or human C-peptide by ELISA (ALPCO Diagnostics, 80-INSHUU-E10 and 80-CPTHU-E10 respectively) as described in the manufacturer’s protocol. Supernatant from treated cell cultures was used to measure human CXCL10 by ELISA (BioLegend, 439904) as described in the manufacturer’s protocol.

Flow cytometry (FC)

All stained cells were analyzed using the Attune NxT (Thermo Fischer) flow cytometer. Data analysis was performed with FlowJo (BD) software. For IFN induced protein detection, SC-islets were treated with 20ng/ml recombinant human (rh)IFNγ, 48hr prior to FC staining. For intracellular staining (ICS) of CXCL10, SC-islets were also treated with 2μM monensin for 6hrs (BioLegend, #420701) to block secretion.

Intracellular staining

Differentiated SC-islet clusters, sampled from suspension cultures (1–2 ml), were dissociated using TrypLE Express (Gibco, 12604013) at 37 °C, mechanically disrupted to form single cells, fixed using 1% paraformaldehyde (PFA) overnight and stored at 4 °C. For staining, fixed single cells were incubated in Perm/Wash Buffer (BD Biosciences, 554723) for 30 min at room temperature, then incubated in Perm/Wash Buffer with primary antibodies (1 h at room temperature), washed three times with Perm/Wash Buffer, incubated with secondary antibodies in Perm/Wash Buffer (1 h at room temperature), washed three times and resuspended in Perm/Wash Buffer.

Surface marker staining

PBS containing 4% Fetal Bovine Serum (FBS) was used as blocking and staining buffer. Immune cells or other dissociated single cells were washed and blocked with blocking buffer for 30 min at 4 °C, then incubated in blocking buffer with conjugated antibodies (1h at at 4 °C), washed three times with blocking buffer, fixed using 1% PFA overnight and stored at 4 °C.

Human lymphocyte staining from mouse whole blood

PBS containing 2% FBS was used as blocking and staining buffer. Cell suspensions from whole blood were washed with blocking buffer and preincubated with Mouse BD Fc Block™ (anti CD16/CD32 Ab, BD Biosciences). Cells were then incubated in blocking buffer with conjugated antibodies (1h at at 4 °C), washed with blocking buffer and fixed with BD FACS lysing solution (BD Biosciences) to lyse red blood cells. For analysis of human immune cells murine cells were identified and excluded by staining with anti-murine CD45 Ab. %CD4+ and %CD8+ were gated from hCD45+/hCD3+

Immunofluorescence Microscopy

Differentiated clusters or tissues were fixed in 4% PFA for overnight at 4 °C, transferred to 30% sucrose overnight, frozen in OCT (Tissue-Tek) and cryostat sectioned. For staining, slides were incubated in CAS block (ThermoFisher, 008120) with primary antibody overnight at 4 °C, washed three times, incubated in secondary antibody for 2 h at room temperature, washed, mounted in ProLong Diamond Antifade Mountant with DAPI, covered with coverslips and sealed with clear nail polish. Representative regions were imaged using Zeiss.Z2 with Apotome microscope.

Magnetic enrichment using CD49a

Stage 6 SC-islet clusters were dissociated using TrypLE Express for 20 min at 37°C. Cells were then quenched with S3 + 10% FBS and spun down. Remaining undissociated cell clusters were mechanically dissociated using a P1000 pipette. The dissociated single cells were resuspended in sorting buffer (PBS + 1% BSA + 2 mM EDTA) and filtered through a 37-µm mesh filter. Cells were counted and resuspended at a density of 10 million cells per 300 µL in 15 mL conical tubes. Cells were stained at room temperature for 20 min using a 1:100 dilution of anti-human CD49a PE-conjugated (BD Biosciences) antibody, covered from light and agitated every 3 minutes. Stained cells were washed twice with 15 mL of sorting buffer by spinning down (5 min, 300 g) and resuspended to their initial density of 10 million cells per 300 µl. To label with microbeads, 40 µL of anti-PE UltraPure MACS microbreads (Miltenyi 130-105-639) were added for each 10 million cells, and the cell solution was incubated for 15 min at 4°C, agitated every 5 min. The stained cells were washed twice as above and resuspended to a target density of 25–30 million cells per 500 µl. Volumes of 500 µL (containing no more than 30 million cells) were then magnetically separated on LS columns (Miltenyi 130-042-401) in a QuadroMACS separator (Miltenyi 130-090-976) using the recommended protocol. Successful PE enrichment was verified by live-cell flow cytometry on an Attune NxT (Invitrogen) flow cytometer.

Human primary immune cell isolation and co-culture assays

We obtained healthy donors' blood derived apheresis collars from Brigham and Woman's Hospital. Human PBMCs were isolated using the density gradient medium, Ficoll-Paque Plus (GE health care life sciences, 17144002) and the SepMate tubes (Stem Cell Technologies, 85450); T cells and NK cells were isolated using RosetteSep Human T Cell Isolation Kit (Stem Cell Technologies, 15061 and 15065, respectively). PBMCs or isolated cells were cultured in T-cell media: X-VIVO 10 (Lonza, 04-380Q) media supplemented with 5% Human AB Serum (Valley Biomedical, HP1022HI), 5% Fetal Bovine Serum (ThermoFisher Scientific, A3840101), 1% Penicillin/Streptomycin (ThermoFisher Scientific, 15070063), GlutaMAX (ThermoFisher Scientific, 35050061), MEM Non-Essential Amino Acids (ThermoFisher Scientific, 11140050).

For co-culture assays, SC-islets were used as target cells. SC-islet clusters were dissociated using TrypLE Express (Gibco, 12604013) at 37 °C, mechanically disrupted to form single cells, ten thousand cells were plated per well on 96-well V-shaped bottom plates and allowed to reaggregate for 48hrs in S3 media. SC-islets were then treated with antibodies (as described) and/or thapsigargin, 5uM (Sigma Aldrich, T9033) for 5 hours before the co-culture assay. Cells were washed to remove residual thapsigargin and Immune cells (PBMCs/T-cells/NK), pre-labeled with Cell Trace Violet (CTV; ThermoFischer Scientific, C34571) were added at a ratio of 5:1 (immune;target cells) in T-cell media.

T cell activation and proliferation assays

After a 48 hours co-culture, the PBMCs/T-cell cell fraction was removed from top cell suspension (SC-islet cluster settle in the bottom). A portion of the cells were taken for flow cytometry staining for CD3+ T cells and activation markers CD69 and CD25. Results are presented as Medial Fluorescent Intensity (MFI), adjusted to baseline MFI of T-cells in an unstimulated PBMC control. The other portion was seeded on 96-well low adherence round bottom plates and allowed to expand for 7-days in T-cell media containing 20 U/ml rhIL-2. Cells were taken for flow cytometry staining and analysis of CD3+ T cells gated cells, while the frequency of CTV negative cells served as a marker for proliferated cells. PBMCs/T cells activated with Dynabeads Human T-Activator CD3/CD28 beads (ThermoFisher Scientific, 111.61) for 48 hours were used as positive control for both assays.

NK cell activation/degranulation assays

During co-culture of SC-islets and NK cells, a CD107a antibody was added to bind internalizing degranulation marker CD107a. After a 48 hours co-culture, the NK cell fraction was removed from top cell suspension and taken for flow cytometry staining and analysis of CD56+CD107a+ cells.

SC-islet and SC-β apoptosis assays

After a 48 hours co-culture, the SC-islet clusters were dissociated using TrypLE Express at 37 °C, to form single cells, fixed O.N. with 1% formaldehyde (CytoFix; Fisher Scientific, BDB554655) and then stained for flow cytometry using C-peptide antibody and terminal deoxynucleotidyl transferase dUTP nick end labeling (TUNEL) for apoptosis with the In Situ Cell Death Detection Kit (Roche Diagnostics, 12156792910). Percent apoptosis was calculated relative to baseline TUNEL staining of SC-islets with no PBMCs.

Tissue/cell preparation and library preparation for single cell RNA sequencing

For graft extraction, mice were euthanized, and the transplanted kidney was removed. SC-islets graft was peeled off the kidney, was sliced into small pieces and digested in 2mg/mL collagenase D (Sigma, 11088858001) in RPMI (GIBCO, 11875-085) for 45 min in 37 °C. Additional breaking was used by pipetting and by filtering through a 40μm cell strainer. Cells were then centrifuged and resuspended in 0.5% BSA in PBS and a magnetic mouse depletion kit (Miltenyi, 130-104-694) was used to remove residual mouse cells.

For co-cultured experiment, cells were collected from 96 wells after 48hrs of co-cultured and SC-islets dissociated using TrypLE Express at 37 °C, to form single cells and quenched with S3 media.

Harvested cells from both sources were centrifuged, resuspended in 0.04% BSA in PBS, counted (LUNA-FX7 Automated Cell Counter), adjusted to 1000 cells/μl and sent to the Harvard University Bauer Sequencing Core for 10X Chromium Single Cell 3’ Library preparation and sequencing.

All samples were loaded into Chip G per the user guide from 10x Genomics, no alterations were made at any step of the protocol (Part No. CG000315). GEMs were formed targeting 10,000 cells and reverse transcription completed immediately after. The cDNA was cleaned from the GEM reagents, amplified for a total of 11 cycles and verified via TapeStation (Agilent Technologies). Amplified cDNA was diluted and ran on the 4200 TapeStation instrument using High Sensitivity D5000 tape and reagents (Part No. 5067-5592 & 5067-5593). The amplified cDNA was fragmented, end repaired, and A-tailed followed by adaptor ligation, and PCR amplification for a total of 12 cycles with each sample receiving a unique set of dual indices (Part No. 1000215). Final libraries were diluted and ran using the High Sensitivity D5000 tape and reagents (Part No. 5067-5592 & 5067-5593) on the 4200 TapeStation (Agilent Technologies). Libraries were quantified via Kapa qPCR using the Complete Universal Kit (Part No. 07960140001, Roche Sequencing Solutions) and the CFX96 Touch Real-Time PCR Detection System (Bio-Rad Laboratories). Libraries were sequenced on an Illumina NovaSeq instrument using the parameters outlined in the user guide (Read1: 28 bp, i7 index: 10 bp, i5 index: 10 bp, Read2: 90 bp).

In vivo **single cell RNA sequencing analysis**

Raw sequencing files were processed using Cell ranger 5.0.0 (10X Genomics). Illumina basecall files were converted to fastq format. Samples were aligned to the GRCh38 genome using STAR aligner (Dobin et al., 2013). Graft samples were also aligned to a GRCh38/mm10 hybrid genome to obtain the human/mouse percentage for each cell. Cells with less than a 50% of aligned reads mapping to the human reference, were discarded.

Processed scRNASeq data was analyzed in R version 4.0.3 using the Seurat suite version 4.0.6 (Hao et al., 2021). Count matrices were loaded into a Seurat object filtering out genes detected in less than 3 cells and cells with less than 200 genes. Quality control filtering was adjusted for each sample as indicated in **Supplementary File 2**. A total of 35,647 cells from 14 samples passed these quality control steps (**Supplementary File 2)**. Data normalization and scaling were performed using Seurat's SCTransform function adding the mitochondrial percentage as an additional regressed variable.

Samples were integrated in two ways. Grafts injected with PBMCs (hPi) and graft samples without injection (control) were integrated to assess the graft component. hPi samples and control PBMC samples were integrated to explore differences in the immune component. In both cases, samples were integrated following the default integration guidelines for SCT transformed datasets from https://satijalab.org/seurat/articles/integration_introduction.html, with 3000 variable features.

To explore transcriptional heterogeneity and to perform initial cell clustering, principal component analysis and nonlinear dimensional reduction using Uniform Manifold Approximation and Projection (UMAP) (McInnes et al., 2018) were applied using 40

dimensions and resolution 1.0 for the integrated hPi and control samples, and 30 dimensions and 0.8 resolution for the integrated hPi and PBMCs. Cell types were assigned to clusters using known marker genes.

Differential expression at the gene level between sample types was performed with DESeq2 1.30.1 (Love et al., 2014) (https://bioconductor.org/packages/DESeq2/) using a pseudobulk approach where counts are aggregated for each cluster at the sample level.

Seurat's plotting functions were used to obtain violin plots. Tidyverse packages (Hadley Wickham (2017). tidyverse: Easily Install and Load the 'Tidyverse'. R package version 1.2.1. https://CRAN.R-project.org/package=tidyverse) were used for data processing.

The distribution of the SC alpha, SC_beta and SC_EC clusters were further analyzed. By selecting the cells from these clusters and reclustering them. Principal component analysis and nonlinear dimensional reduction using UMAP (McInnes et al., 2018) were applied using 10 dimensions and resolution 0.1.

In vitro single cell RNA sequencing analysis

Raw sequencing files were processed using Cell ranger 5.0.0 (10X Genomics). Illumina basecall files were converted to fastq format. Samples were aligned to the GRCh38 genome using STAR aligner (Dobin et al., 2013).

Processed scRNASeq data was analyzed in R version 4.0.3 using the Seurat suite version 4.0.6 (Hao et al., 2021). Count matrices were loaded into a Seurat object filtering out genes detected in less than 3 cells and cells with less than 200 genes. During quality additional filtering was used. Cells with less than 1000 genes, less than 3000 UMIs or with a higher mitochondrial percentage than 15%, were discarded. A total of 42,922 cells from 8 samples passed these quality control steps . Data normalization and scaling were performed using Seurat's SCTransform function adding the mitochondrial percentage as an additional regressed variable. All samples were integrated using Harmony (Korsunsky et al., 2019) regressing the time point variable.

To explore transcriptional heterogeneity and perform initial cell clustering, principal component analysis and nonlinear dimensional reduction using UMAP (McInnes et al., 2018) were applied using 20 dimensions and resolution 0.3. Cell types were assigned to clusters using known marker genes. An evaluation of the UMAP plots showed that 61 cells had been overcorrected (i.e: endocrine cells in the PBMCs only samples or vice versa) during the integration and were removed from the analysis.

For differential expression and dot plot generation, data was also processed using Scanpy (version 1.8.1) (Wolf et al., 2018) to annotate the cell types. Cell ranger output was filtered to retain cells with no more than 15% MT transcripts and 4000 highly variable genes were identified using the highly_variable_genes function with the Seurat v3 option.

Cell types were assigned to clusters using known marker genes. Results of identified cell types were then visualized using dotplot plotting functions.

Using the annotations, differential expression was measured using fold change between treatment conditions and the Mann-Whitney hypothesis test p-values after correction for multiple comparisons using the FDR procedure implemented in the multipletests function from the statsmodels library.

Lentivirus Preparation and Transduction

Lentiviral particles were produced by transfecting 293T cells (Takara Bio) with the packaging vectors pHDM-vsvg, pHDM-tat, pHDM-rev, and pHDM-gag/pol along with lentiviral backbone vectors using the TransIT-293 transfection reagent (Mirus). Lentiviral particles were collected 48 h and 72 h post transfection and concentrated using the PEG-IT virus precipitation reagent (Fisher Scientific, Waltham, MA, USA) overnight at 4 °C followed by centrifugation at 1500 g for 30 min at 4 °C and stored at −80 °C.

The lentiviral vector lentiCRISPRv2 [a gift from Feng Zhang (Addgene plasmid # 52961 ; http://n2t.net/addgene:52961; RRID:Addgene_52961 (Sanjana et al., 2014)] was used to clone guide RNAs (gRNA sequences described in Key resource table; custom clone service from GensScript). For overexpression, lentiviral vectors containing open reading frame (ORF) sequences of eGFP, *CD274*, *CXCL10* and *SOCS1*, cloned into pLX_307, were obtained from the Broad institute inventory.

For transduction, cell clusters collected from spinner flask suspension cultures were dissociated in TrypLE Express (Life Technologies) for 7 min, followed by mechanical dissociation and centrifugation at 300 g for 5 min at room temperature (RT). Cell pellets were resuspended at a density of 2.5 million cells/mL in the stage-matched medium with polybrene reagent (Santa Cruz) at 10 µg/mL. Single-cell suspensions were combined with concentrated lentiviral particles and allowed to reaggregate in spinner flasks, in a humid 37 °C incubator and 5% CO_2.

Whole genome CRISPR screen in vivo and analysis

Brunello pooled library pooled plasmid DNA in a 1 vector system (lentiCRISPRv2 backbone; Addgene # 73179) was obtained from the Broad institute Genetic Perturbation Platform (GPP), to generate pooled lentivirus. Lentivirus and SC-islet transduction was as described above. To determine a titer that will lead to a multiplicity of infection (MOI) that is less than 1, SC-islets were seeded in 6 well plates and treated with different virus volumes per cell number. After 2-3 days transduced SC-islets were treated with puromycin (9µg/ml) and cell counts were taken after 4 days to evaluate cell death ratios compared to a control well with no selection.

Library transduced (LT) SC-islets were transplanted under the kidney capsule of NSG-MHC^{null} nice and PBMCs were injected at week 4 after transplantation. Full experiment layout is described in the results section and in Figure 1**.** Retrieved graft tissue were homogenized (Polytron PT 1200E, KINEMATICA) and a Quick-DNA™ Midiprep Plus Kit

(Zymo Research, D4075) was used to extract genomic DNA (gDNA). gDNA was submitted to GPP for PCR amplification of the integrated construct containing a barcode sequence, and Illumina sequencing to determine the abundance of each gRNA in each sample. Sequencing resulted in 132,183,231 matching reads which consists of a 82% of total reads. PoolQ v3 software was used to deconvolute sequencing files and quantitate gRNA barcodes counts in each sample (https://portals.broadinstitute.org/gpp/public/software/poolq).

To identify genes that may influence graft depletion, we estimate the gene by environment interactions of KO allele targets and PBMC graft environments (hPi), which can be interpreted as a difference in PBMC depletion between the KO and WT alleles. A separate model is fit for each of the target genes using observed sequenced read counts as the outcome and all available data across mouse and guide replicates. Read counts are modeled as negative binomial, with additive random effects for targeting guide and mouse, and fixed effects representing the graft allele and condition. The full model is given by:

log(counts) ~ 1+KO+PBMC+KO*PBMC+(1|Mouse)+(1|Guide)

Significance of the KO*PBMC interaction is evaluated using a likelihood ratio test comparing with a reduced model that only includes the additive effects. All of the models are fit using the glmer.nb function from the lme4 R package. To correct for multiple comparisons, p-values are adjusted using the Benjamini-Hochberg FDR procedure, implemented in the R function p.adjust with option ‘fdr’. A filter was applied to remove any gene hits that were not expressed in at least 10% of either SC-α or SC-β cells in at least one experimental condition across *in vivo* and *in vitro* scRNA-seq experiments (Figures 1 and 2). To visualize the results from a subset of selected CRISPR targets, we produced boxplots of the full model predictions for each allele and treatment combination.

Generation of hESC knockout lines

Gene modified (GM) lines were generated by homology directed repair (HDR), via nucleofection of a Cas9/sgRNA ribonucleoprotein complex (RNP) and a targeting vector. The targeting vector (OriGene) was designed to facilitate the in-frame integration of GFP or luciferase cassettes with puromycin resistance into exon 2 or exon 3 of the CXCL10 or STAT1 loci, respectively (**Figures 4A and 4B**). Culture and expansion were performed on Matrigel® (Corning) coated plates with mTeSR™ Plus media (Stem Cell Technologies). Cells were clump passaged every 72h or 96h. For nucleofection Hues8 monolayers were dissociated into single cells with Accutase (Stem Cell Technologies), and $1x10^6$ cells were nucleofected using the 4D-Nucleofector (Lonza) with 5 µg targeting plasmid and RNP (120 pmol targeting sgRNA and 104 pmol Alt-R Cas9 (IDT), according to the manufacturer’s instructions. Nucleofected cells were then plated in a matrigel-coated tissue culture plate containing mTeSR™ Plus, cloneR (Stem Cell Technologies) and 7.5 µM RS-1 (Xcessbio). After 48h, puromycin (0.5µg/ml) was added and surviving colonies were transferred to 96-well plates for PCR and expansion. Genomic DNA was

extracted and purified using Quick-DNA™ (Zymo Research), and target cassette knock-in was confirmed by PCR analysis (**Figure S4A**) using Phusion® Hot Start Flex 2x master mix and primer sets (see key resource table) that amplify the wild type and targeted genomic alleles (blue and red arrows, respectively in **Figure 4A and 4B**). Several heterozygous clones were acquired from each knockout, and we selected a CXCL10-GFP (C10G) clone and a STAT1-luciferase (ST1L) clone that contained the integrated transgene in one allele along with a nonhomologous end-joining (NHEJ) mutation in the intact endogenous allele, determined by PCR and Sanger (GENEWIZ) sequencing (**Figure S4A**). Overall, C10G and ST1L contained null mutations in both allelles.

QUANTIFICATION AND STATISTICAL ANALYSIS

Statistical analysis was performed by unpaired Student's *t*-tests as indicated, using the Prism v9. All data are presented mean ± SD. $p<0.05$ was considered statistically significant. Sufficient sample size was estimated without the use of a power calculation. Data analysis was not blinded.

GRAPHIC ILLUSTRATIONS

Graphic illustrations in the manuscript were created with BioRender.com under BioRender's Academic License Terms.

DATA AVAILABILITY

scRNA-seq and pooled CRISPR screen data generated during this study are available at NCBI GEO accession number GSE200104, and composed of listed SubSeries related to specific experiments described in this paper.

REFERENCES

Dobin, A., Davis, C.A., Schlesinger, F., Drenkow, J., Zaleski, C., Jha, S., Batut, P., Chaisson, M., and Gingeras, T.R. (2013). STAR: ultrafast universal RNA-seq aligner. Bioinformatics 29, 15-21.

Hao, Y., Hao, S., Andersen-Nissen, E., Mauck, W.M., 3rd, Zheng, S., Butler, A., Lee, M.J., Wilk, A.J., Darby, C., Zager, M., et al. (2021). Integrated analysis of multimodal single-cell data. Cell 184, 3573-3587 e3529.

Korsunsky, I., Millard, N., Fan, J., Slowikowski, K., Zhang, F., Wei, K., Baglaenko, Y., Brenner, M., Loh, P.R., and Raychaudhuri, S. (2019). Fast, sensitive and accurate integration of single-cell data with Harmony. Nat Methods 16, 1289-1296.

Love, M.I., Huber, W., and Anders, S. (2014). Moderated estimation of fold change and dispersion for RNA-seq data with DESeq2. Genome Biol 15, 550.

McInnes, L., Healy, J., and Melville, J. (2018). UMAP: Uniform Manifold Approximation and Projection for Dimension Reduction, pp. arXiv:1802.03426.

Pagliuca, F.W., Millman, J.R., Gurtler, M., Segel, M., Van Dervort, A., Ryu, J.H., Peterson, Q.P., Greiner, D., and Melton, D.A. (2014). Generation of functional human pancreatic beta cells in vitro. Cell 159, 428-439.

Sanjana, N.E., Shalem, O., and Zhang, F. (2014). Improved vectors and genome-wide libraries for CRISPR screening. Nat Methods 11, 783-784.

Veres, A., Faust, A.L., Bushnell, H.L., Engquist, E.N., Kenty, J.H., Harb, G., Poh, Y.C., Sintov, E., Gurtler, M., Pagliuca, F.W., et al. (2019). Charting cellular identity during human in vitro beta-cell differentiation. Nature 569, 368-373.

Wolf, F.A., Angerer, P., and Theis, F.J. (2018). SCANPY: large-scale single-cell gene expression data analysis. Genome Biol 19, 15.
